# High-Throughput Screening Identifies Covalent Inhibitors with Unique Chemistry and Binding Modes for SARS-CoV‑2 Main Protease

**DOI:** 10.1021/jacsau.6c00371

**Published:** 2026-07-13

**Authors:** Sandeep Atla, Kai S. Yang, Erol C. Vatansever, Eun Jeong Cho, Xinyu Ma, Yugendar R. Alugubelli, Satyanarayana Nyalata, Lauren R. Blankenship, Kaustav Khatua, Demonta Coleman, Xuejiao Guo, Rachel M. Sammons, Dorsa Rabie, Banumathi Sankaran, Tahmina Pervin, Benjamin W. Neuman, Chia-Chuan D. Cho, Lai Hoang Son Le, Alan Pham, Nagarjun R. Mallampudi, Kevin N. Dalby, Shiqing Xu, Wenshe Ray Liu

**Affiliations:** † Texas A&M Drug Discovery Center and Department of Chemistry, College of Arts and Sciences, 14736 Texas A&M University, College Station, Texas 77843, United States; ‡ Targeted Therapeutic Drug Discovery & Development Program, the University of Texas, Austin, Texas 78712, United States; § Division of Chemical Biology & Medicinal Chemistry, College of Pharmacy, the University of Texas, Austin, Texas 78712, United States; ∥ Molecular Biophysics and Integrated Bioimaging, Berkeley Center for Structural Biology, 1666 Laurence Berkeley National Laboratory, Berkeley, California 94720, United States; ⊥ Department of Pharmaceutical Sciences, Irma Lerma Rangel College of Pharmacy, Texas A&M University, College Station, Texas 77843, United States; # Institute of Biosciences and Technology and Department of Translational Medical Sciences, School of Medicine, Texas A&M University, Houston, Texas 77030, United States; ∇ Department of Biochemistry and Biophysics, College of Agriculture and Life Sciences, Texas A&M University, College Station, Texas 77843, United States; ○ Department of Cell Biology and Genetics, School of Medicine, Texas A&M University, College Station, Texas 77843, United States; ◆ Department of Biology, College of Arts and Sciences, Texas A&M University, College Station, Texas 77843, United States

**Keywords:** SARS-CoV-2, Main Protease, Covalent Inhibitor, Nucleophilic Aromatic Substitution, Nucleophilic Acyl Substitution

## Abstract

Using an easily scalable SARS-CoV-2 main protease (M^Pro^) substrate, high-throughput screening of ∼75,000 compounds drawn from diversity and fragment libraries was conducted. Based on screening results, 40 inhibitors were confirmed with IC_50_ values below 30 μM. Four hits were revalidated, and their M^Pro^ complexes were characterized by X-ray crystallography. For two inhibitors, CDI5573 and CDI8009, clear active-site electron density revealed covalent modification of Cys145, the enzyme’s catalytic cysteine. In both structures, His41 of the catalytic dyad reorients from its native hydrogen-bonding pose with Cys145 to engage in π-stacking with the inhibitors’ aromatic rings. The two covalent adducts arise from distinct chemical mechanisms. CDI5573, which is a 3-nitro-1,2,5-oxazole derivative, reacts through nucleophilic aromatic substitution (S_N_Ar) to replace one of the nitro groups by Cys145, whereas CDI8009 undergoes nucleophilic acyl substitution to form a thioester with Cys145. Structure-guided optimization of CDI5573 yielded new analogs, including one inhibitor with an IC_50_ of 15 nM, placing this molecule among the most potent inhibitors developed for M^Pro^. These findings introduce new covalent chemotypes with diverse modes of engagement for M^Pro^, expand the accessible space for this essential viral enzyme, provide leads with the potential to complement existing COVID-19 therapeutics and mitigate viral resistance, and guide the targeting of other cysteine enzymes for drug discovery.

## Graphical abstract

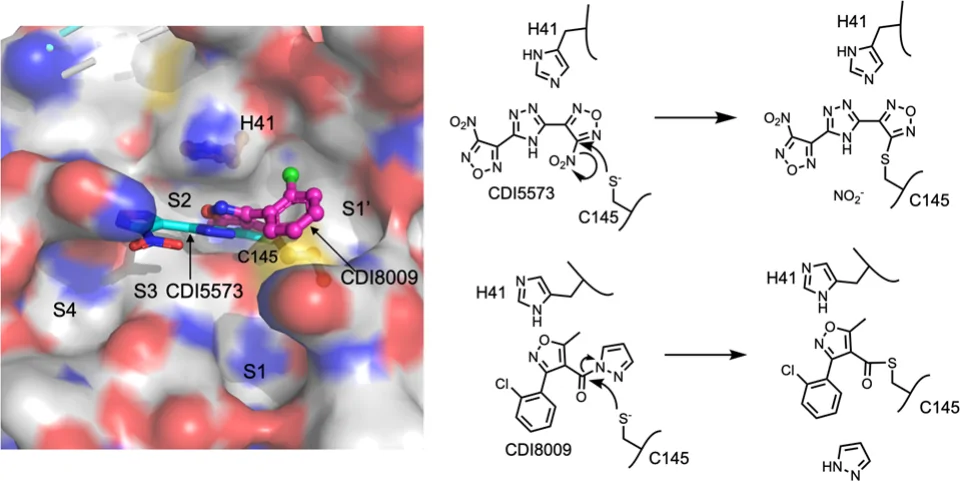

COVID-19 is an infectious disease that emerged in late 2019 and rapidly spread worldwide.^1^ To date, nearly 800 million cases and more than 7 million deaths have been reported.^2^ Although the acute pandemic phase has receded, the disease persists endemically, with periodic surges. The COVID-19 pathogen, SARS-CoV-2, is an enveloped, positive-sense β-coronavirus (CoV). Upon infection of human cells, the viral genomic RNA is translated into two polypeptides, pp1a and pp1.^3^ These two polypeptides are cleaved by two internal cysteine protease fragments nsp3 and nsp5 to generate a number of mature nonstructural proteins (nsps). Some of these nsps assemble into a replication-transcription complex to synthesize genomic and subgenomic RNAs. Because they are indispensable for polypeptide processing, nsp3 and nsp5 are prime antiviral targets. Clinical validation has been achieved for nsp5 with approved inhibitors, including nirmatrelvir (used with ritonavir) and ensitrelvir.^4^

Nsp5, also known as main protease (M^Pro^) or 3C-like protease (3CL^Pro^), is a cysteine protease with a catalytic dyad comprising Cys145 and His41. The enzyme is highly conserved. M^Pro^ from SARS-CoV and SARS-CoV-2 shares ∼ 96% sequence identity and is broadly conserved across CoV.^5^ Given the recurrence and lethality of major CoV outbreaks over the past two decades (SARS-CoV, MERS-CoV, and SARS-CoV-2), M^Pro^ is a compelling target for pan-CoV antiviral development. Ongoing innovation is also needed to counteract resistance that can emerge to approved drugs.^6^ Accordingly, expanding the chemical space and binding mode diversity of M^Pro^ inhibitors remains a priority for the development of durable, broad-spectrum CoV therapeutics.

Multiple M^Pro^ substrates have been developed for enzymatic characterization, inhibitor profiling, and high-throughput screening.^7^ In our early work, we evaluated three fluorogenic substrates, Sub1, Sub2, and Sub3, whose structures are shown in Figure 1A, for drug repurposing assays.^8^ Sub3 delivered rapid kinetics and strong fluorescence, but is relatively large, and its synthesis is not easily scalable. Sub1 is much smaller and can be synthesized at scale. However, MPro hydrolyzes it substantially more slowly than Sub3 (Figure 1B). Guided by MPI3,^9^ a potent inhibitor with an IC_50_ of 8 nM and containing a β-(*S*-2-oxopyrrolidin-3-yl)-alanyl (opaa) residue that enhances S1 subsite recognition in the M^Pro^ active site, we designed Sub4, a tripeptidic fluorogenic substrate (structure in Figure 1A) featuring an *N*-CBZ cap, a P1 opaa residue, and a *C*-terminal 7-amino-4-methylcoumarin (AMC) reporter. Under assay conditions of 10 μM substrate and 100 nM M^Pro^, Sub4 is hydrolyzed ∼ 5-fold faster than Sub1 and yields a robust fluorescence signal (Figure 1B). Sub4 is prepared by three straightforward amide couplings from inexpensive, readily available starting materials. Therefore, we scaled up its synthesis and used it for our high-throughput screening campaigns.

**Figure 1:**
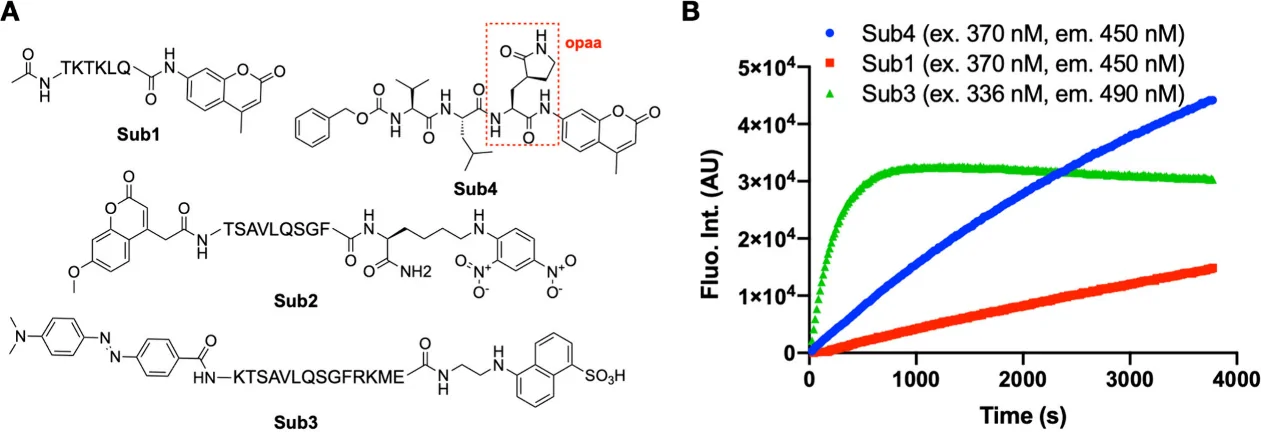
M^Pro^ substrates used for kinetic analysis. (**A**) Structures of four M^Pro^ substrates. β-(*S*-2-oxopyrrolidin-3-yl)-alanyl (opaa) for improved binding to M^Pro^ in Sub4 is highlighted. (**B**) Comparison of catalytic activity of 100 nM M^Pro^ on 10 μM Sub1, Sub3, and Sub4.

We first evaluated the M^Pro^-Sub4 enzyme–substrate pair for high-throughput screening. At Sub4 of 600 nM, the initial hydrolysis rate, measured as detectable fluorescent AMC formation, increased proportionally with M^Pro^ concentration from 0 to 1,000 nM (). At a fixed M^Pro^ concentration (100 nM), reaction velocity was linear across 0–10 μM (), indicating a K_m_ higher than 10 μM for this substrate. This high K_m_ value is critical for avoiding substrate overcompeting with potential inhibitors during high-throughput screening. Under conditions with 10 nM M^Pro^ and 10 μM Sub4, we observed a robust increase in enzyme-catalyzed fluorescence, and the potent inhibitor MPI3 produced dose-dependent inhibition of this fluorescence change, consistent with its reported inhibition potency (). Based on these evaluations, we established a robust high-throughput screening assay using 20 nM M^Pro^ and 10 μM Sub4, with a buffer condition tolerant of DMSO, BSA, and TX-100. This assay was first applied to a 29,669-compound library, followed by an additional 45,169-compound library. Primary screening was performed at 12.5 μM. Compounds showing >15% inhibition were retested at 10 and 50 μM. Hits with >40% inhibition at 50 μΜ were advanced to IC_50_ determination using library materials. In total, 40 inhibitors were identified with IC_50_ < 30 μM (), including 20 with IC_50_ ≤ 10 μM and 2 with submicromolar potency.

Several hits contained 2-chloroacetylamino warheads. Because these electrophiles are known to form covalent adducts with M^Pro^, they were not considered for further investigation in our search for new MPro inhibition mechanisms. We then purchased four commercially available hits with putative covalent mechanisms and retested them using Sub3 as a substrate. Their determined IC_50_ values are presented in Table 1. For CDI0069–0003 and CDI8009–5647, repurchased material exhibited substantially greater potency than the corresponding library stocks. This is likely due to compound degradation and/or differences in the primary assay testing conditions. To define binding modes, all four compounds were evaluated by X-ray crystallography by soaking ligands into pregrown apo-M^Pro^ crystals.^10^ Clear active site electron density was obtained for CDI5573–0017 and CDI8009–5647, enabling confident modeling of the bound ligands. By contrast, CDI0069–0003 and NSC651084 did not yield interpretable ligand density. Both compounds contain a 1,2- or 1,4-benzoquinone electrophile that can react with a cysteine thiol at multiple positions, generating potentially mixtures of adducts. Such heterogeneity likely contributed to the weak/heterogeneous density observed for these two molecules. For CDI5573–0017 and CDI8009–5647, their modeled structures reveal distinct covalent engagement and interaction patterns in the M^Pro^ active site.

**Table 1:**
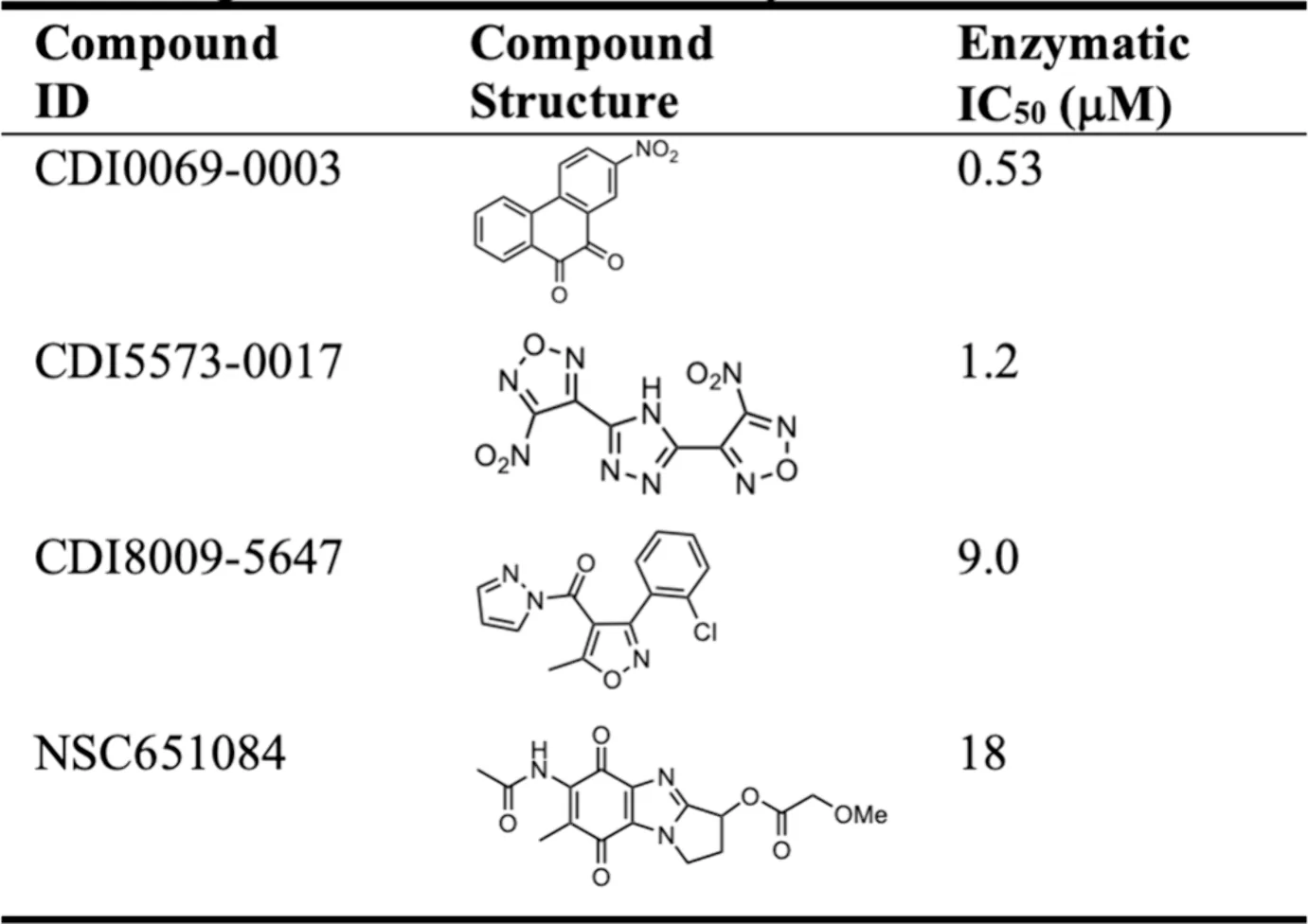
5 Retested M^pro^ Inhibitors from High-Throughput Screening Results and Their Potency

CDI5573–0017 (CDI5573 in short) is 3,5-bis­(4-nitro-1,2,5-oxadizol-3yl)-1,2,4-triazole. In the M^Pro^ active site, the electron density delineates three heterocycles and one terminal oxadiazole conjugated to Cys145 (Figure 2A). Notably, His41 adopts an unusual pose. Its imidazole stacks over the CDI5573 ring system, consistent with a π-π stacking interaction. No electron density is observed for the nitro group at the oxazole ring connecting to Cys145, indicating its displacement by the Cys145 thiol. A relatively weak density consistent with a nitro substituent appears on the oxadiazole distal to Cys145, suggesting a possible flexible conformation mode. These structural features support a nucleophilic aromatic substitution (S_N_Ar) reaction mechanism in which the Cys145 thiol replaces one terminal nitro group on an electron-deficient oxadiazole (Figure 2B). In the literature, another structural confirmed molecule that uses the S_N_Ar mechanism to inhibit M^Pro^ is halicin.^11^ Beyond the covalent Cys145 adduct and a His41 π-π contact, there are four hydrogen bonds formed with CDI5573. The central triazole forms two hydrogen bonds with two water molecules. One water, buried deep in the active site, also hydrogen-bonds to His164. The side chain amide of Gln189 forms two additional hydrogen bonds with CDI5573, one to a ring nitrogen and one to a nitro oxygen on the distal oxadiazole (Figure 2C). The same distal oxadiazole forms van der Waals contacts with the aa187–189 loop. Compared with apo-M^Pro^, His41 shifts away from Cys145 and rotates its side chain by ∼ 90° to establish a π-π stacking interaction with CDI5573 (Figure 2D). The aa187–193 loop moves outward to widen the active site, and the short helix bearing Met49 becomes poorly ordered, indicating increased flexibility. A substantial rearrangement of residues 43–51 is consistent with the requirement to form a characteristic Y-shaped, S–O–N–O-S-bridged cross-link among residues Cys22, Cys44, and Lys61.^12^ This oxidative linkage has been observed in multiple M^Pro^-inhibitor structures.^13^ Superimposing M^Pro^-CDI5573 with the M^Pro^-nirmatrelvir complex (Figure 3E) highlights significantly different binding modes for the two inhibitors. According to the Schechter-Berger nomenclature,^14^ M^Pro^ comprises subsites, S1, S2, S3, S4, and S1’. In the CDI5573 complex, the reorientation of His41 creates an additional deep subsite for CDI5573 binding. Aside from penetrating this newly formed cavity, CDI5573 predominantly occupies S2. In contrast, nirmatrelvir engages multiple subsites except S1’.

**Figure 2:**
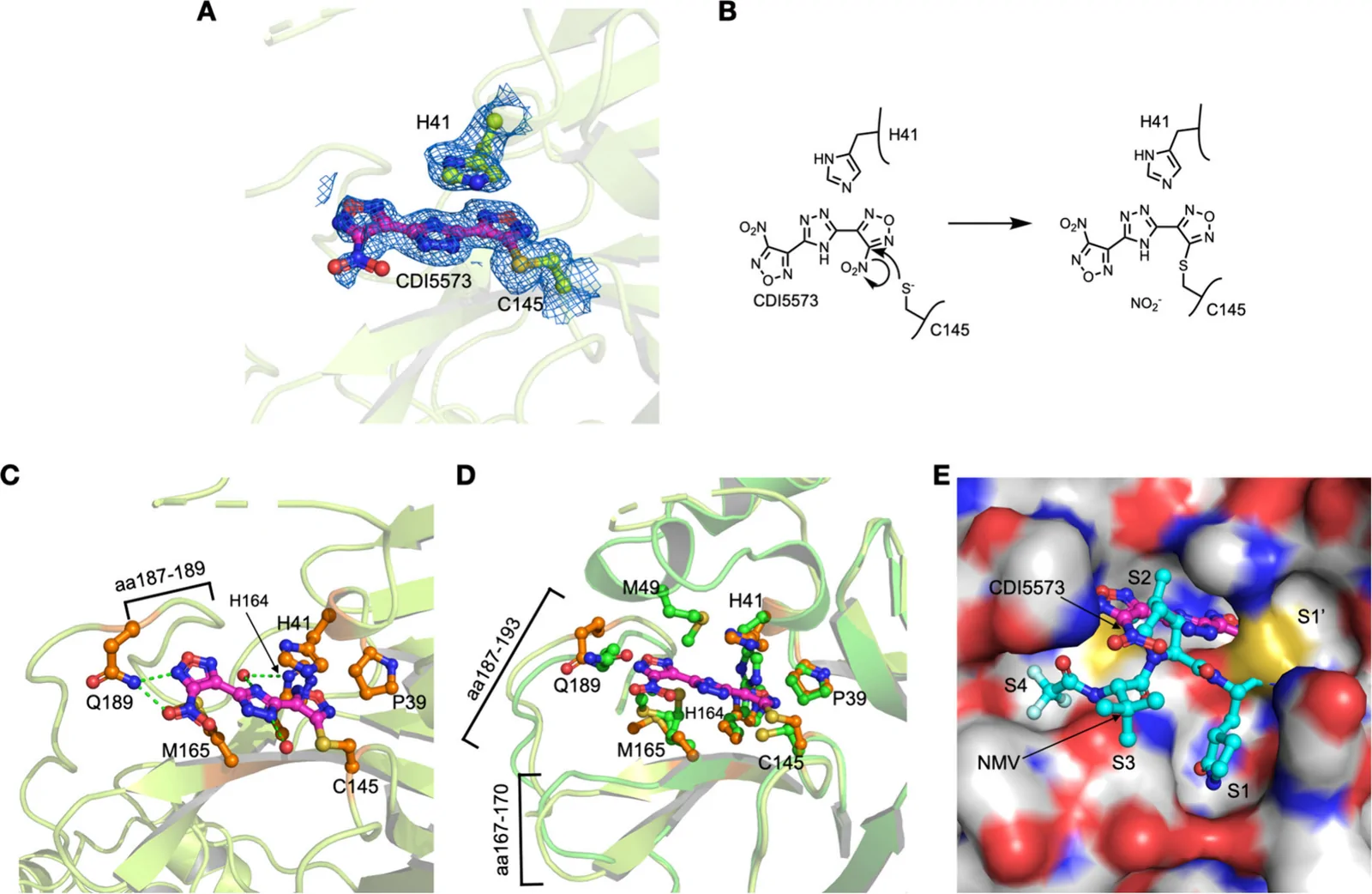
Crystal structure of M^Pro^ bound with CDI5573. (**A**) 2Fo-Fc maps that are colored in blue and contoured at the 1σ level delineate CDI5573 with one of its nitro groups replaced by Cys145 and His41 forming a pi-stacking interaction with CDI5573. (**B**) A proposed nucleophilic aromatic substitution (S_N_Ar) reaction between CDI5573 and the Cys145 thiol to generate a covalent adduct. (**C**) Interactions of CDI5573 in the active site of M^Pro^. Hydrogen bonds involving CDI5573 are depicted as green dashed lines. Carbon atoms of CDI5573 are colored in pink. M^Pro^ residues surrounding CDI5573 are presented as their ball-and-stick models with carbon atoms colored in orange. Secondary structures of M^Pro^ are presented in limon. (**D**) Comparison of M^Pro^-CDI5573 to apo M^Pro^ (pdb entry: 6Y2E). M^Pro^-CDI5573 is presented as same as that in **C**. Apo M^Pro^ is depicted with its second structures and carbon atoms colored in green. (**E**) Comparison of M^Pro^-CDI5573 to M^Pro^-nirmatrelvir (NMV) by superimposing their structures. Surface of M^Pro^ in M^Pro^-CDI5573 is contoured by showing its carbon atoms in light gray. CDI5573 is presented as same as in **C**. NMV is shown with its carbon atoms colored in cyan. Five active site subsites according to the Schechter-Berger nomenclature are labeled. Structure of M^Pro^-NMV is based on pdb entry: 7RFS.

**Figure 3:**
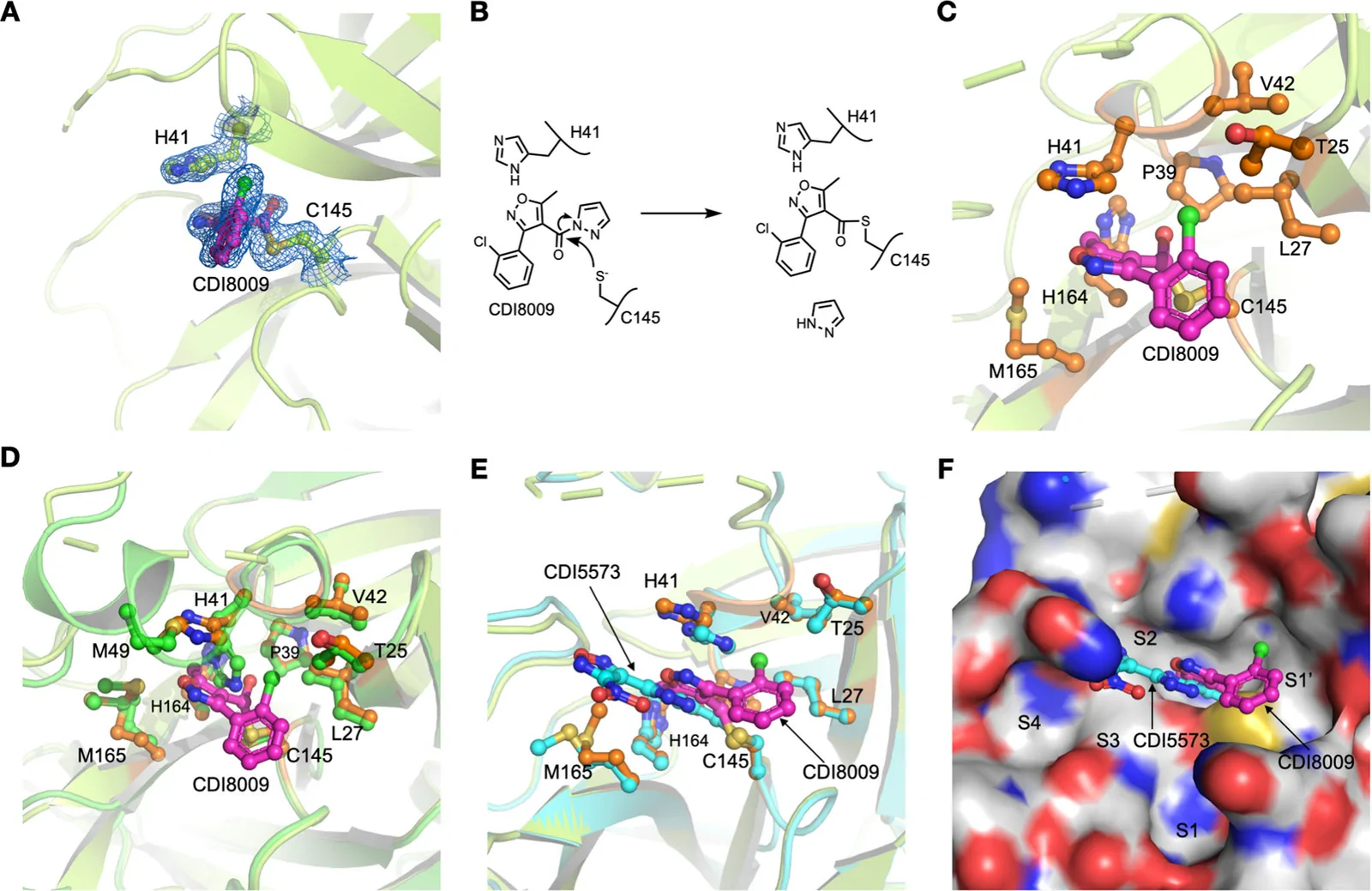
Crystal structure of M^Pro^ bound with CDI8009. (**A**) 2Fo-Fc maps colored in blue and contoured at the 1σ level delineate CDI8009 with its pyrazole covalently replaced by Cys145 and His41 stacking on top of its isoxazole ring. (**B**) A proposed nucleophilic acyl substitution reaction between CDI8009 and Cys145 to form a covalent adduct. (**C**) Interactions of CDI8009 at the active of M^Pro^. No hydrogen bond is formed. Carbon atoms of CDI8009 are colored in pink. M^Pro^ residues surrounding CDI8009 are presented as their ball-and-stick models with carbon atoms colored in orange. Secondary structures of M^Pro^ are presented in limon. (**D**) Comparison of M^Pro^-CDI8009 to apo M^Pro^ (pdb: 6Y2E). Two structures are superimposed. M^Pro^-CDI8009 is presented as same as in **C**. Apo M^Pro^ is shown with secondary structures and carbon atoms in green. (**E**) Comparison of M^Pro^-CDI8009 to M^Pro^-CDI5573. Two structures are superimposed. M^Pro^-CDI8009 is shown as same as in **C**. M^Pro^-CDI5573 is depicted with secondary structures and carbon atoms colored in cyan. (**F**) M^Pro^-CDI8009 and M^Pro^-CDI5573 superimposed structures with the surface of M^Pro^ in M^Pro^-CDI8009 depicted to show five active site subsites. Carbon atoms are colored in light gray to draw the protein surface.

To further confirm the covalent, adduct formation between M^Pro^ and CDI5573, we incubated them together and then applied the protein sample to the electrospray ionization mass spectrometry (ESI-MS) analysis. Compared to free enzyme that had a determined molecular weight of 33,855 Da, the M^Pro^-CDI5573 complex showed a detected molecular weight of 34,056 Da (). The molecular difference is 201 Da, corresponding to the covalent replacement of both nitro groups by M^Pro^ residues. Although our crystal structure of M^Pro^-CDI5573 showed only one covalent bond, it is likely that in solutions, M^Pro^-CDI5573 allows structural rearrangement to allow CDI-5573’s second nitro group to react with another nucleophilic residue. Nevertheless, the ESI-MS results confirmed the formation of a covalent adduct between M^Pro^ and CDI5573.

CDI8009–5647 (CDI8009, abbreviated) is an acylpyrazole. Its labile acyl-pyrazole bond can undergo nucleophilic acyl substitution with the M^Pro^ catalytic Cys145 to form a thioester. This adduct mimics the acyl-enzyme intermediate generated during peptide cleavage by M^Pro^, wherein Cys145 transiently forms a thioester with the substrate’s scissile carboxyl before hydrolysis regenerates the Cys145 thiol. The central question, therefore, is why CDI8009 can potently inhibit M^Pro^ instead of undergoing a two-step reaction, first to a thioester intermediate that then hydrolyzes, to serve as a substrate. Our X-ray crystallography analysis revealed electron density in the M^Pro^ active site that unambiguously resolves the covalently attached 3-(2-chlorophenyl)-5-methylisoxazole-4-carbonyl fragment (Figure 3A). This is consistent with a mechanism presented in Figure 3B. Notably, His41 adopts an orientation in which its imidazole side chain forms a π-π stacking interaction with the CDI8009 isoxazole. This type of thioester bond formation and His41 reorientation have been observed with several small-molecule thioester inhibitors.^15^ In the enzyme catalysis, His41 serves as a base to activate a water nucleophile for thioester hydrolysis. Here, no ordered water density is observed in the vicinity of His41, and its reoriented, stacking conformation is incompatible with acting as a general base. The generated thioester bond is also deeply buried and sterically shielded. As shown in Figure 3C, the 2-chlorophenyl substituent packs tightly against Thr25, Leu27, Pro39, His41, and Val42, filling the local cavity and occluding solvent access to the thioester carbonyl. Together, suppression of His41’s base function and solvent exclusion rationalize the stabilization of the acyl-enzyme thioester and the observed potent inhibition by CDI8009.

Compared to apo-M^Pro^, significant conformational changes are observed in His41 and in the Met49-bearing peptide region (Figure 3D). His41 reoriented from its dyad position for hydrogen bonding with Cys145 into a pose that forms a stacking interaction with the CDI8009 isoxazole. The imidazole side chain also undergoes a significant conformational change. The Met49-bearing peptide region becomes poorly ordered, and a rearrangement of residues 43–51 accompanies formation of the characteristic S–O–N–O-S-bridged cross-link among Cys22, Cys44, and Lys61. Overall, M^Pro^-CDI8009 adopts protein conformations that are highly similar (Figure 3E). His41 assumes a comparable orientation for stacking over the ligand in both complexes, albeit slightly displaced farther from Cys145 in M^Pro^-CDI8009 to accommodate the chlorine atom in CDI8009.

This type of His41 reorientation has also been described in M^Pro^ bound with shikonin and ensitrelvir.^16^ A notable difference involves Met165. Its εmethyl group points toward S2 in M^Pro^-CDI8009 but toward S4 in M^Pro^-CDI5573. Superimposing the two structures, with the protein surface as in Figure 3F, underscores distinct inhibitor-binding modes. In each case, the reorientation of His41 creates a deep subsite to accommodate an aromatic ring from the inhibitor while forming a covalent adduct with Cys145. CDI8009’s 2-chlorophenyl group packs tightly in S1’, whereas CDI5573 engages S2 predominantly. These interaction patterns are different from those of most reported M^Pro^ inhibitors. CDI8009 shares mechanistic similarity with the previously reported indole chloropyridinyl ester-derived SARS-CoV-2 M^Pro^ inhibitors,^19^ as both inhibitor classes undergo nucleophilic acyl substitution with Cys145 to form covalent thioester adducts. Notably, our crystallographic analysis reveals that CDI8009 adopts a distinct binding mode featuring pronounced His41 reorientation and tight shielding of the thioester carbonyl from solvent, which may contribute to stabilization of the acyl-enzyme intermediate.

Because the 3-(2-chlorophenyl)-5-methylisoxazole-4-carbonyl fragment of CDI8009 shields the thioester carbonyl from solvent in the final M^Pro^-CDI8009 complex, a reverse process for a pyrazole to add to this thioester is precluded as well. Therefore, the parent molecule must engage the enzyme in a different, preacylation pose before forming the thioester and only then rearrange to the observed binding mode. This required binding-mode shift complicates the structure-based optimization of CDI8009. We therefore prioritized CDI5573 for a structure–activity relation (SAR) campaign to improve potency. For CDI5573 to react productively with Cys145, the likely sequence involves His41 reorientation to a π-stacked conformation with CDI5573, binding of CDI5573 to S2, and alignment of the oxadiazole with the leaving nitro group for reaction with Cys145. Comparison of M^Pro^-CDI5573 with Apo-M^Pro^ shows that the aa187–189 loop moves outward to widen the active site to accommodate CDI5573, indicating a potential steric repulsion between its apo conformation and the incoming ligand. Guided by these features, we designed and synthesized 13 new 3-nitro-1,2,5-oxadiazole analogs, as shown in Table 2, that are overall smaller than CDI5573. SR-B-163, bearing an amine in place of the nitro leaving group, served as a negative control. Enzymatic inhibition assays showed that SR-B-163 was not inhibited, whereas all three 3-nitro-1,2,5-oxadiazole analogs inhibited M^Pro^ to varying extents. Among 13 new inhibitors, 10 have IC_50_ ≤ 1 μM and 2 have IC_50_ < 100 nM. Except the three chlorophenyl-substituted inhibitors, SR-C-44, SR-C-46, and NS-A-136, and a tetrazine-substituted analog SR-C-40, all others surpass the potency of CDI5573. The most potent molecule, SR-B-182, has an IC_50_ of 15 nM. This strong inhibitory potency ranks SR-B-182 among the most potent M^Pro^ inhibitors developed to date.^15,17^ SR-B-182 closely resembles CDI5573 but avoids steric clash with the aa187–189 loop. Its higher potency than CDI5573 validates our structure-based design. NS-A-136 is the second most potent inhibitor with an IC_50_ of 93 nM. Its high potency is likely due to the strong electron-withdrawing effect of 3-bromopyridine-1-oxide, which further activates 3-nitro-1,2,5-oxadiazole toward reaction with Cys145.

**Table 2:**
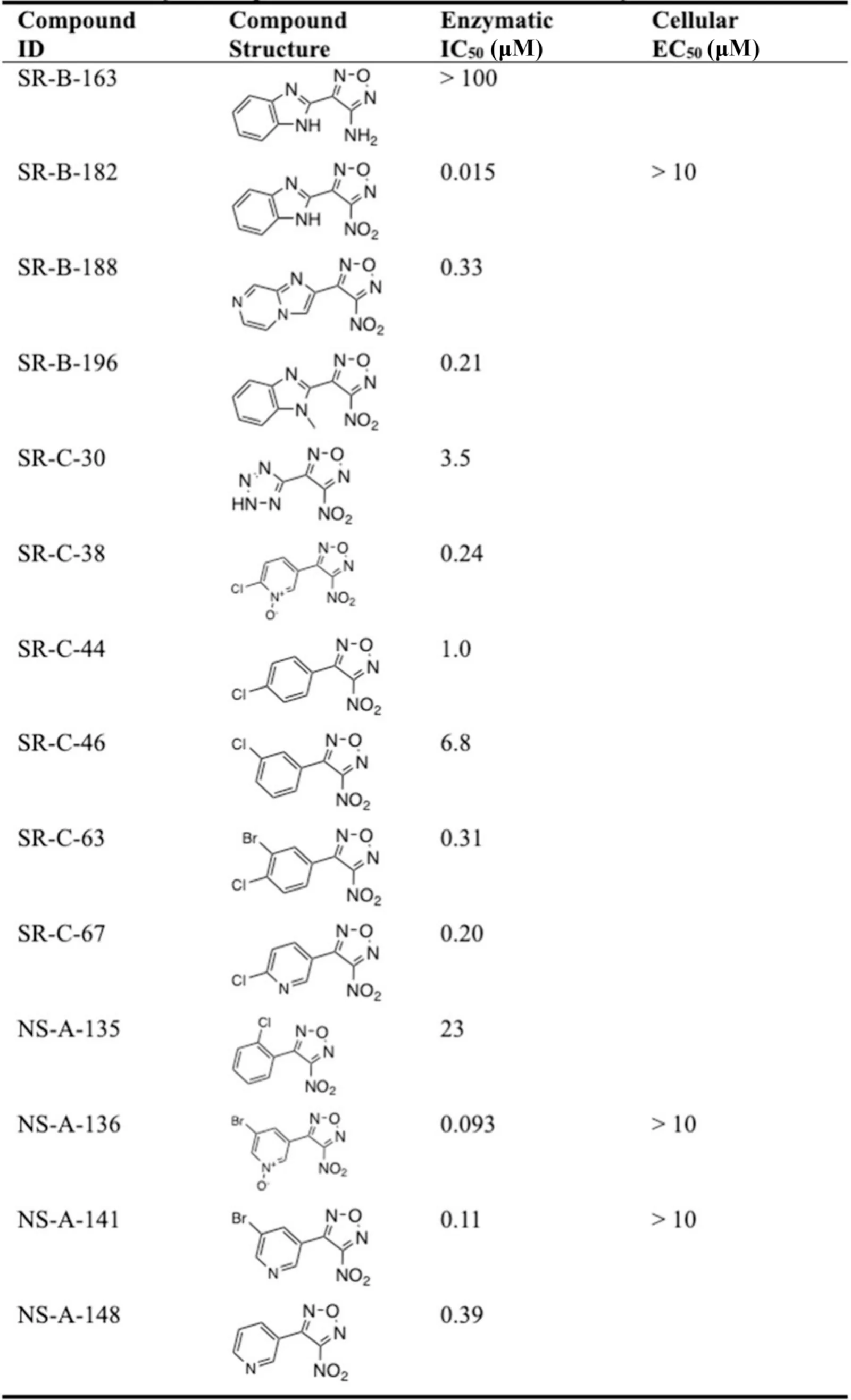
Newly Developed M^Pro^ Inhibitors and Their Potency

Transient expression of M^Pro^ in HEK293T cells induces acute toxicity, which can be counteracted by cell-permeable inhibitors that rescue host cells. Leveraging this phenotype, we previously developed a live-cell assay to quantify intracellular M^Pro^ inhibitors.^18^ In this assay, HEK293T cells are transiently transfected to express M^Pro^-eGFP and incubated with test inhibitors. Potent inhibitors suppress M^Pro^-mediated cytotoxicity, thereby increasing cell survival and overall eGFP expression levels. The eGFP fluorescence gain is then quantified to determine cellular EC_50_ values. Three potent inhibitors, SR-B-182, NS-A-136, and NS-A-141 with IC_50_ ≤ 110 nM were subjected to cellular potency evaluations using this assay. However, they all exhibit cellular EC_50_ values above 10 μM. This disparity suggests limited cellular permeability and/or rapid intracellular metabolism. Similarly, all these compounds were ineffective at inhibiting SARS-CoV-2 growth in Vero-Ace2-TMPRSS2 cells (antiviral EC_50_ > 3 μM), which is consistent with their weak cellular M^Pro^ inhibition.

To gain a deeper insights regarding this phenomenon, computational ADMET modeling was performed using ADMETlab 3.0. The SMILES structures of SR-B-182, NS-A-136, and NS-A-141 were first generated in ChemDraw 23.1.2 and then submitted to the online platform. From there, an individual report for each compound was produced, characterizing all computationally predicted ADMET profile features. All three compounds exhibited acceptable predicted Caco-2 permeability values, with log-unit scores above −5.15, suggesting a potential ability to cross the small intestinal epithelial barrier. In contrast, the predicted MDCK and PAMPA permeability values were unfavorable for all three compounds, indicating limited passive diffusion across cellular phospholipid bilayers. ADMETlab 3.0 further predicted a high probability that these compounds are substrates of CYP1A2, with confidence values exceeding 90%, while also suggesting moderate-to-high likelihood of metabolism by other cytochrome P450 machinery, including CYP2C9 and CYP3A4 (Figure 4). These predictions may help explain the observed discrepancy between enzymatic and cellular activities. Nevertheless, radar-plot analysis of drug-likeness metrics indicated that all three compounds largely fell within the optimal range for therapeutic candidate development. Therefore, further optimization to enhance cellular potency will be necessary before advancing molecules in this new chemotype group to antiviral testing. The primary focus of this study was the discovery and structural characterization of novel covalent chemotypes identified through high-throughput screening. Notably, the optimized analog SR-B-182 exhibits an IC_5_0 of 15 nM, placing it among the most potent M^Pro^ inhibitors reported to date, while displaying a distinct S_N_Ar-based covalent mechanism and binding mode. Detailed comparative analyses of selectivity, binding thermodynamics, and resistance profiles are beyond the scope of the present work and represent important directions for future studies.

**Figure 4:**
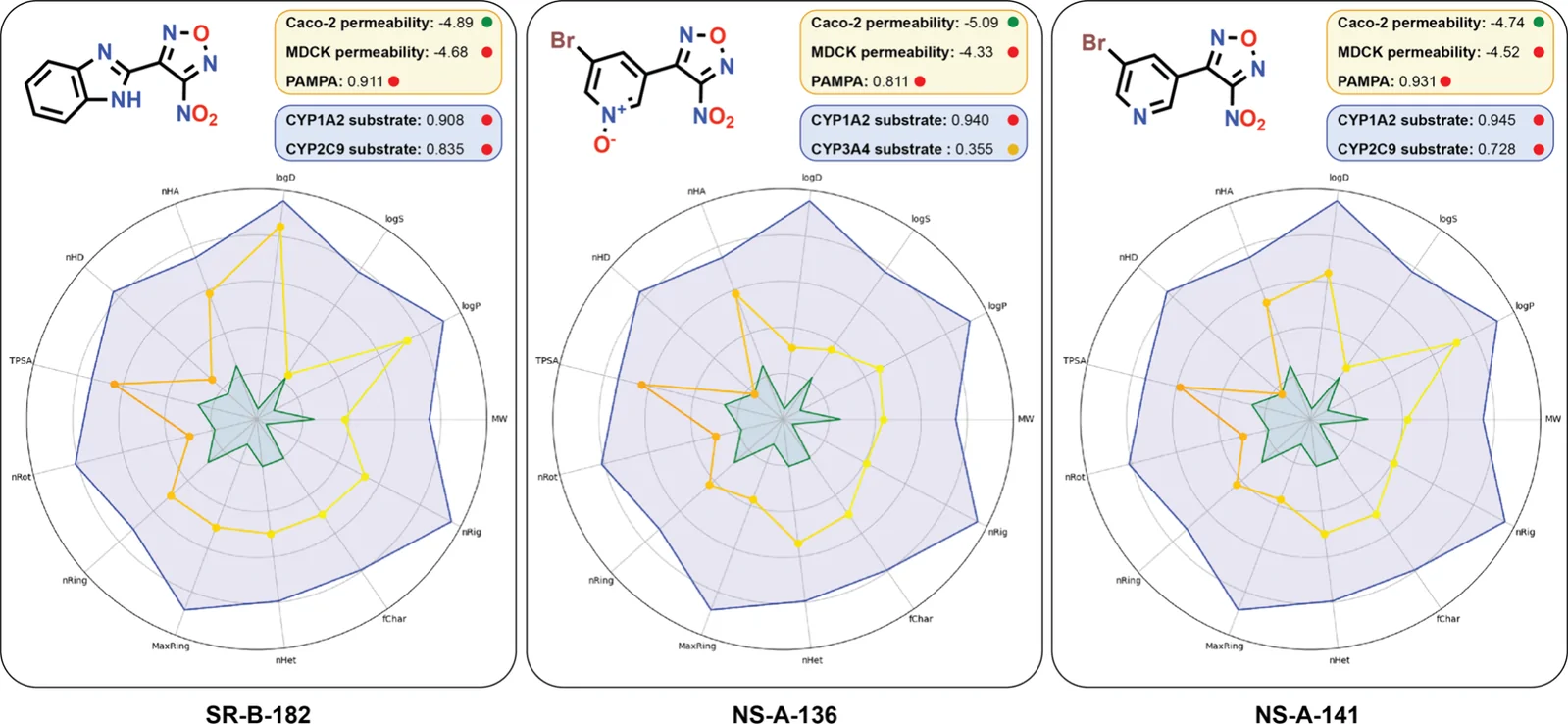
Computational ADMET profiles of SR-B-182, NS-A-136, and NS-A-141 predicted using ADMETlab 3.0. All three compounds were predicted to exhibit acceptable Caco-2 permeability, suggesting potential capacity to cross the small intestinal epithelial barrier. In contrast, MDCK and PAMPA permeability predictions indicated unfavorable passive membrane transport properties. The compounds were also predicted to undergo metabolism by multiple cytochrome P450 enzymes, with CYP1A2 substrate probability exceeding 90%. Radar plots summarizing drug-likeness descriptors (MW: molecular weight; logP: octanol–water partition coefficient; logS: aqueous solubility; logD: distribution coefficient; nHA: number of hydrogen-bond acceptors; nHD: number of hydrogen-bond donors; TPSA: topological polar surface area; nRot: number of rotatable bonds; nRing: number of rings; MaxRing: maximum ring size; nHet: number of heteroatoms; fChar: formal charge; and nRig: number of rigid bonds) showed that the predicted values for these compounds generally fell within the acceptable range (yellow line), bounded by the upper (blue) and lower (green) reference limits, and therefore were still potential for future optimization.

In summary, using Sub4, a fluorogenic M^Pro^ substrate with simple and scalable synthesis, we conducted high-throughput screening of two diversity libraries containing ∼ 75,000 compounds and identified 40 hits with IC_50_ values below 30 μM. Four hits were confirmed with freshly purchased samples and characterized by X-ray protein crystallography. In the determined structures, two inhibitors formed covalent adducts with Cys145, and in both complexes, His41 reoriented to enable π-π stacking interactions with the binding ligand. Mechanistically, CDI5573 undergoes an S_N_Ar reaction to generate a covalent adduct with Cys145, whereas CDI8009 proceeds via nucleophilic acyl substitution to yield a thioester with Cys145. Guided by the CDI5573 complex, a SAR campaign was conducted, leading to the development of multiple potent inhibitors. One inhibitor exhibits an IC_50_ of 15 nM, placing it among the most potent M^Pro^ inhibitors reported to date. Further optimization of this new chemotype of M^Pro^ inhibitors will be required to advance candidate molecules into antiviral testing.

## Supplementary Material







## Notes

The expression and crystallization of M^Pro^; the structural determination of M^Pro^ bound with all inhibitors; the synthesis and characterization of all newly developed inhibitors; the determination of enzymatic IC_50_, and cellular EC_50_ to inhibit ectopically expressed M^Pro^ in 293T cells; Tables S1–S2 show all parameters for the X-ray crystallography analysis of M^Pro^ bound with CDI5573 and CDI8009; Finally determined structures have been deposited into the Protein Data Bank with the entry codes as 9BTE (M^Pro^-CDI5573) and 9BST (M^Pro^-CDI8009MPI97) (PDF)
24 final hits with their activity data and chemical information (XLSX)
32 final hits with their activity data and chemical information (XLSX)

## Abbreviations

COVID-19: coronavirus disease 2019;
SARS-CoV-2: severe acute respiratory syndrome coronavirus 2;
M^Pro^: main protease;
NMV: nirmatrelvir.
