# Supplementary material for: High-Throughput Screening Identifies Covalent Inhibitors with Unique Chemistry and Binding Modes for SARS-CoV‑2 Main Protease
Source: JACS Au. 2026 Jul 13;6(9):4888–96. doi: 10.1021/jacsau.6c00371 (PMC13625513; doi:10.1021/jacsau.6c00371)

Supplementary Information

For

High-Throughput Screening Identifies Covalent Inhibitors with Unique Chemistry and Binding Modes for SARS-CoV-2 Main Protease

Sandeep Atla[a,‡], Kai S. Yang[a,‡], Erol C. Vatansever [a,‡], Eun Jeong Cho[b,c], Xinyu Ma[a], Yugendar R. Alugubelli[a], Satyanarayana Nyalata[a], Lauren R. Blankenship[a], Kaustav Khatua[a], Demonta Coleman[a], Xuejiao Guo[a], Rachel M. Sammons[b,c], Dorsa Rabie[a], Banumathi Sankaran[d], Tahmina Pervin[i], Benjamin W. Neuman[i], Chia-Chuan D. Cho[a], Lai Hoang Son Le[a], Alan Pham[a], Nagarjun R. Mallampudi[a], Kevin N. Dalby[b,c], Shiqing Xu[a,e,*], and Wenshe Ray Liu[a,e,f,g,h,*]

[a]Texas A&M Drug Discovery Center and Department of Chemistry, College of Arts and Sciences, Texas A&M University, College Station, TX 77843, United States

[b]Targeted Therapeutic Drug Discovery & Development Program, the University of Texas, Austin, TX 78712, United States

[c]Division of Chemical Biology & Medicinal Chemistry, College of Pharmacy, the University of Texas, Austin, TX 78712, United States

[d]Molecular Biophysics and Integrated Bioimaging, Berkeley Center for Structural Biology, Laurence Berkeley National Laboratory, Berkeley, CA 94720, United States

[e]Department of Pharmaceutical Sciences, Irma Lerma Rangel College of Pharmacy, Texas A&M University, College Station, TX 77843, United States

[f]Institute of Biosciences and Technology and Department of Translational Medical Sciences, School of Medicine, Texas A&M University, Houston, TX 77030, United States

[g]Department of Biochemistry and Biophysics, College of Agriculture and Life Sciences, Texas A&M University, College Station, TX 77843, United States

[h]Department of Cell Biology and Genetics, School of Medicine, Texas A&M University, College Station, TX 77843, United States

[i]Department of Biology, College of Arts and Sciences, Texas A&M University, College Station, TX 77843, United States

[‡]Contributed equally to the paper.

Correspondence should be addressed to shiqing.xu@tamu.edu and wsliu2007@tamu.edu.

The PDF file includes:

Supplementary Materials and Methods

Figure S1-S36

Table S1-S2

Supplementary Synthesis Details

Synthesis Schemes S1-S6

LC-MS Data of Final Compounds

Supplementary Materials and Methods

Expression and Purification of SARS-CoV-2 M^{Pro}

The expression plasmid pET28a-His-SUMO-CoV-2 M^{Pro} was constructed in a previous study (*43*). We used this construct to transform *E. coli* BL21(DE3) cells. A single colony grown on an LB plate with 25 µg/mL kanamycin was picked and grown in 5 mL LB media supplemented with 25 µg/mL kanamycin overnight. We inoculated this overnight culture to 6 L 2YT media with 25 µg/mL kanamycin. Cells were grown to OD_{600} as 0.8. At this point, we added 1 mM IPTG to induce the expression of His-SUMO-CoV-2 M^{Pro}. Induced cells were let grown for 3 h and then harvested by centrifugation at 12,000 rpm, 4 °C for 30 min. We resuspended cell pellets in 150 mL lysis buffer (20 mM Tris-HCl, 100 mM NaCl, 10 mM imidazole, pH 8.0) and lysed the cells by sonication on ice. We clarified the lysate by centrifugation at 16,000 rpm, 4 °C for 30 min. We decanted the supernatant and mixed with Ni-NTA resins (GenScript). We loaded the resins to a column, washed the resins with 10 volumes of lysis buffer, and eluted the bound protein using elution buffer (20 mM Tris-HCl, 100 mM NaCl, 250 mM imidazole, pH 8.0). We exchanged buffer of the elute to another buffer (20 mM Tris-HCl, 100 mM NaCl, 10 mM imidazole, 1 mM DTT, pH 8.0) using a HiPrep 26/10 desalting column (Cytiva) and digested the elute using 10 units SUMO protease overnight at 4 °C. The digested elute was subjected to Ni-NTA resins in a column to remove His-tagged SUMO protease, His-tagged SUMO tag, and undigested His-SUMO-CoV-2 M^{Pro}. We loaded the flow-through onto a Q-Sepharose column and purified CoV-2 M^{Pro} using FPLC by running a linear gradient from 0 to 500 mM NaCl in a buffer (20 mM Tris-HCl, 1 mM DTT, pH 8.0). Fractions eluted from the Q-Sepharose column was concentrated and loaded onto a HiPrep 16/60 Sephacryl S-100 HR column and purified using a buffer containing 20 mM Tris-HCl, 100 mM NaCl, 1 mM DTT, and 1 mM EDTA at pH 7.8. The final purified protein was concentrated and stored in a -80 °C freezer.

Antiviral EC_{50} testing

Vero E6-ACE2-TMPRSS2 cells, a puromycin-selectable cell line expressing the human ACE2 and TMPRSS2 genes (BEI Resources, NR54970), cultured in a 37 °C incubator with 5% CO_2 in DMEM supplemented with 10% fetal bovine serum, 1× antibiotic/antimycotic and puromycin dihydrochloride (10 µg/ml final concentration), were used for virus growth, titration of infectivity, and antiviral testing. SARS-CoV-2 BQ.1.1 (local isolate; GISAID accession EPI_ISL_17371329) infectious titer was determined by tissue culture infectious dose 50% ($TCID_{50}$), calculated by the method of Reed & Muench. Cells were inoculated at a multiplicity of 0.02 $TCID_{50}$ units per cell, incubated for 1 h at 37 °C to allow for virus adsorption, rinsed three times with phosphate-buffered saline pH 7 to remove unbound inoculum, and treated by the addition of culture medium containing various concentrations of candidate antivirals. Aliquots of cell culture supernatant were collected at 24 h and 48 h after inoculation, and the amount of virus genome was measured by Luna One-Step RT-qPCR (NEB, Ipswich, MA, US) with the N1 primer set targeting the nucleoprotein gene of the virus, calibrated to a standard curve of inactivated viral RNA (BEI Resources, NR-52285).

Assay Evaluation and Optimization for Compound Screening

M^{Pro} activity was determined by RFU of free AMC released from Sub4. Inhibitors block this protease activity, resulting signal drop. The assay was evaluated for its dose dependent signal change against M^{Pro}, substrate, and a known inhibitor in a miniaturized format (10 ⌈L of assay volume). The intra-plate variation was validated.

Detailed Process:

1. Assay Protocol:
   Assay buffer: 20 mM Tris, pH 7.4, 10 mM NaCl, 0.5 mM EDTA, 1 mM DTT, 0.01% Triton X-100, 10 ⌠g/mL BSA.
   General procedure: 5 ⌠L of 2· enzyme was added to a 384-well black assay plate (Corning # 3821). Inhibitor was added to enzyme solution and incubated for 30 min at room temperature. After the addition of 5 ⌠L 2· substrate solution, the signal change was monitored kinetically (per min) on a Neo2 plate reader with ex/em at 370/450 nm.
2. Preliminary tests:
   2.1 Enzyme dose dependency: RFU increased as increasing M^{Pro} when 600 nM of Sub4 was used. Initial rate was calculated from the RFU change for the first 5 min of the assay. This dose dependence is presented in Figure S1.
   2.2 Substrate dose dependency: RFU increased as Sub4 dose increases when 100 nM of M^{Pro} was used. Initial rate was calculated from the RFU change for the first 30 min. Results are presented in Figure S2.
3. Assay validation with a known inhibitor: Enzyme activity decreased as increasing inhibitor dose, result calculated IC_{50} = 50.8 ± 3.18 nM, H = 2.39 ± 0.4 when 10 nM and 10 µM of M^{Pro} and substrate were used, respectively. Initial rate was calculated from the RFU change for the first 30 min
4. Intra plate validation was conducted by filling 2 columns with positive control (Sub4 only) and 2 columns with negative control (M^{Pro} + Sub4) manually using a multichannel pipettor. Initial rate for the first 30min was used to calculate assay statistics. z' factor of 0.75 confirmed the assay readiness for compound screening.

Compound Screening

Please see two screening reports after the ESI-MS characterization session.

Characterization of M^{Pro} Inhibition for Identified Hit Molecules and All Synthesized Inhibitors

For retesting of identified hit molecules and newly synthesized inhibitors, we conducted the assay using 20 nM M^{Pro} and 10 ⌠M Sub3 (DABCYL-Lys-Thr- Ser-Ala-Val-Leu-Gln-Ser-Gly-Phe-Arg-Lys-Met-Glu-EDANS). We dissolved all inhibitors in DMSO as 10 mM stock solutions. Sub3 was dissolved in DMSO as a 1 mM stock solution and diluted 100 times in the final assay buffer containing 10 mM sodium phosphate, 10 mM NaCl, 0.5 mM EDTA, and 1.25% DMSO at pH 7.6. We incubated M^{Pro} and an inhibitor in the final assay buffer for 30 min before adding the substrate to initiate the reaction catalyzed by M^{Pro}. The production format was monitored in a fluorescence plate reader with excitation at 336 nm and emission at 490 nm. We calculated the initial rate according to the fluorescent intensity in the first 5 min by linear regression, which was then normalized according to the initial rate of positive and negative controls.

X-Ray Crystallography Analysis of M^{Pro}-Inhibitor Complexes

The production of crystals of M^{pro}-inhibitor complexes was following the previous protocols (13). Data were collected on a Bruker Photon II detector or the Advanced Light Source (ALS) beamline 5.0.2 using a Pilatus3 S 6M detector. The diffraction data were indexed, integrated and scaled with iMosflm or PROTEUM3. All the structures were determined by molecular replacement using the structure model of the free enzyme of the SARS-CoV-2 M^{pro} [Protein Data Bank (PDB) ID code 7JPY] as the search model using Phaser in the Phenix package. *JLigand* and *Sketcher* from the CCP4 suite were employed for the

generation of PDB and geometric restraints for the inhibitors. The inhibitors were built into the Fo-Fc density by using *Coot*. Refinement of all the structures was performed with Real-space Refinement in Phenix. Details of data quality and structure refinement are summarized in Tables S1-S2.

Electrospray Ionization Mass Spectrometry (ESI-MS) Analysis of M^{Pro} Bound with CDI5573

Purified M^{Pro} protein (10 μM) was incubated with CDI5573 (50 μM; 5-fold molar excess) in [10 mM sodium phosphate, 10 mM sodium chloride, and 0.5 mM EDTA, pH 7.6] for 30 mins at 37°C prior to analysis. Samples were analyzed by an Orbitrap Exploris™ 480 mass spectrometer (Thermo Fisher Scientific, Inc., Waltham, MA) coupled with an EASY-nLC 1000 system (Thermo Fisher Scientific, Inc., Waltham, MA). Protein–inhibitor sample were separated by a C4 column packed with butyl-bonded silica, using solvents A (0.1% formic acid in 2% acetonitrile, 98% water) and B (0.1% formic acid in 100% acetonitrile), with a continuous gradient from 2 to 95% B over 25 min.

High-resolution Orbitrap mass spectrometry revealed a clear mass shift upon incubation of the protein with inhibitor. The deconvoluted spectrum of the apo protein displayed a dominant species at 33,855 Da, consistent with the calculated molecular weight of the unmodified protein. Upon incubation with CDI5573, a dominant species was observed at 34,056 Da. Results are presented in Figure S4.

Compound Screening Report 1

Abstract: Assay optimization/automation, Compound screening, and Hit identification/validation

Summary: TTP tested new batch of M^{Pro} enzyme/Sub4 and evaluated the assay tolerance against assay additives. Automated assay protocol was further validated, followed by a small scale of pilot screening. Primary screening of ~30,000 compound at a single dose was conducted, hits were identified/validated in multiple experiments and IC_{50} of 24 compounds were examined.

Detailed progress:

1. General assay protocol:

    1.1. Reagents: [M^{Pro}] = 20 nM, [Sub4] = 10 μM, assay buffer (20 mM Tris, pH7.4, 10 mM NaCl, 0.5 mM EDTA, 1 mM DTT, 0.02% Triton X-100 and 10 μg/mL BSA)

    1.2. Assay volume = 10 mL, monitored RFU with ex/em at 360/450 nm kinetically (for assay validation/hit validation) or after 2h incubation (for primary screening).

2. Assay validation:

    2.1. Evaluation of new batches of enzyme and Sub4: New batches of M^{Pro}/Sub4 were received, and their consistent activities (or little better) was confirmed by testing old and new batches of M^{Pro}/Sub4 in parallel. As shown in Figure S5, the combination of new batches of M^{Pro} and Sub4 showed a bit higher initial rate (RFU change during the first 30 min of assay) than other combinations, confirming the reagents are suitable for screening.

    2.2. Evaluation of assay tolerance against additives (Figure S6):

DMSO: the assay was stable up to 1.56%.
BSA: tolerant up to 0.56%. The M^{Pro} activity was slowed down at higher BSA.
TX-100: The RFU signals were generally low in addition to high deviations, suspecting either (or both) M^{Pro} or/and Sub4 are sticky to plastics. The data is not reliable due to the high std.

2.3. Incubation time for end-point reading was determined.

A half of 384 well plate was filled with a positive (Sub4 only) sample and a half of the plate was filled with a negative (M^{Pro} + Sub4) sample using a bulk dispenser. Both samples contain 0.1 % DMSO to mimic screening compounds and the RFUs at different incubation times were monitored. As shown in Figure S7, RFU from positive sample was consistent while the RFU from negative sample increase as increasing incubation time, resulting increase of S/B concurrently. The z' was also increased slightly as increasing incubation time. Z' is a statistical parameter to judge whether the response in a particular assay is large enough to warrant further attention *(Zhang JH, Chung TDY, Oldenburg KR (1999). "A simple statistical parameter for use in evaluation and validation of high throughput screening assays". J. Biomol. Screening. 4: 67–73)*. In order to achieve strong S/B (signal-to-background ratio) for hit identification, 2h incubation time resulting S/B of ~10 was selected for end-point reading. Additionally, the >0.8 of z' confirmed the assay robustness.

3. Primary screening

3.1. Summary of screening procedure: 29,669 compounds from Chemdiv diversity, Maybridge, NCI diversity sets were plated into 94 assay plates and screened at 12.5 µM at a single dose for 7 days. Each plate has total 64 wells reserved for both positive and negative controls. General plate map and screening flow are as illustrated in Figure S8. Each assay plate was read twice for intrinsic RFU of compounds (compounds and M^{Pro}) and assay readout after 2h incubation. Compounds were incubated with M^{Pro} for 30min at rt before the assay initiation. Inhibition % of each compound was calculated based on the controls (inh % = 100 ($RFU_{Negative}$-RFU_{sample})/($RFU_{Negative}$-$RFU_{Positive}$)).

3.2. Result of primary screening: The z's from each assay plates were consistent (Figure S9). The averaged assay statistics of z' = 0.88 ± 0.02 and S/B = 6.85 ± 0.53 confirmed the assay robustness and data reliability. Assay data was uploaded to CDD (Collaborative Drug Discovery). Total 49 compounds showed >25% inhibition but most of them showed < 50% (Figure S9). Including additional 28 compounds which showed higher intrinsic fluorescence, total 77 compounds (31 compounds from Maybridge, 36 compounds from Chemdiv, and 10 compounds from NCI) were selected for further validation.

4. Hit validation.

4.1. Cherry pick screening at a single dose of 10 µM, duplicates: 4 different assays (Rescreening to validate reproducible inhibition activity, counter screens to validate interferences with assay component (Sub4 only, 10 µM Sub4 with compound) or assay product (AMC only, 600 nM of AMC with compounds), and rescreening in the presence of CAT/SOD to examine ROS (reactive oxygen species)-driven inhibition, [CAT] = 1U/µL and [SOD] = 0.3U/µL) were conducted in parallel. Assays were monitored kinetically for 3h. Kinetic assay profiles of each compounds were investigated to sort out false hits due to assay interferences. All 28

compounds with high intrinsic fluorescence were found to interfere with either AMC or Sub4, so they were excluded from further study. Additional 25 compounds which failed to reproduce inhibition activity or interfered either Sub4 or AMC were also excluded.

4.2. Dose response experiment: Remaining **24** compounds were further validated in varying doses of compounds (0-100 µM, 3x dilution) in duplicates. RFU changes were monitored for 3h and the rate at 2h was used for IC_{50} calculation. IC_{50} calculation was calculated using a standard Hill equation, or the four-parameter logistic curve embedded in CDD program. *Details on compounds information and data are provided in a separate excel file.* Briefly, 13 compounds showed IC_{50} between 0.72 – 10 µM, 6 compounds showed IC_{50} between 10 - 30 µM, 5 compounds showed IC_{50} over 100 µM. Among them 15 compounds lost inhibition completely in the presence of CAT/SOD, indicating their inhibition was ROS driven. One compound (Chemdiv Diverse 0439-0102) which showed most potent activity lost inhibition activity ~ 50% in the presence of CAT/SOD. ROS are known to irreversibly oxidize solvent-accessible amino acid residues on the target protein, including the catalytic cysteine residue of cysteine proteases. Furthermore, production of ROS by redox cycling compounds can rapidly consume DTT. Therefore, ROS-based inhibition of Cys-residue is normally investigated in the presence of CAT (catalase, converts H_2O_2 to oxygen) and SOD (superoxide dismutase, catalyzes dismutation of O_2^- to produce H_2O_2) in early stage hit validation.

5. Appendix: Excel file 1 listed 24 final hits with their activity data and chemical information

   Descriptions for column headings are as below:

   - ✓ Cpds RFU: compound's intrinsic RFU. RFU <500 is normally considered as no fluorescence.
   - ✓ P_inh%: inhibition % in primary screening tested at 12.5 µM
   - ✓ C_inh%: inhibition % in cherry pick screening tested at 10 µM
   - ✓ CAT/SOD_inh%: inhibition % in cherry pick screening tested at 10 µM in the presence of CAT/SOD. Reduced number compared with C_inh% represents ROS-driven inhibition mechanism.
   - ✓ AMC_% stability: compound's interference with AMC. 100% indicates no interference.
   - ✓ Sub only_% stability: compound's interference with AMC-labeled Sub4. 100% indicates no interference.

Compound Screening Report 2

Abstract: 2nd Compound screening of extended TTP deck and Hit identification/validation

Summary: TTP validated new batch of M^{Pro} enzyme/Sub4 and screened rest of TTP compounds (~45,000 compounds) collection using the optimized M^{Pro} assay at a single dose like the first screening. Primary hits were validated by rescreen and counter screens. Potential of ROS was also investigated in the presence of CAT/SOD. The potency (IC_{50}) of 32 compounds was evaluated.

Detailed progress:

1. General assay protocol (consistent with the 1st primary screening):

1.1. Reagents: [M^{Pro}] = 20 nM, [Sub4] = 10 µM, assay buffer (20 mM Tris, pH7.4, 10 mM NaCl, 0.5 mM EDTA, 1 mM DTT, 0.02% Triton X-100 and 10 µg/mL BSA)

1.2. Assay volume = 10 µL, monitored RFU with ex/em at 360/450 nm at 0 and 2h incubation (for primary screening) and kinetically (for hit validation).

2. Primary screening:

2.1. Summary of screening procedure: 45,169 compounds from Br-containing diversity and fragment (Chemdiv), Kinase focused (Chembridge), and Fsp3 enriched and natural like (Life Chemical) sets were plated into 144 assay plates and screened at 12.5 µM at a single dose for 10 days. Consistent with previous screening, each assay plate was read twice for intrinsic RFU of compounds (compounds and M^{Pro}, at 0 h incubation) and assay readout after 2h incubation. Compounds were incubated with M^{Pro} for 30min at rt before the assay initiation by addition of Sub4.

2.2. Result: The averaged assay statistics of z' = 0.86 ± 0.03 and S/B = 7.84 ± 0.49 confirmed the assay robustness and data reliability. Inhibition % of each compound was calculated based on the controls (inh % = 100x($RFU_{Negative}$-RFU_{sample})/($RFU_{Negative}$-$RFU_{Positive}$)). Negative control contains M^{Pro} and Sub4 while positive control contains Sub4 only. Assay data was uploaded to CDD (Collaborative Drug Discovery). Total 56 compounds showed >15% inhibition were selected for further validation. Any compounds exhibiting significant intrinsic fluorescence (>3x positive control) were not considered for hit selection.

3. Hit validation. (Details of hit selection flow is illustrated in Figure S10)

3.1. Rescreening and counter screening: All validation screens were conducted at two doses (10 and 50 µM) with duplicates. Counter screens include assays with 1) Sub4 only (10 µM AMC-labeled peptide with compound) and AMC only (assay product, 600 nM of AMC with compounds). Rescreening was also conducted in the presence of CAT/SOD to examine ROS (reactive oxygen species)-driven inhibition. Assays were monitored kinetically for 3h. Kinetic assay profiles of each compound were investigated to sort out false hits due to assay interferences or incomplete inhibition. Initial rate was calculated based on RFU changed between 0-2h and used for inhibition analysis. First, compounds failed to show reproducible activity or dose dependent inhibition were excluded. Second, compounds showing non-linear time-dependent kinetic profile were excluded. Remaining 39 compounds did not show significant sensitivity in both counter screens.

3.2. CAT/SOD effect: Total 32 compounds presenting >40 % inhibition at 50 µM were further examined for their sensitivity against CAT/SOD at two dosed (10 and 50 µM). While most compounds were not sensitive to CAT/SOD, two compounds, C066-4043 and E679-0499 lost their activity completely in the presence of CAT/SOD.

3.3. Dose response experiment: Total 32 compounds were further validated in varying doses of compounds (0-100 µM, 3x dilution) in duplicates. RFU changes were monitored for 3h and the

rate at 2h was used for IC_{50} calculation. IC_{50} was calculated using a standard Hill equation, or the four-parameter logistic curve embedded in CDD program. *Details on compounds information and data are provided in a separate excel file (details of column heading are summarized in Section 4, Appendix).*

4. Supplementary Excel file 2 Listed 32 final hits with their activity data and chemical information

   Descriptions for column headings are as below:

- ✓ Cpds RFU (10uM): compound's intrinsic RFU at 12.5 µM. RFU <500 is normally considered as no fluorescence.
- ✓ P_inh% (10uM): inhibition % in primary screening tested at 12.5 µM
- ✓ C_inh% (10uM/50uM): inhibition % in cherry pick screening tested at 10/50 µM
- ✓ CAT/SOD_inh% (10uM/50uM): inhibition % in cherry pick screening tested at 10/50 µM in the presence of CAT/SOD. Reduced number compared with C_inh% represents ROS-driven inhibition mechanism.

Supplementary Figures

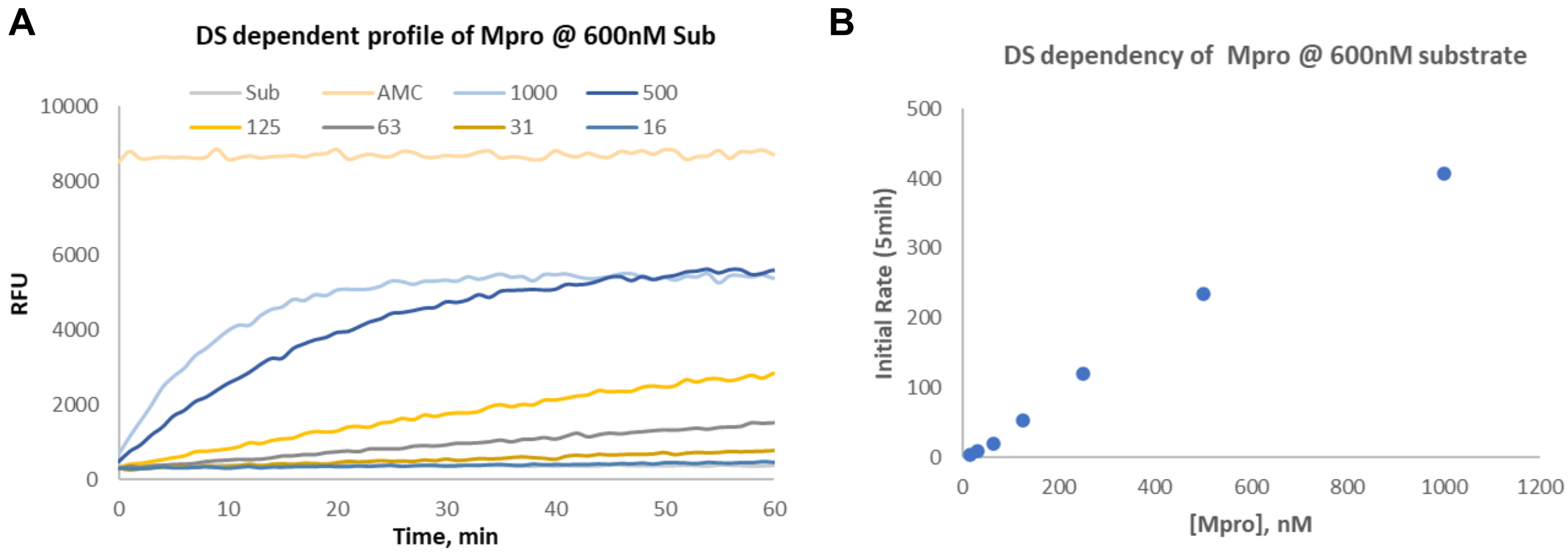

Figure S1. (**A**) M^{Pro}-catalyzed AMC release from Sub4. 600 nM Sub4 alone and 1 μM AMC were used as two controls. M^{Pro} concentrations are indicated as labels for different curves (16, 31, 63, 125, 500, and 1000 nM). (**B**) Dose dependence of M^{Pro}-catalyzed AMC release on the M^{Pro} concentration. The M^{Pro}-catalyzed AMC release rates were calculated based on the first 5-min RFU change divided by the elapse time.

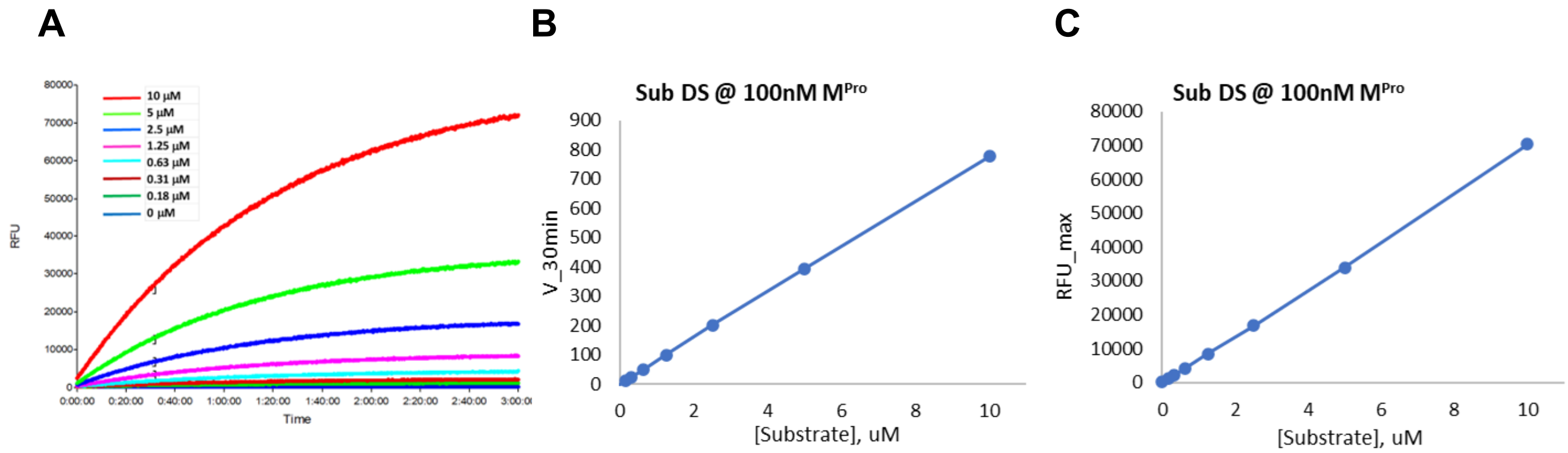

Figure S2. (**A**) 100 nM M^{Pro}-catalyzed AMC release from different concentrations of Sub4 (0.31, 0.63, 1.25, 2.5, 5, and 10 μM). (**B**) Substrate concentration dependence of AMC release rate. The reaction rates were calculated based on the first 30-min RFU change shown in **A** divided by the elapse time. (**C**) Substrate concentration dependence of total AMC released after 3 h.

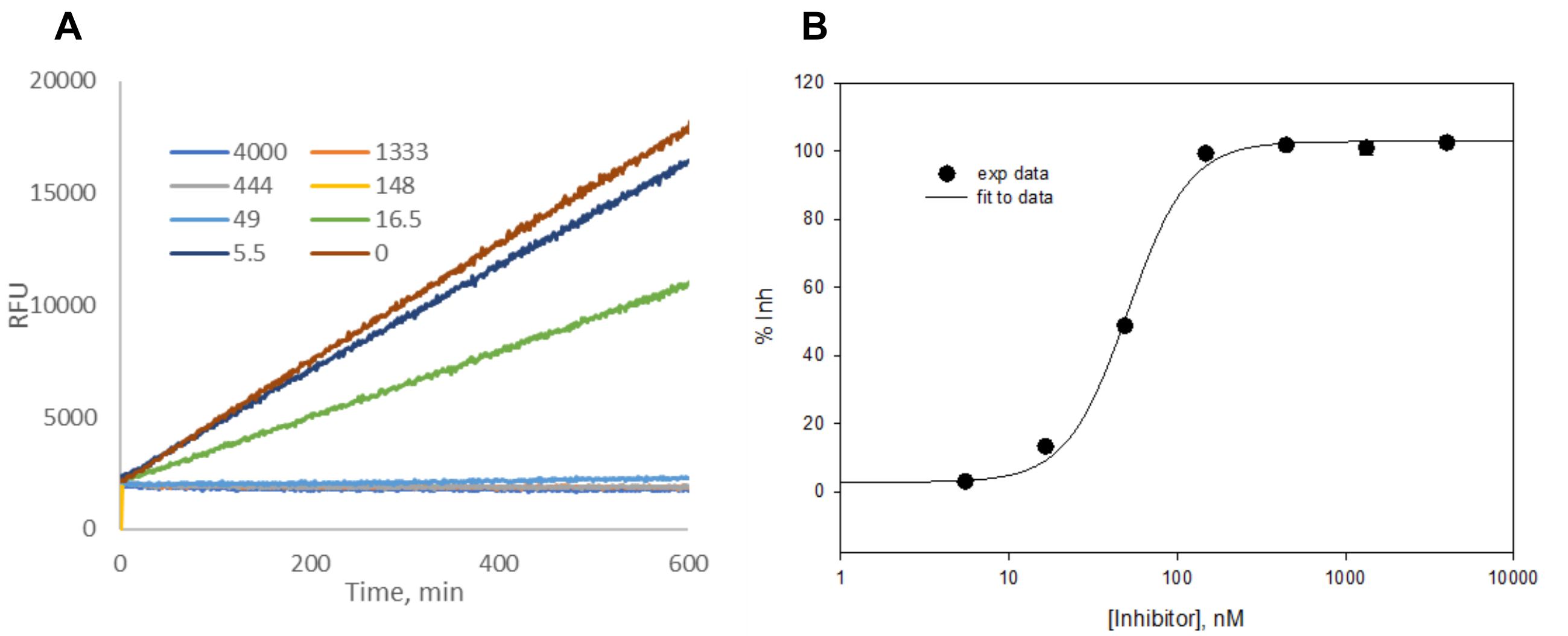

Figure S3. Dose dependent profile of M^{Pro}-catalyzed AMC fluorescence release from Sub4 in the presence of MPI3. (**A**) Raw fluorescence release data. Conditions: 20 nM M^{Pro}, 10 ⌠M Sub4, 0, 5.5, 16.5, 49, 148, 444, 1333, 4000 nM MPI3. (**B**) Initial rate vs MPI3 concentration.

A

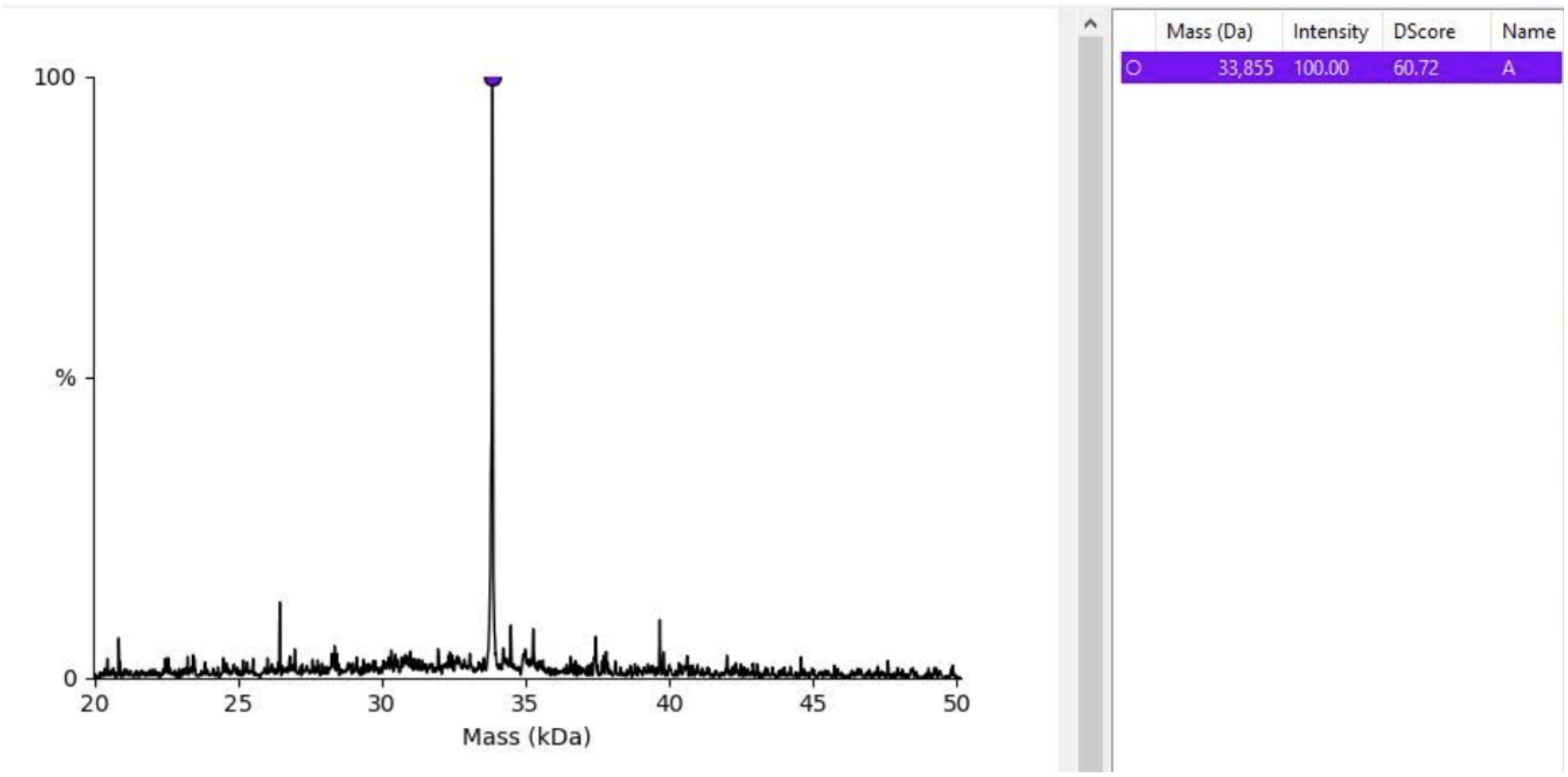

B

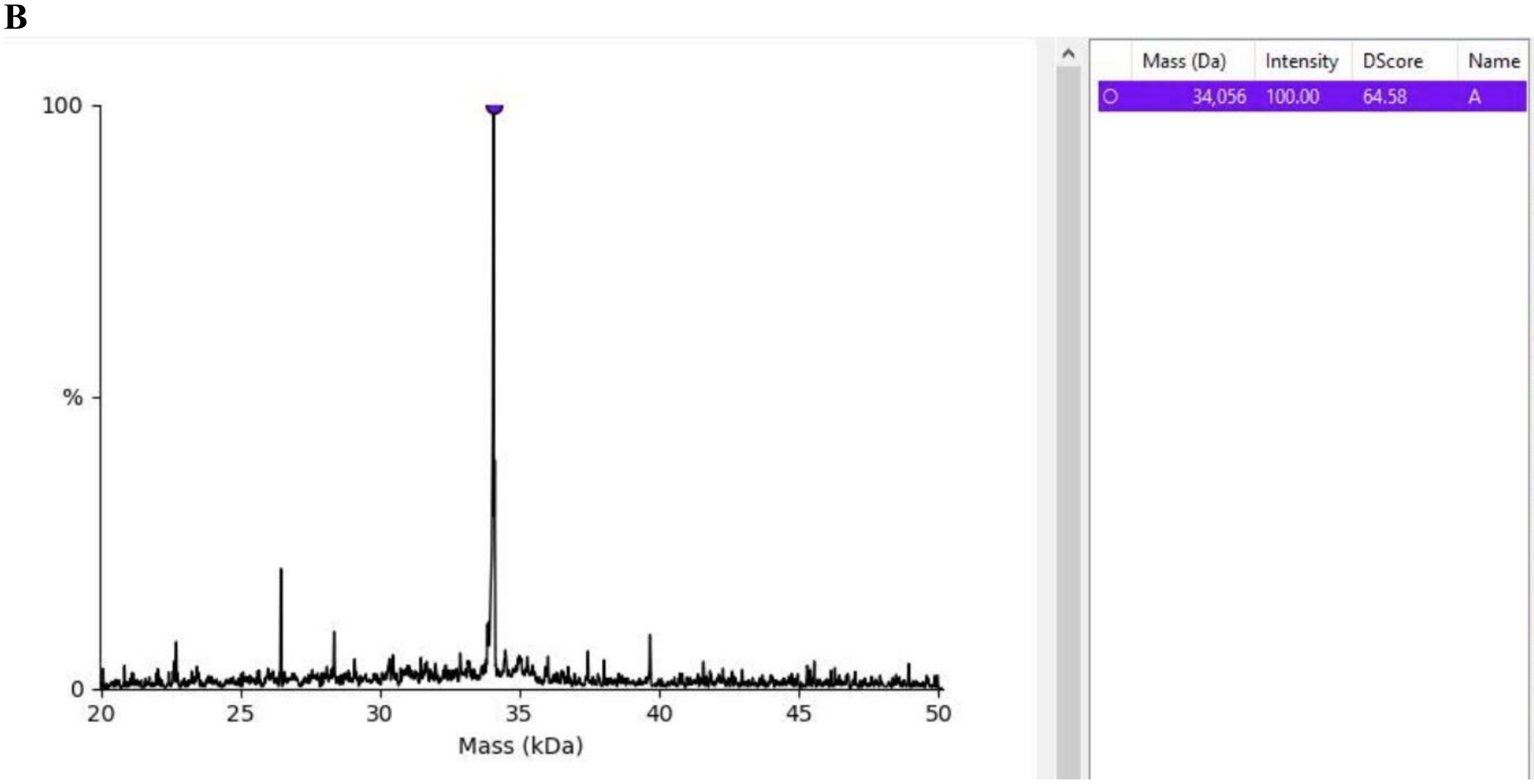

Figure S4. Deconvoluted ESI-MS spectra of (**A**) free M^{Pro} and (**B**) M^{Pro} bound with CDI-5573. Determined molecular weights for the two protein samples are 33,855 and 34,056 Da, respectively.

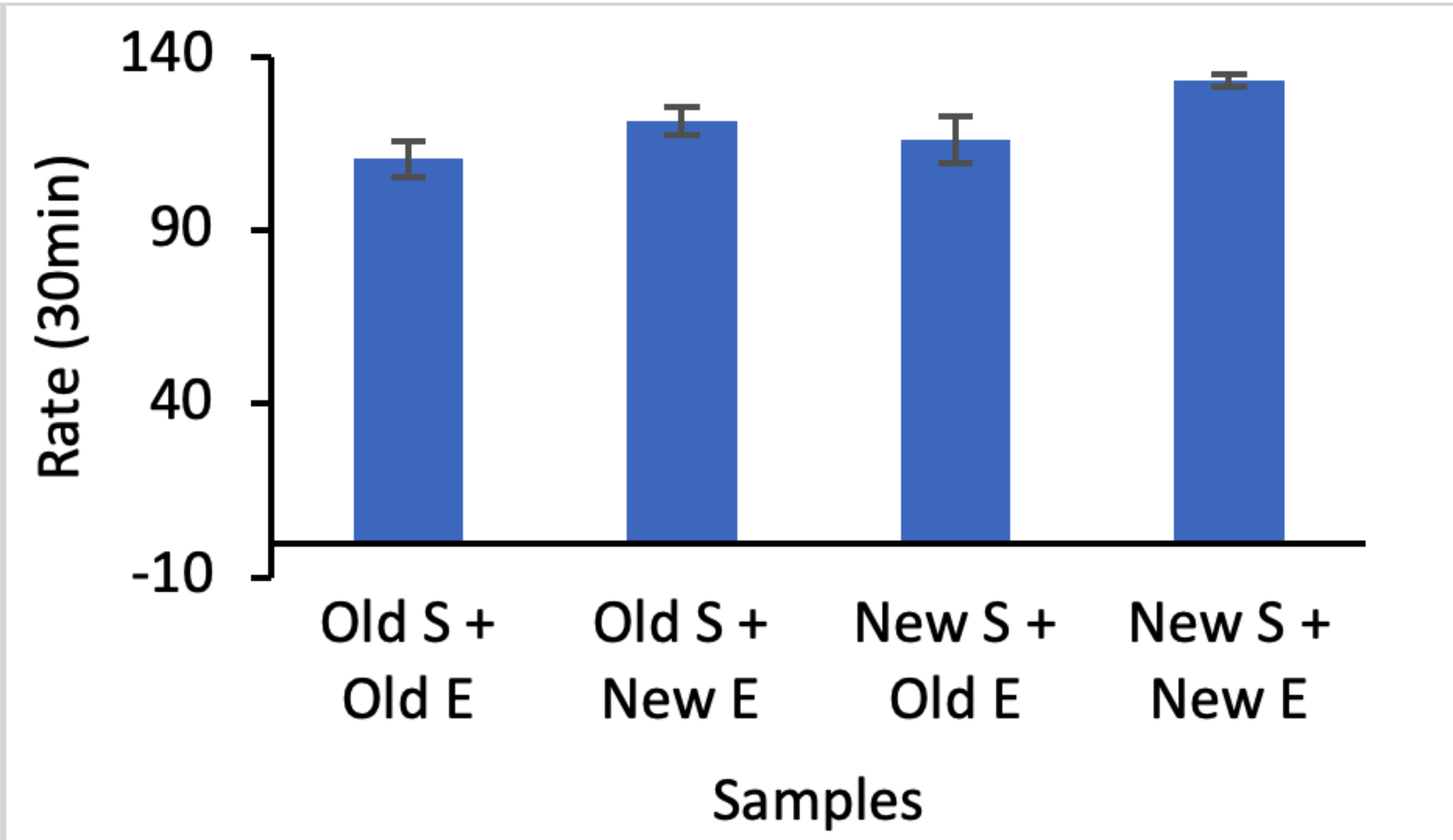

Figure S5. Comparison of old and new batches of M^{Pro} (E) and Sub4 (S).

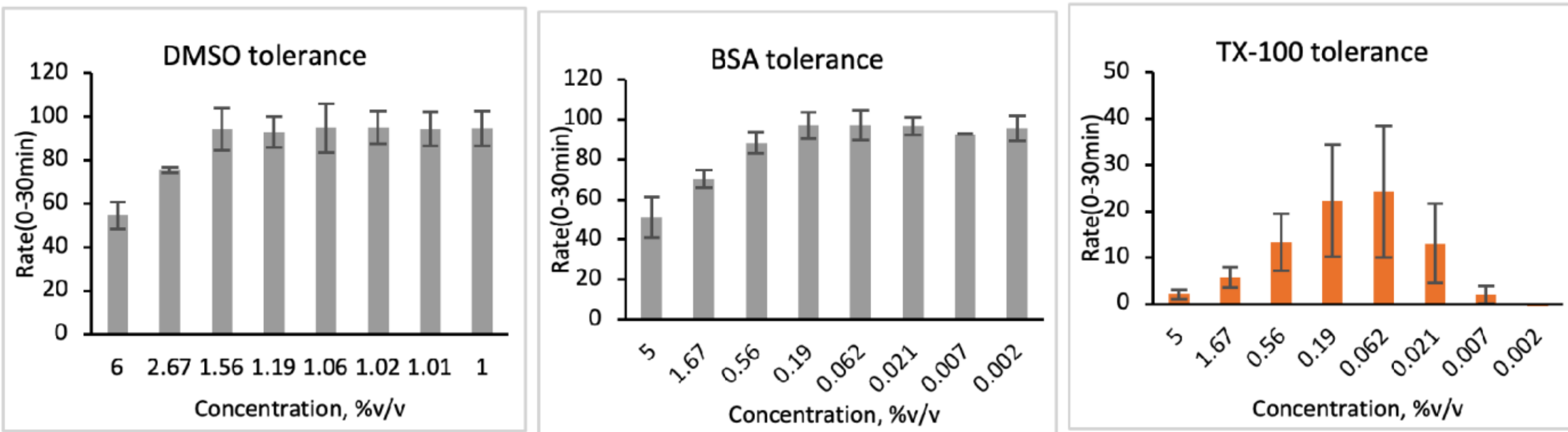

Figure S6. Assay tolerance against DMSO, BSA, and Detergent.

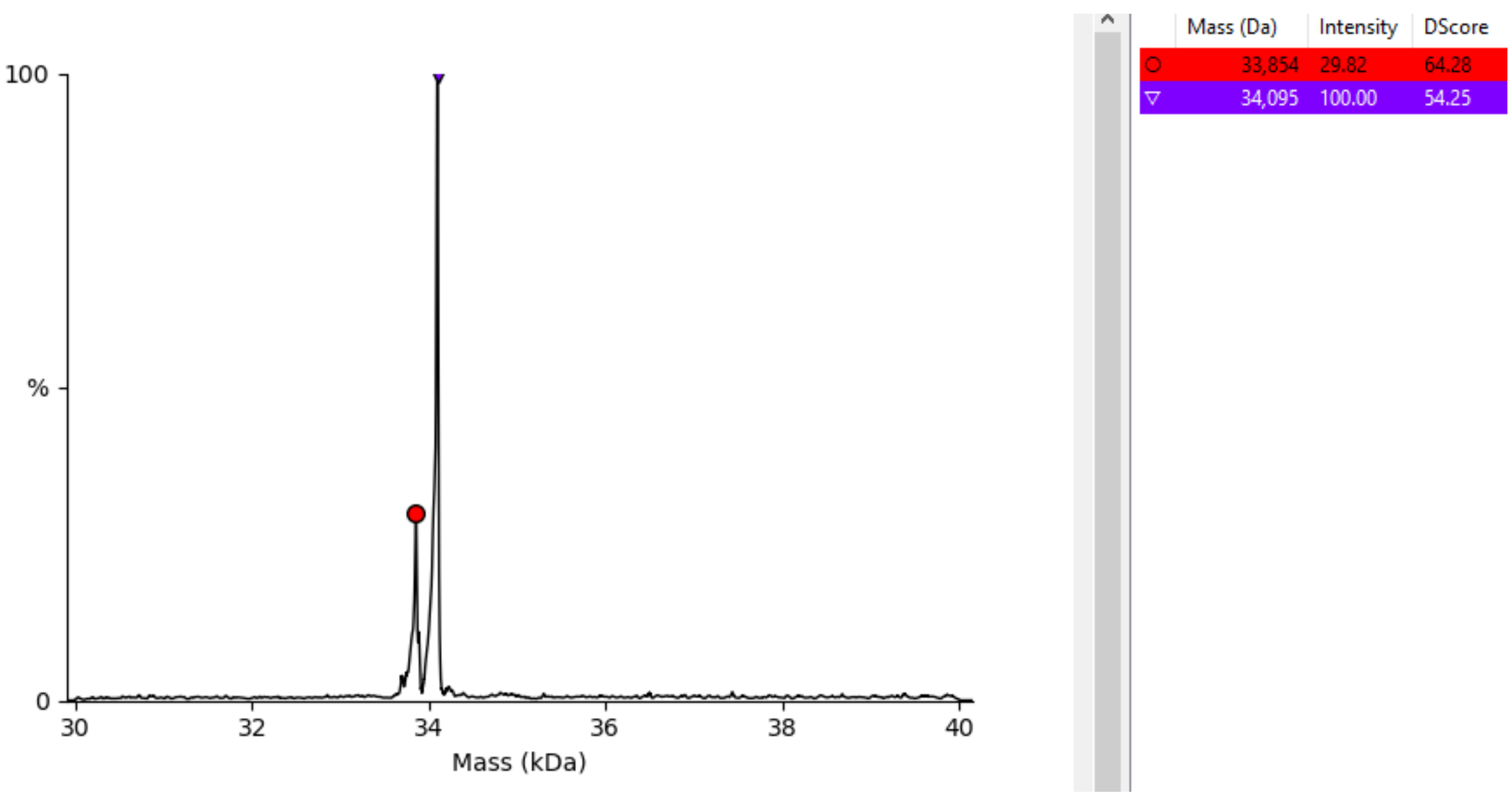

Figure S7. Mass spectrometry data of compound NS-A-136 reacting with M^{Pro}. The added mass of 241 daltons to the mass correlates to the binding of the compound to the M^{Pro} and loss of NO_2 from NS-A-136.

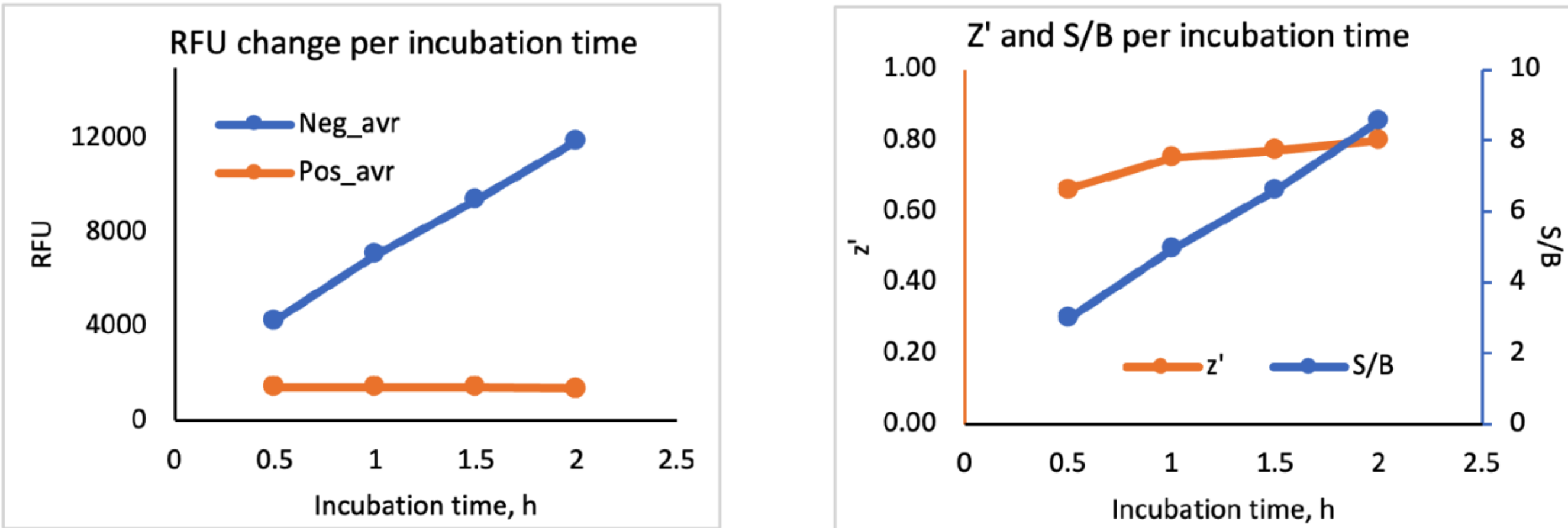

Figure S8. Incubation time optimization.

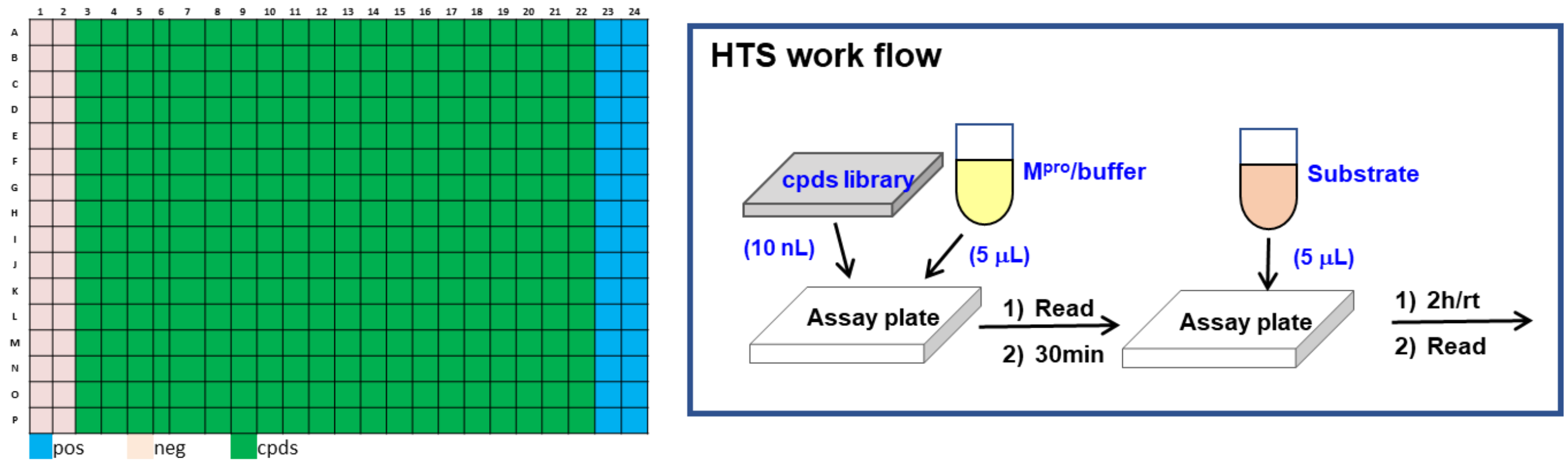

Figure S9. Illustration of assay plate map and HTS workflow.

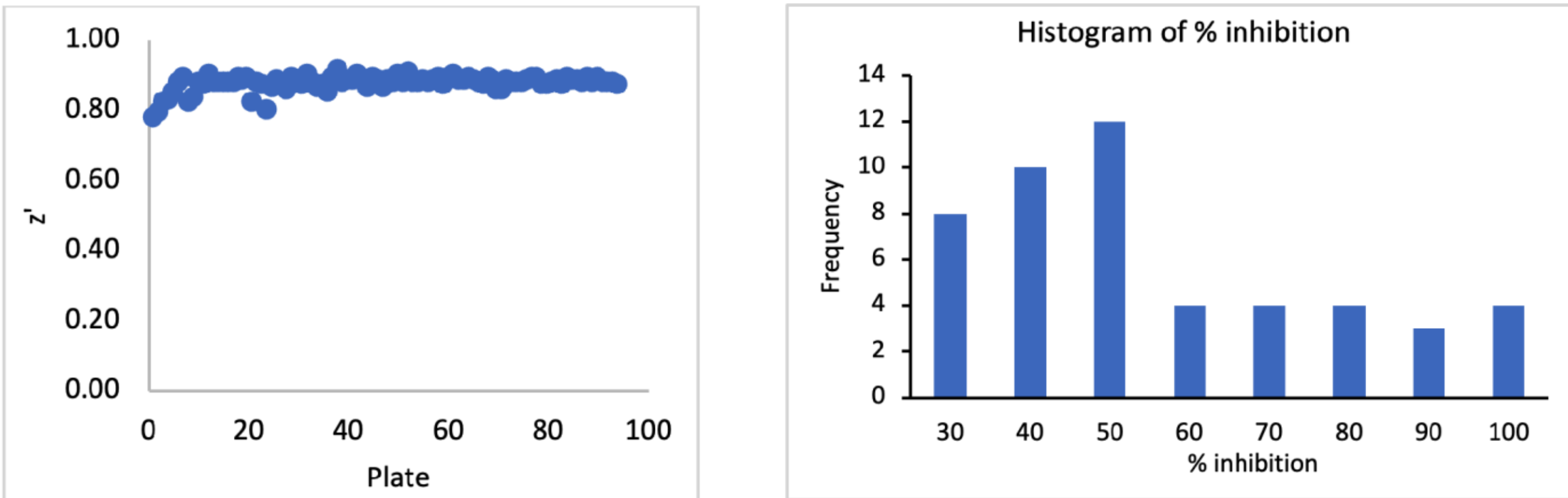

Figure S10. Distribution of z' values and % inhibition.

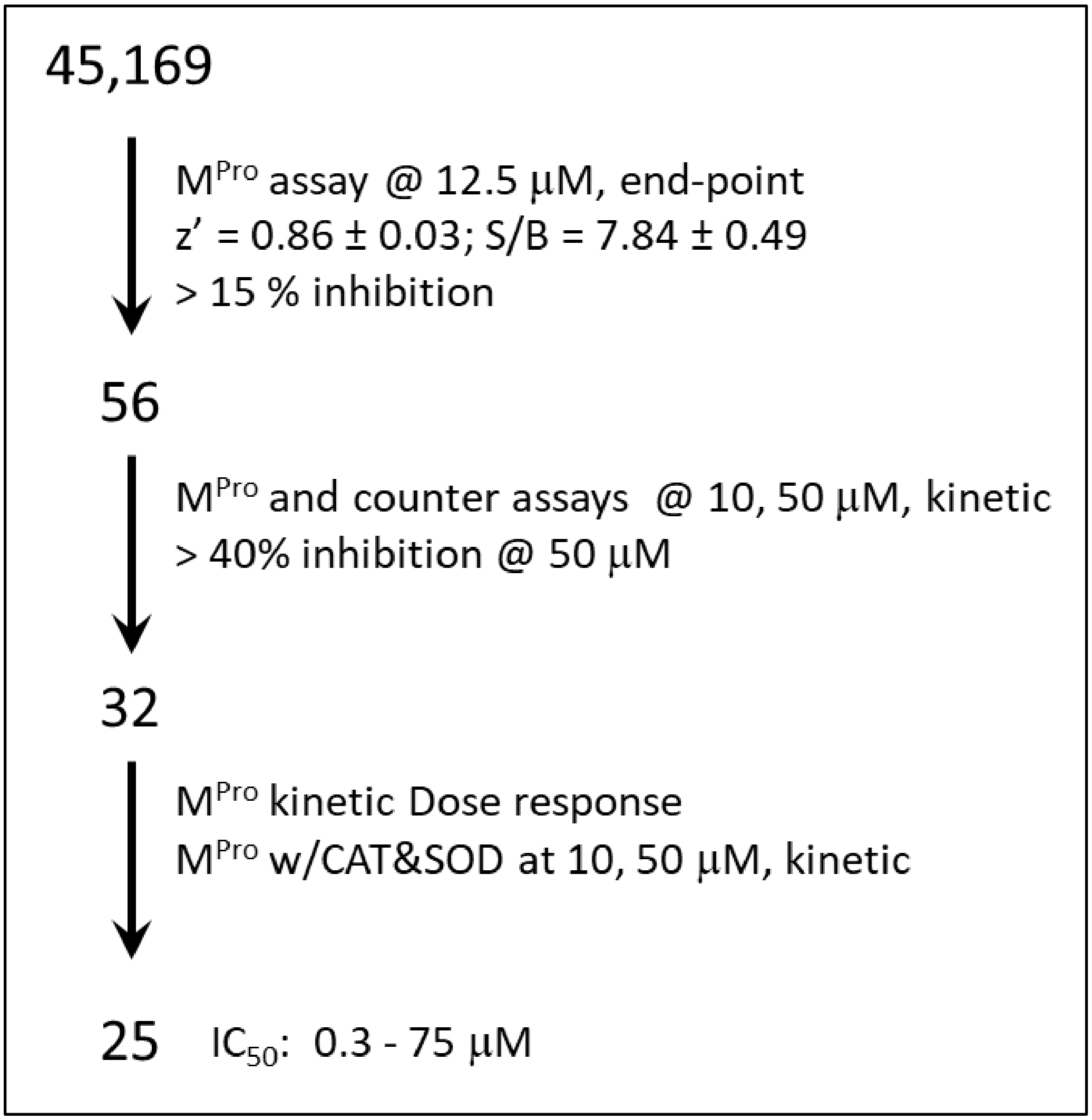

Figure S11. Hit selection flow for the 2nd compound screening campaign.

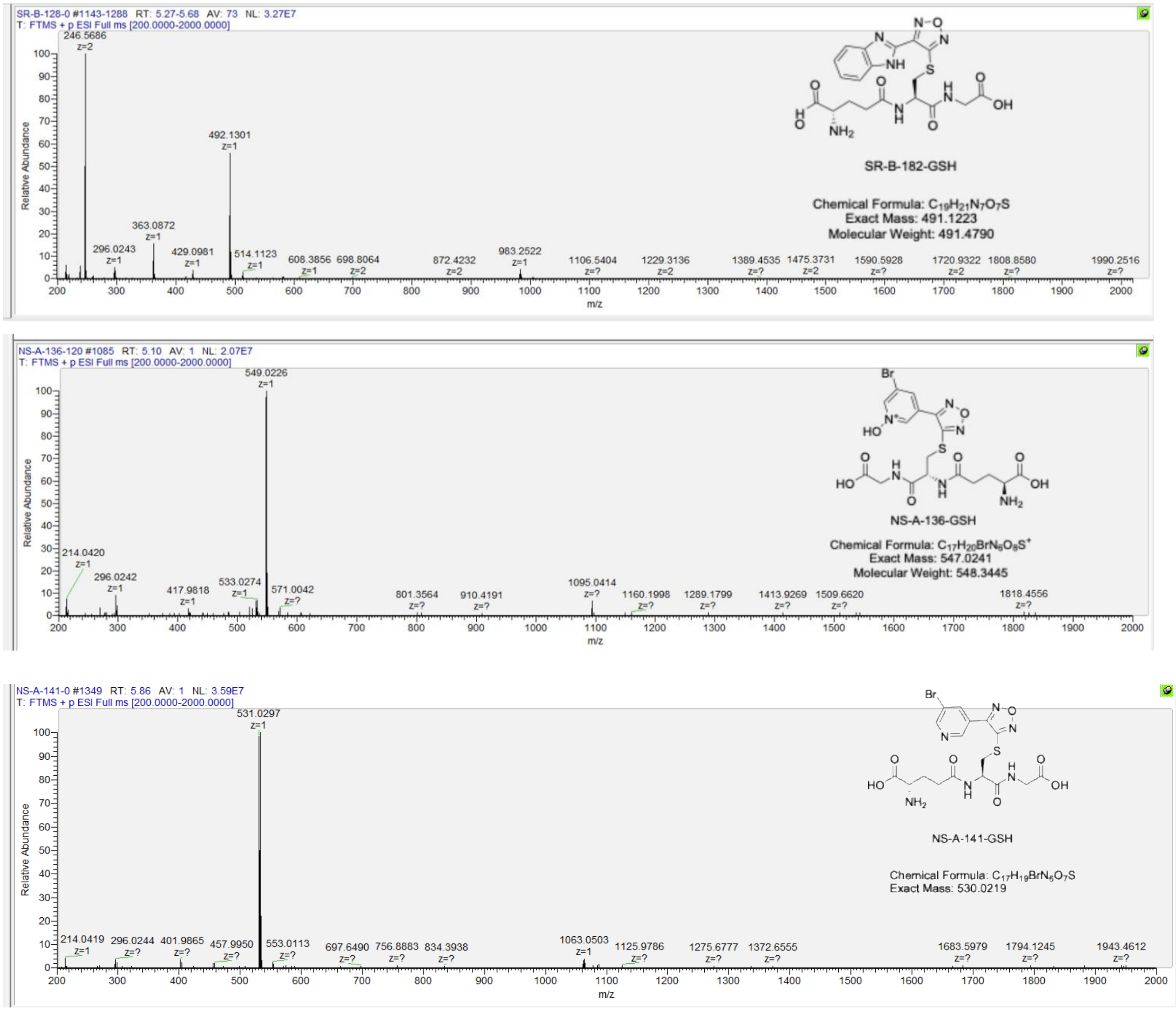

Figure S12. GSH stability of SR-B-182, NS-1-136 and NS-A-141. 20 uM of SR-B-182, NS-1-136 and NS-A-141 was incubated with 10 mM of GSH, their mass were analyzed by LC-MS/MS.

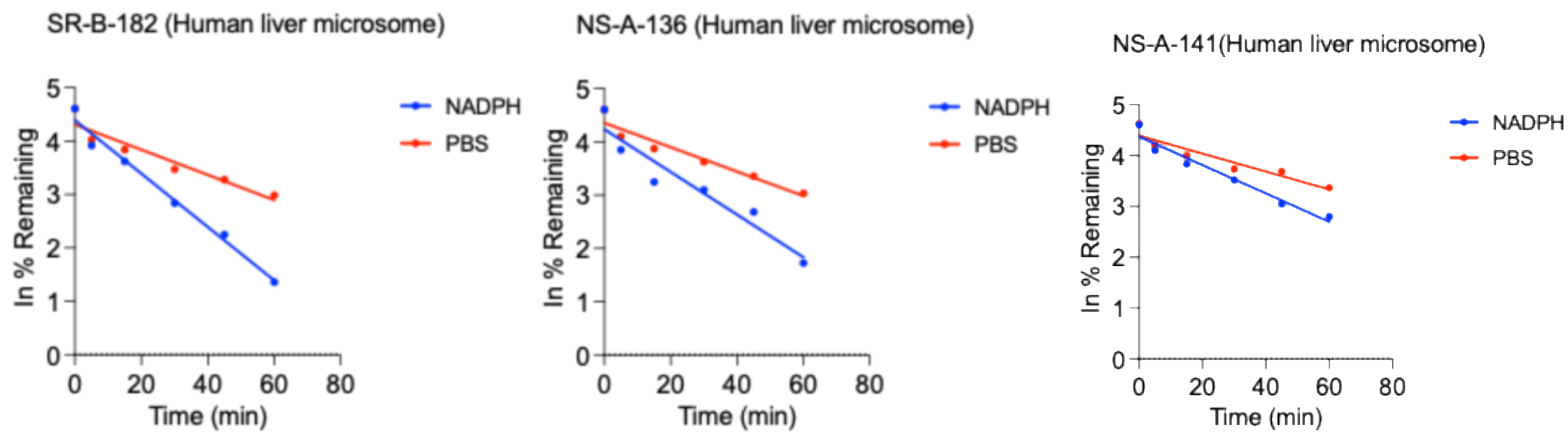

Compound	Half life $t_{1/2}$ (min) in Human liver microsome (With NADPH)	Half life $t_{1/2}$ (min) in Human liver microsome (Without NADPH)
SR-B-182	13.8	29.4
NS-A-136	17.3	30.4
NS-A-141	24.8	39.2

Figure S13. Metabolic stability of SR-B-182, NS-1-136 and NS-A-141 in Human Liver Microsome.

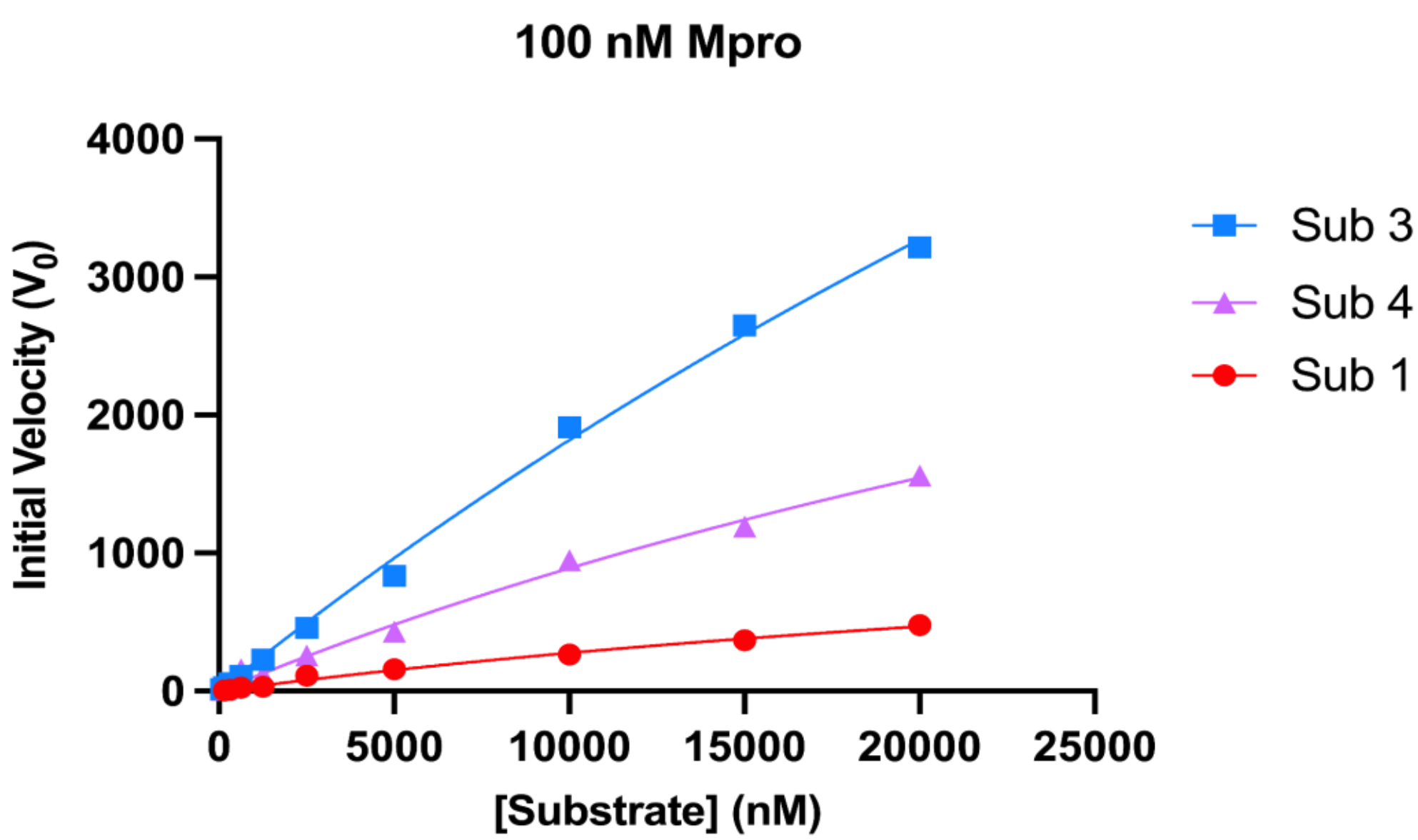

Figure S14. The initial velocity as a function of substrate concentration for Substrates 1, 3, and 4.

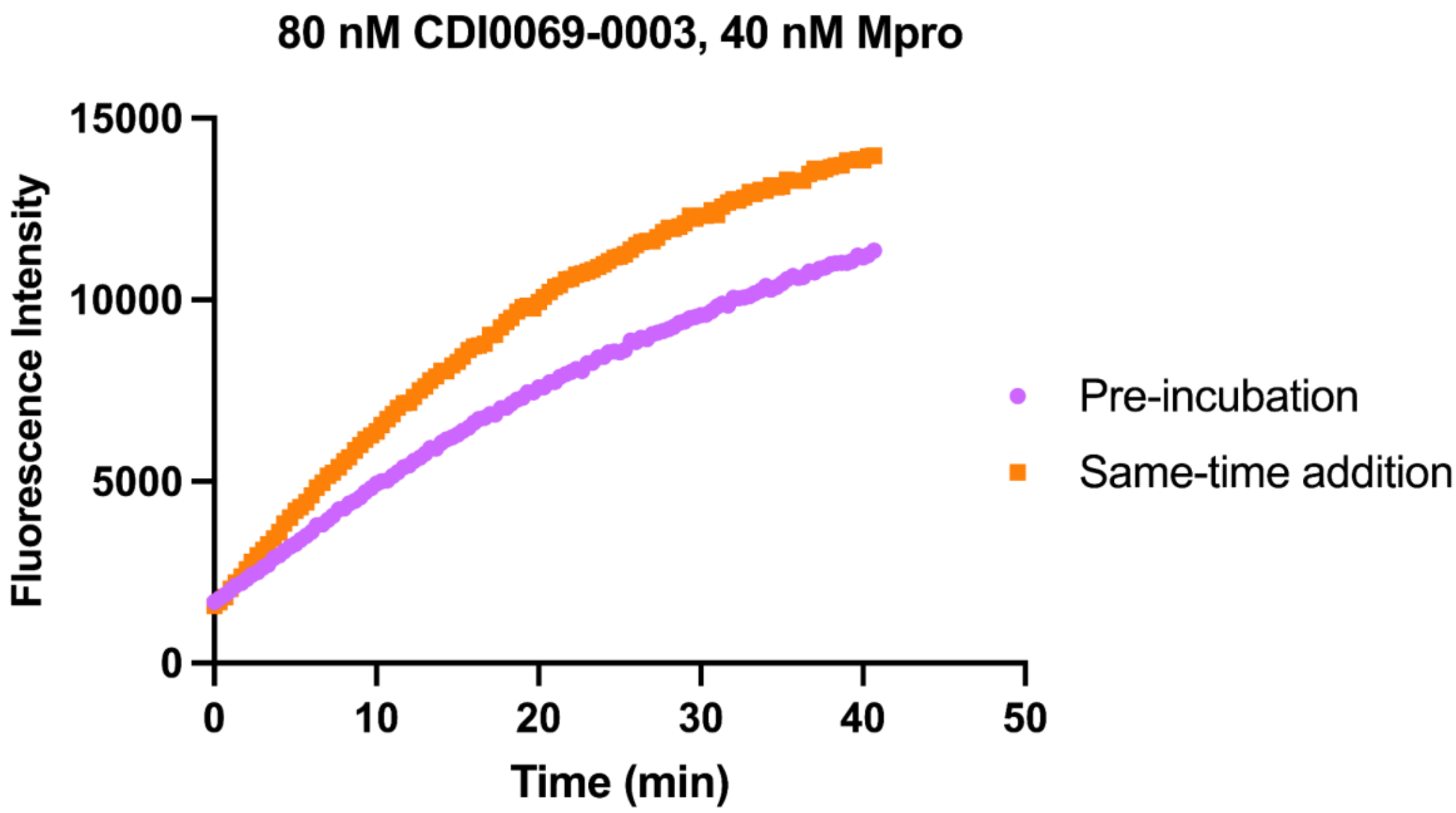

Figure S15. Inhibition activity of CDI0069-0003 against Mpro, indicating a reversible mechanism of action. Mpro was pre-incubated with CDI0069-0003 at a 1:2 enzyme-to-inhibitor molar ratio for 3 hours, and diluted to final assay concentrations of 40 nM Mpro and 80 nM inhibitor, followed by substrate addition. This pre-incubated condition was compared to a same-time addition control, in which 40 nM Mpro, 80 nM inhibitor, and substrate were combined simultaneously at the start of the assay.

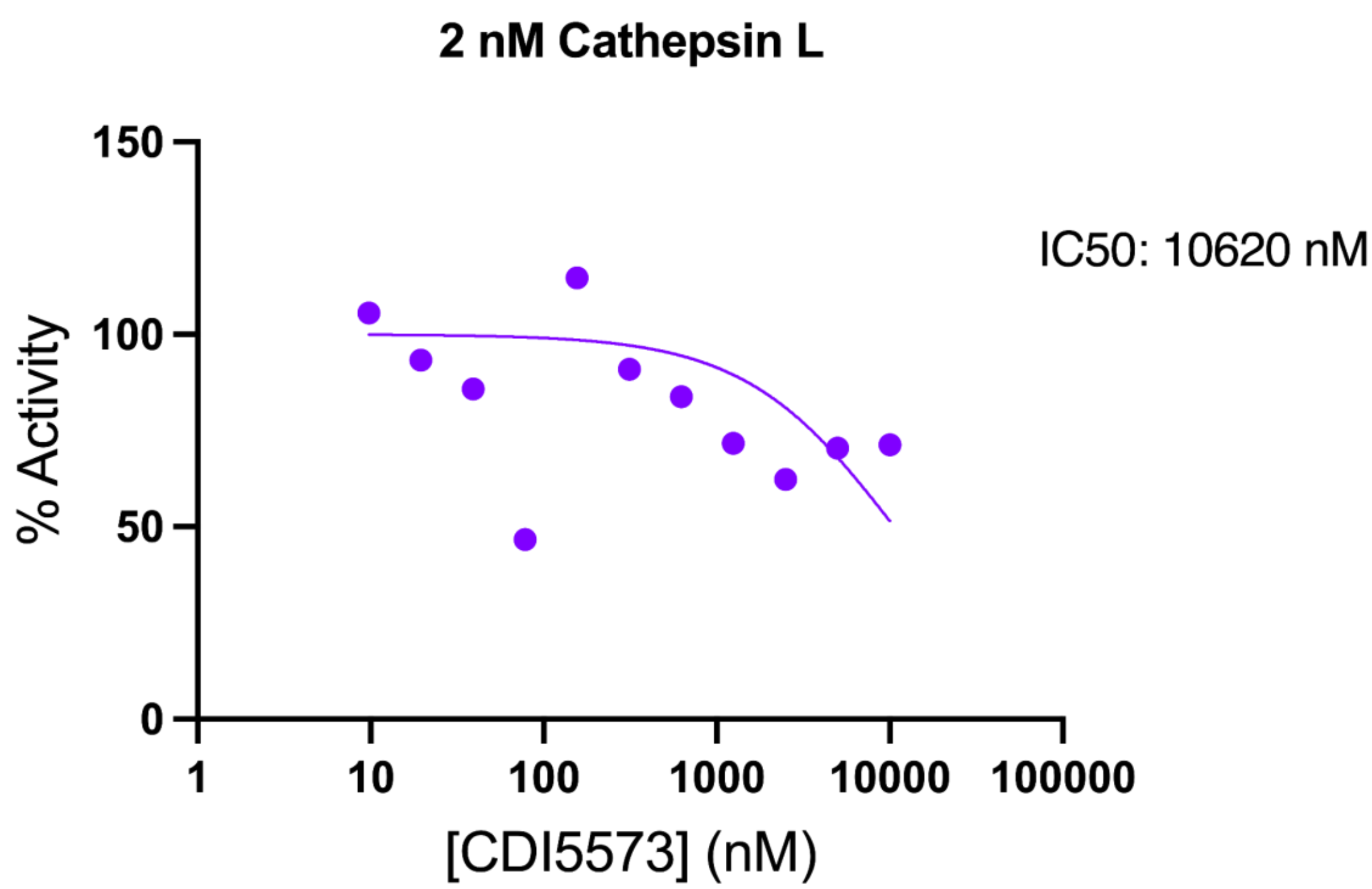

Figure S16. CDI5573 inhibition assay against Cathepsin L, a human cysteine protease, demonstrating that CDI5573 exhibits meaningful selectivity for Mpro over other cysteine proteases.

Supplementary Table 1: Data Collection and Refinement Statistics for M^{Pro}-CDI5573

PDB Entry	9BTE
Resolution range (Å)	42.05 - 1.6 (1.657 - 1.6)
Space group	I 1 2 1
Unit cell	51.3492 82.248 89.1954 90 96.8989 90
Total reflections	479954 (29538)
Unique reflections	48375 (4788)
Multiplicity	9.9 (6.2)
Completeness (%)	99.40 (98.92)
Mean I/sigma(I)	12.73 (1.41)
Wilson B-factor	17.20
R-merge	0.09655 (1.695)
R-meas	0.1014 (1.845)
R-pim	0.03026 (0.7157)
CC1/2	0.999 (0.506)
CC*	1 (0.82)
Reflections used in refinement	48224 (4777)
Reflections used for R-free	2452 (246)
R-work	0.2345 (0.4212)
R-free	0.2654 (0.4739)
CC(work)	0.956 (0.346)
CC(free)	0.939 (0.279)
Number of non-hydrogen atom	2613
macromolecules	2363
ligands	18
solvent	232
Protein residues	306
RMS(bonds)	0.009
RMS(angles)	1.11
Ramachandran favored (%)	96.71
Ramachandran allowed (%)	3.29
Ramachandran outliers (%)	0.00
Rotamer outliers (%)	0.00
Clashscore	10.65
Average B-factor	31.21
macromolecules	30.85
ligands	41.68
solvent	34.07
Number of TLS groups	1

Supplementary Table 2: Data Collection and Refinement Statistics for M^{Pro}-CDI8009

PDB Entry	9BST
Resolution range	42.15 - 1.6 (1.657 - 1.6)
pace group	I 1 2 1
Unit cell	51.49 82.1962 89.105 90 96.8115 90
otal reflections	93824 (8547)
Unique reflections	48108 (4694)
Multiplicity	2.0 (1.8)
Completeness (%)	98.93 (96.68)
Mean I/sigma(I)	9.29 (1.71)
Wilson B-factor	12.02
R-merge	0.0525 (0.3639)
R-meas	0.07424 (0.5146)
R-pim	0.0525 (0.3639)
CC1/2	0.998 (0.881)
CC*	1 (0.968)
Reflections used in refinement	48057 (4694)
Reflections used for R-free	2436 (243)
R-work	0.2627 (0.4946)
R-free	0.2954 (0.5232)
CC(work)	0.947 (0.318)
CC(free)	0.923 (0.230)
Number of non-hydrogen atoms	2598
macromolecules	2363
ligands	15
solvent	220
rotein residues	306
RMS(bonds)	0.009
RMS(angles)	1.72
Ramachandran favored (%)	96.38
Ramachandran allowed (%)	3.29
Ramachandran outliers (%)	0.33
Rotamer outliers (%)	0.00
Clashscore	9.38
verage B-factor	25.83
macromolecules	25.38
ligands	32.43
solvent	30.15
Number of TLS groups	1

Supplementary Synthesis Details:

All reagents and solvents for synthesis were purchased from commercial sources and used without purification. All glassware was flame-dried prior to use. Thin layer chromatography (TLC) was carried out on aluminum plates coated with 60 F254 silica gel. TLC plates were visualized under UV light (254 or 365 nm) or stained with 5% phosphomolybdic acid. Normal phase column chromatography was carried out using a Yamazen Smart Flash AKROS system. Analytical reverse-phase high-pressure liquid chromatography (RP-HPLC) was carried out on a Shimadzu LC20 HPLC system with an analytical C18 column. The mobile phases were H2O with 0.1% formic acid (A) and acetonitrile with 0.1% formic acid (B) if not mentioned otherwise. NMR spectra were recorded on a Bruker AVANCE Neo 400 MHz or Varian INOVA 300 MHz spectrometer in specified deuterated solvents. High-resolution electrospray ionization mass spectrometry (HRMS-ESI) was carried out on a Themo Scientific QExactive Focus system. Purities of assayed compounds were greater than 95% as determined by reverse-phase high performance liquid chromatography analysis.

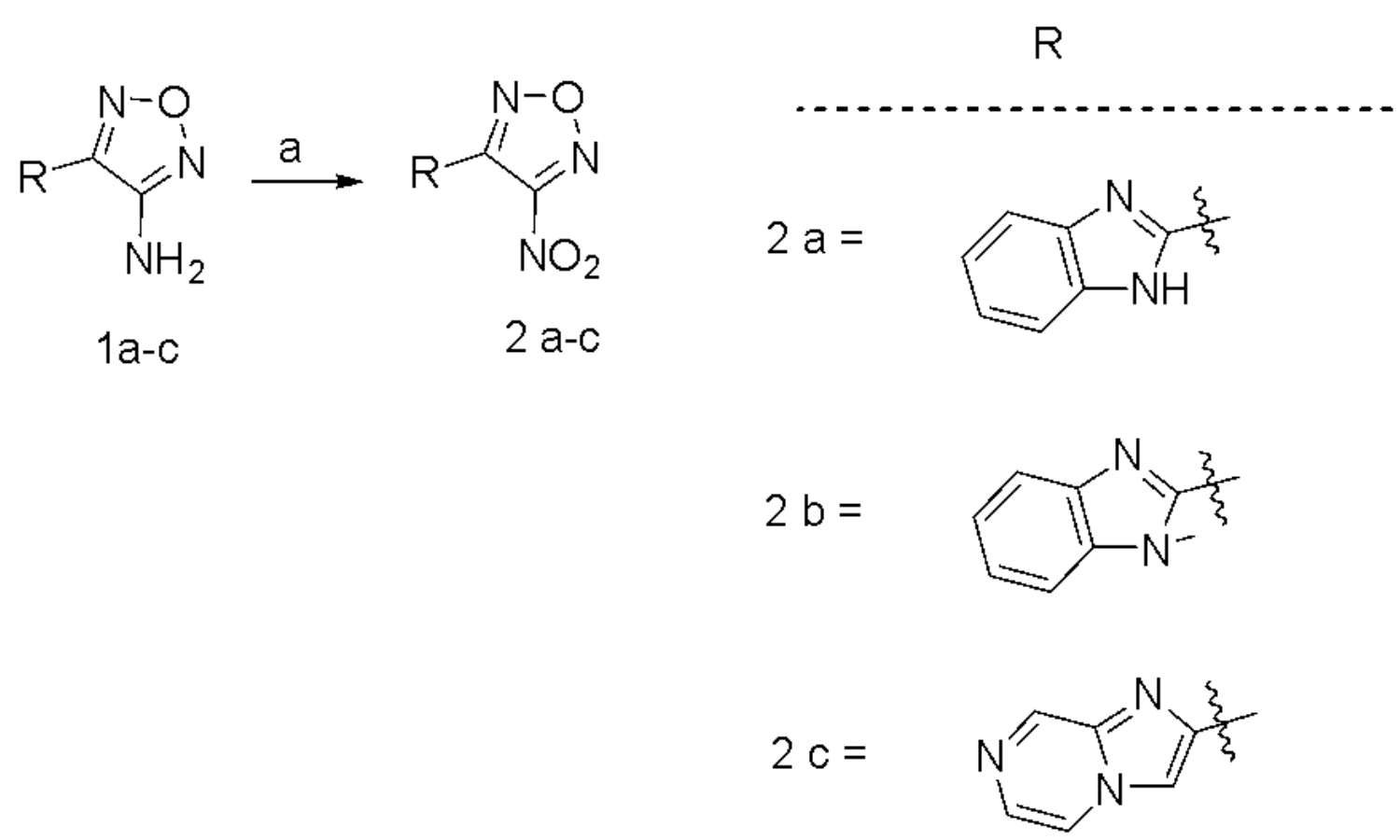

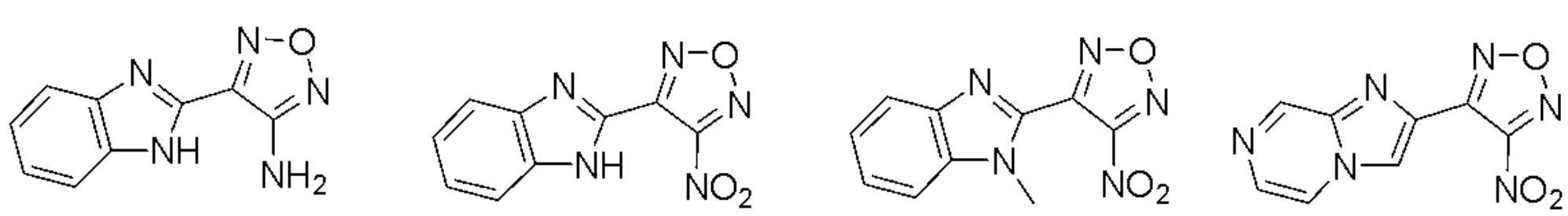

1a (SR-B-163) **2a (SR-B-182)** **2b (SR-B-196)** **2c (SR-B-188)**

Scheme S1. The synthesis of SR-B-182 (2a), SR-B-196 (2b), SR-B-188 (2c).
Reagents and conditions: (a) Trifluoro Acetic anhydride, 35% H_2O_2, CH_2Cl_2, 50 °C, 2 h.

General procedure for synthesis of 2a-c:
A solution of trifluoroacetic anhydride (6 eq) in CH_2Cl_2 (10 volumes) was added dropwise to 35% H_2O_2 (6 eq) at 0 °C and stirred for 1.5 h. Then compound 1a-c (1 eq) in CH_2Cl_2 (10 volumes) was added dropwise to above reaction mixture and stirred for 2 h at 50 ^{0}C. After completion of the reaction, the mixture was quenched by adding water and extracted with dichloromethane (3x20 mL). The combined organic layers were dried over Na_2SO_4, filtered and concentrated under

vacuum. Purify the residue by flash chromatography (0-100% EA in Hexane as the eluent) yielded resulting products in good yields.

3-(1H-benzo[d]imidazol-2-yl)-4-nitro-1,2,5-oxadiazole 2a (SR-B-182):
By following the above general procedure, SR-B-182 compound was synthesized as a yellow solid in good yield (88%).
^{1}H NMR (400 MHz, CD_3OD) δ 7.64 – 7.56 (m, 2H), 7.30 – 7.24 (m, 2H). ^{13}C NMR (101 MHz, CD_3OD) δ 159.22, 142.07, 138.76, 135.46, 125.01, 116.26. HRMS (ESI) calculated for C9H5N5O3 $[M+H]^+$ 232.0400; Found 232.0464.

3-(1-methyl-1H-benzo[d]imidazol-2-yl)-4-nitro-1,2,5-oxadiazole 2b (SR-B-188):
By following the above general procedure, SR-B-188 compound was synthesized as a white solid in good yield(85%).
^{1}H NMR (400 MHz, CD_3OD) δ 9.04 (s, 1H), 8.70 (s, 1H), 8.51 (dd, J = 4.6, 1.2 Hz, 1H), 7.92 – 7.89 (m, 1H). ^{13}C NMR (101 MHz, DMSO-d_6) δ 145.62, 144.63, 140.46, 130.28, 129.67, 121.14, 118.42. HRMS (ESI) calculated for C8H4N6O3 $[M+H]^+$ 233.0300; Found 233.0315.

3-(imidazo[1,2-a]pyrazin-2-yl)-4-nitro-1,2,5-oxadiazole 2c (SR-B-196):
By following the above general procedure, SR-B-196 compound was synthesized as a white solid in good yield(82%).
^{1}H NMR (400 MHz, $CDCl_3$) δ 7.76 (d, J = 8.2 Hz, 1H), 7.43 – 7.35 (m, 2H), 7.29 (ddd, J = 8.2, 6.4, 1.8 Hz, 1H), 4.06 (s, 3H). ^{13}C NMR (101 MHz, $CDCl_3$) δ 143.00, 141.00, 136.41, 135.79, 125.79, 123.87, 121.54, 110.24, 31.95. HRMS (ESI) calculated for C8H4N6O3 $[M+H]^+$ 246.0500; Found 246.0621

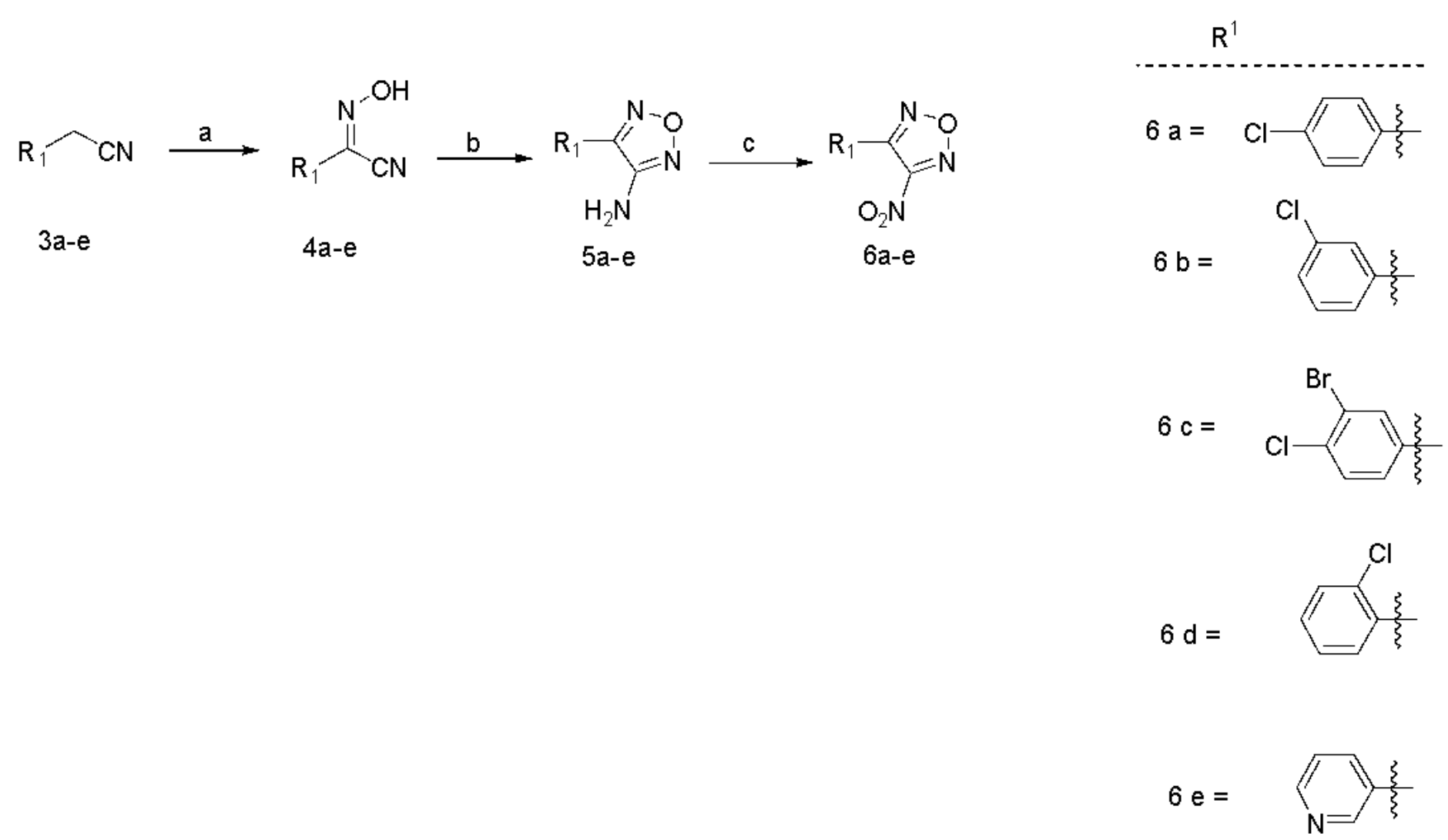

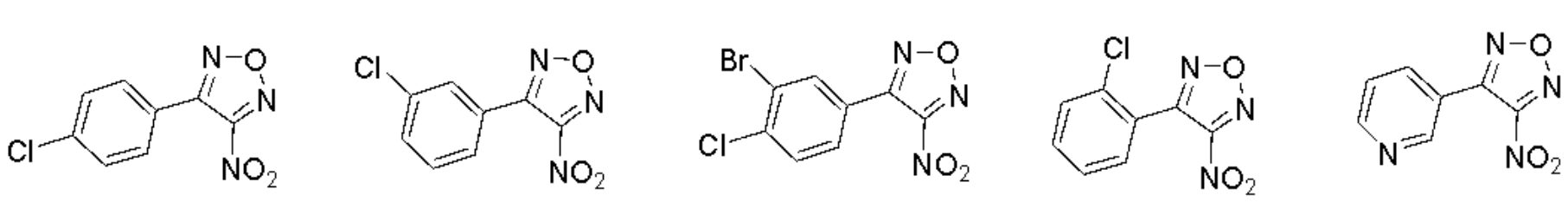

6a (SR-C-44) 6b (SR-C-46) 6c (SR-C-63) 6d (NS-A-135) 6e (NS-A-148)

Scheme S2. The synthesis of SR-C-44 (6a), SR-C-46 (6b), SR-C-63 (6c), NS-A-135 (6d) and NS-

A-148 (6e).

Reagents and conditions: (a) t-BuOK, tert-butyl nitrite, DMF 0 °C to rt, 1 h (b) i. 50 % NH_2OH, MeOH, 40 °C, 16 h; ii. CDI, THF, rt, 2 h (c) Trifluoro Acetic anhydride, 35% H_2O_2, CH_2Cl_2, 50 °C, 2 h.

General procedure for synthesis of 4a-e:
A solution of nitrile substrate 3a-e (1 eq) in DMF was purged with N_2 and the mixture was cooled to 0 °C using ice water bath. t-BuOK (1.2 eq) was added to the reaction mixture, resulting in a color change from colorless to yellow. After 5 min, tert-butyl nitrite (2 eq) was added, resulting in a deep red color, and the reaction mixture was allowed to warm to room temperature for 1h. After completion of the reaction, 1N HCl was added until the red color faded, and the reaction mixture was extracted with water and EtOAc (2x100 mL). The combined organic layers were dried over Na_2SO_4, filtered and concentrated under vacuum. Crude was directly used for next step without further purification.

General procedure for synthesis of 5a-e:
To a stirred solution of cyano-oxime 4a-e (1 eq) in MeOH was added 50 % NH_2OH solution (2 eq) at rt and stirred for 16h at 40 ^{0}C. After completion of the reaction, the reaction mixture was cooled to rt, concentrated in vacuo to remove methanol. The crude residue was concentrated two additional times with dichloromethane until a white solid was obtained. The white solid was dissolved in THF then CDI (2 eq) was added, and the reaction mixture was stirred at room temperature for 2 h. The starting material consumption was confirmed by TLC. The reaction mixture was concentrated and purified directly by flash chromatography (0-100% EA in Hexane as the eluent) yielded resulting products in good yields.

4-(4-chlorophenyl)-1,2,5-oxadiazol-3-amine (5a):
According to the general procedure for synthesis of 5a-e, 5a compound was synthesized as a white solid in good yield (82%).
^{1}H NMR (400 MHz, DMSO-d_6) δ 7.82 – 7.75 (m, 2H), 7.67 – 7.60 (m, 2H), 6.25 (s, 2H).

4-(3-chlorophenyl)-1,2,5-oxadiazol-3-amine (5b):
According to the general procedure for synthesis of 5a-e, 5c compound was synthesized as a white solid in good yield (85%).
^{1}H NMR (400 MHz, $CDCl_3$) δ 7.67 (t, J = 1.6 Hz, 1H), 7.60 – 7.50 (m, 1H), 7.49 – 7.35 (m, 2H).

4-(3-bromo-4-chlorophenyl)-1,2,5-oxadiazol-3-amine (5c):
According to the general procedure for synthesis of 5a-e, 5b compound was synthesized as a white solid in good yield (87%).
^{1}H NMR (400 MHz, DMSO-d_6) δ 8.10 (d, J = 1.7 Hz, 1H), 7.80 (qd, J = 8.4, 3.1 Hz, 2H), 6.32 (s, 2H).

4-(2-chlorophenyl)-1,2,5-oxadiazol-3-amine (5d):
According to the general procedure for synthesis of 5a-e, 5d compound was synthesized as a white solid in good yield (86%).
^{1}H NMR (400 MHz, DMSO-d_6) δ 7.71 – 7.64 (m, 1H), 7.64 – 7.46 (m, 3H), 6.16 (s, 2H).^{13}C NMR (101 MHz, DMSO-d_6) δ 155.86, 146.05, 133.23, 132.08, 131.96, 130.06, 127.61, 124.62.

4-(pyridin-3-yl)-1,2,5-oxadiazol-3-amine (5e):

According to the general procedure for synthesis of 5a-e, 5e compound was synthesized as a white solid in good yield (81%).
^{1}H NMR (400 MHz, DMSO-d_6) δ 8.94 (dd, J = 2.3, 0.9 Hz, 1H), 8.75 (dd, J = 4.9, 1.6 Hz, 1H), 8.20 – 8.12 (m, 1H), 7.63 – 7.55 (m, 1H), 6.33 (s, 2H). 13C NMR (101 MHz, DMSO-d_6) δ 155.45, 151.11, 148.16, 145.00, 135.52, 124.06, 121.99.

3-(4-chlorophenyl)-4-nitro-1,2,5-oxadiazole 6a (SR-C-44):
According to the general procedure for synthesis of 2a-c, SR-C-44 compound was synthesized as a white solid in good yield (78%).
^{1}H NMR (400 MHz, $CDCl_3$) δ 7.82 (d, J = 8.6 Hz, 2H), 7.53 – 7.42 (m, 2H). HRMS (ESI) calculated for C8H4ClN3O3 $[M+H]^+$ 225.9900; Found 225.9913

3-(3-chlorophenyl)-4-nitro-1,2,5-oxadiazole 6b (SR-C-46):
According to the general procedure for synthesis of 2a-c, SR-C-46 compound was synthesized as a white solid in good yield (81%).
^{1}H NMR (400 MHz, $CDCl_3$) δ 7.88 (t, J = 1.7 Hz, 1H), 7.76 (d, J = 7.7 Hz, 1H), 7.54 (ddd, J = 8.1, 2.0, 1.1 Hz, 1H), 7.45 (t, J = 7.9 Hz, 1H). HRMS (ESI) calculated for C8H4ClN3O3 $[M+H]^+$ 225.9900; Found 225.9912

3-(3-bromo-4-chlorophenyl)-4-nitro-1,2,5-oxadiazole 6c (SR-C-63):
According to the general procedure for synthesis of 2a-c, SR-C-63 compound was synthesized as a white solid in good yield (83%).
^{1}H NMR (400 MHz, $CDCl_3$) δ 7.96 (d, J = 1.9 Hz, 1H), 7.62 – 7.51 (m, 2H). ^{13}C NMR (101 MHz, $CDCl_3$) δ 158.60, 147.39, 139.11, 134.25, 131.11, 128.92, 123.65, 121.61. HRMS (ESI) calculated for C8H3BrClN3O3 $[M+H]^+$ 303.9000; Found 303.9012

3-(2-chlorophenyl)-4-nitro-1,2,5-oxadiazole 6d (NS-A-135):
According to the general procedure for synthesis of 2a-c, NS-A-135 compound was synthesized as a white solid in good yield (77%).
^{1}H NMR (400 MHz, $CDCl_3$) δ 7.61 – 7.51 (m, 2H), 7.45 – 7.37 (m, 1H), 7.37 – 7.30 (m, 1H). ^{13}C NMR (101 MHz, $CDCl_3$) δ 170.36, 140.03, 133.80, 132.66, 131.51, 130.17, 127.39, 122.45. HRMS (ESI) calculated for C8H3ClN3O3 $[M+H]^+$ 223.9857; Found 224.0043.

3-nitro-4-(pyridin-3-yl)-1,2,5-oxadiazole 6e (NS-A-148):
According to the general procedure for synthesis of 2a-c, NS-A-148 compound was synthesized as a white solid in good yield (75%).
^{1}H NMR (400 MHz, $CDCl_3$) δ 8.98 (d, J = 2.3 Hz, 1H), 8.86 (dd, J = 4.9, 1.7 Hz, 1H), 8.11 – 8.03 (m, 1H), 7.57 – 7.48 (m, 1H). ^{13}C NMR (101 MHz, $CDCl_3$) δ 153.00, 149.69, 147.30, 136.79, 123.83, 118.83. HRMS (ESI) calculated for C7H5N4O3 $[M+H]^+$ 193.0300; Found 193.0355.

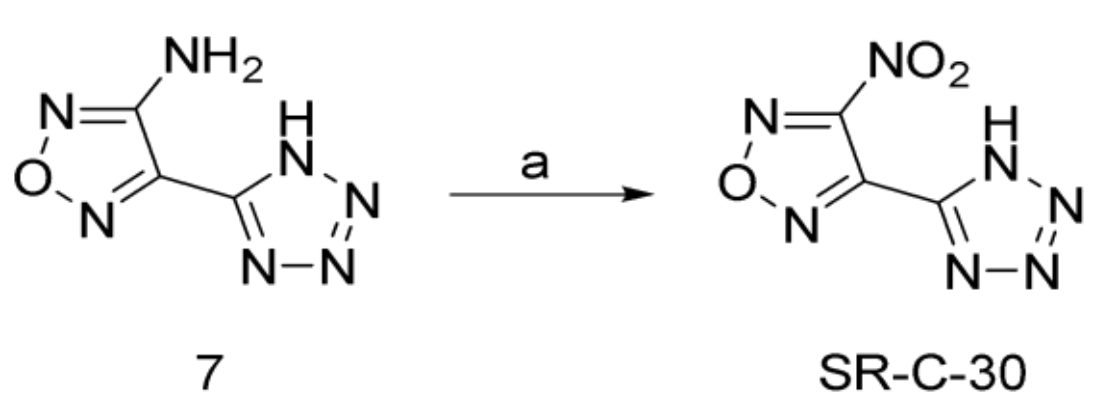

Scheme S3. The synthesis of SR-C-30. Reagents and conditions: (a) Conc. H_2SO_4, 35% H_2O_2, 50 °C, 2 h.

3-nitro-4-(1H-tetrazol-5-yl)-1,2,5-oxadiazole (SR-C-30):
A solution of Conc. H_2SO_4 (1.5 mL) was added dropwise to 35% H_2O_2 (1.5 mL) at 0 °C and stirred for 5 min. Then compound 7 (100 mg, 0.65 mmol) was added to above reaction mixture and stirred for 2 h at 50 ^{0}C. After completion of the reaction, the mixture was quenched by adding water and extracted with EtOAc (3x20 mL). The combined organic layers were dried over Na_2SO_4, filtered and concentrated under vacuum. Purify the residue by flash chromatography (0-100% EA in Hexane as the eluent) yielded resulting product in moderate yield (55%).
HRMS (ESI) calculated for C3HN7O3 $[M-H]^+$ 182.0100; Found 182.0058.

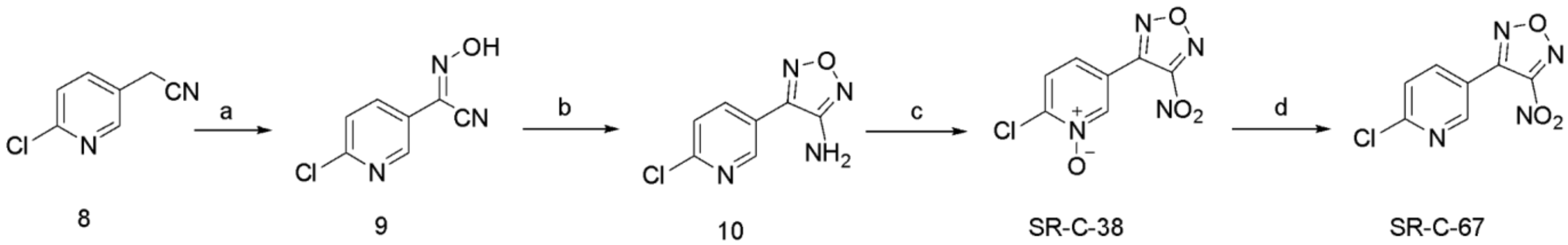

Scheme S4. The synthesis of SR-C-38 and SR-C-67.

Reagents and conditions: (a) t-BuOK, tert-butyl nitrite, DMF 0 °C to rt, 1 h (b) i. 50 % NH_2OH, MeOH, 40 °C, 16 h; ii. CDI, THF, rt, 2 h (c) Trifluoro Acetic anhydride, 35% H_2O_2, CH_2Cl_2, 50 °C, 2 h (d) PBr_3, $CHCl_3$, 60 °C, 16 h.

(Z)-6-chloro-N-hydroxynicotinimidoyl cyanide (9):
According to the general procedure for synthesis of 4a-e, compound 9 was synthesized as a white solid. Crude was directly used for next step without further purification.

4-(6-chloropyridin-3-yl)-1,2,5-oxadiazol-3-amine (10):
According to the general procedure for synthesis of 5a-e, compound 10 was synthesized as a white solid in good yield (78%).
^{1}H NMR (400 MHz, DMSO-d_6) δ 8.78 (d, J = 2.0 Hz, 1H), 8.22 (dd, J = 8.3, 2.5 Hz, 1H), 7.74 (d, J = 8.3 Hz, 1H), 6.37 (s, 2H).

2-chloro-5-(4-nitro-1,2,5-oxadiazol-3-yl)pyridine 1-oxide (SR-C-38):
According to the general procedure for synthesis of 2a-c, SR-C-38 compound was synthesized as a white solid in good yield (72%).
^{1}H NMR (400 MHz, DMSO-d_6) δ 8.91 (d, J = 1.6 Hz, 1H), 8.08 (d, J = 8.5 Hz, 1H), 7.74 (dd, J = 8.5, 1.8 Hz, 1H). HRMS (ESI) calculated for C8H4N6O3 $[M+H]^+$ 242.9800; Found 242.9915

3-(6-chloropyridin-3-yl)-4-nitro-1,2,5-oxadiazole (SR-C-67):
According to the general procedure for synthesis of 2a-c, SR-C-38 compound was synthesized as a white solid in good yield (72%).
To a stirred solution of compound SR-C-38 (0.050 g, 0.174 mmol) in chloroform (2 mL) at 0 °C, PBr_3 (0.2 mL) was added slowly to the above reaction mixture and stirred for 12 h at 60 °C. After the reaction was completed, the mixture was cautiously poured into 20 mL ice-water and the solution neutralized with sodium bicarbonate. The layers were separated, and the water-layer was extracted with chloroform (3 x 15 mL). The organic layers were dried over sodium sulphate and concentrated in vacuo. The residue was purified by flash chromatography (0-100% EA in Hexane as the eluent) yielding resulting product SR-C-67 as a white solid (37 mg, 80 %).

^{1}H NMR (400 MHz, $CDCl_3$) δ 8.74 – 8.65 (m, 1H), 8.01 (dd, J = 8.4, 2.5 Hz, 1H), 7.51 (dd, J = 8.4, 0.5 Hz, 1H). ^{13}C NMR (101 MHz, $CDCl_3$) δ 158.67, 155.21, 149.71, 146.50, 139.27, 124.82, 117.64. HRMS (ESI) calculated for C7H3ClN4O3 $[M+H]^+$ 226.9900; Found 226.9966

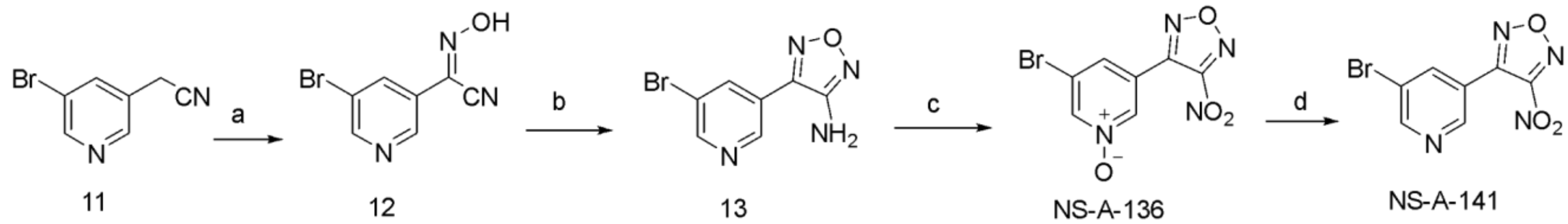

Scheme S5. The synthesis of NS-A-136 and NS-A-141.

Reagents and conditions: (a) t-BuOK, tert-butyl nitrite, DMF 0 °C to rt, 1 h (b) i. 50 % NH_2OH, MeOH, 40 °C, 16 h; ii. CDI, THF, rt, 2 h (c) Trifluoro Acetic anhydride, 35% H_2O_2, CH_2Cl_2, 50 °C, 2 h (d) PBr_3, $CHCl_3$, 60 °C, 16 h.

(Z)-5-bromo-N-hydroxynicotinimidoyl cyanide (12):
According to the general procedure for synthesis of 4a-e, compound 12 was synthesized as a white solid. Crude was directly used for next step without further purification.

4-(5-bromopyridin-3-yl)-1,2,5-oxadiazol-3-amine (13):
According to the general procedure for synthesis of 5a-e, compound 13 was synthesized as a white solid in good yield (72%).
1H NMR (400 MHz, DMSO-d_6) δ 8.90 (dd, J = 4.4, 2.0 Hz, 2H), 8.38 (t, J = 2.1 Hz, 1H), 6.41 (s, 2H). 13C NMR (101 MHz, DMSO-d_6) δ 155.44, 151.76, 146.86, 143.97, 137.83, 123.61, 120.42.

3-bromo-5-(4-nitro-1,2,5-oxadiazol-3-yl)pyridine 1-oxide (NS-A-136):
According to the general procedure for synthesis of 2a-c, NS-A-136 compound was synthesized as a white solid in good yield (75%).
^{1}H NMR (400 MHz, $CDCl_3$) δ 8.53 (t, J = 1.5 Hz, 1H), 8.50 (t, J = 1.5 Hz, 1H), 7.75 (t, J = 1.5 Hz, 1H). ^{13}C NMR (101 MHz, $CDCl_3$) δ 158.57, 144.75, 143.31, 138.26, 128.27, 122.57, 121.25.
HRMS (ESI) calculated for C7H3BrN4O4 $[M+H]^+$ 286.9410; Found 286.9403.

3-(5-bromopyridin-3-yl)-4-nitro-1,2,5-oxadiazole (NS-A-141):
According to the general procedure for synthesis of SR-C-67, NS-A-141 compound was synthesized as a white solid in good yield (78%).
^{1}H NMR (400 MHz, $CDCl_3$) δ 8.95 – 8.86 (m, 2H), 8.24 (t, J = 2.1 Hz, 1H). ^{13}C NMR (101 MHz, $CDCl_3$) δ 154.23, 147.71, 146.25, 139.20, 121.03, 120.09. HRMS (ESI) calculated for C7H4BrN4O3 $[M+H]^+$ 270.9400; Found 270.9459.

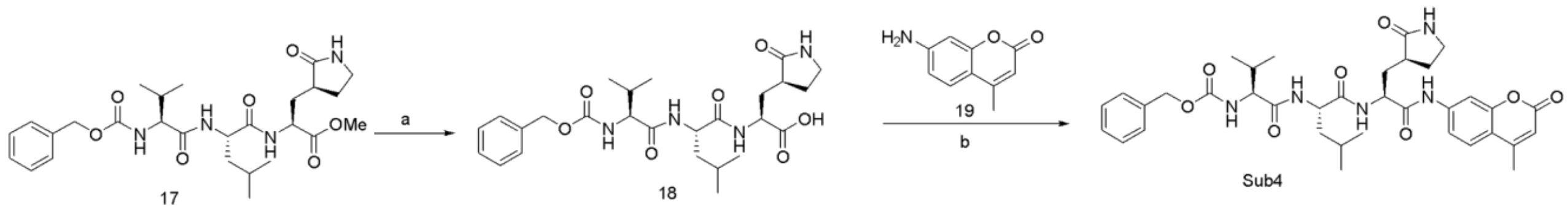

Scheme S6 . The synthesis of Sub4.

Reagents and conditions: (a) $LiOH.H_2O$, THF: H_2O , 0 °C to rt, 16 h (b) T3P, NMM, ACN, 0 °C to rt, 16 h.

(5S,8S,11S)-8-isobutyl-5-isopropyl-3,6,9-trioxo-11-(((S)-2-oxopyrrolidin-3-yl)methyl)-1-phenyl-2-oxa-4,7,10-triazadodecan-12-oic acid (18):

Compound 17 (300 mg, 0.564 mmol) was dissolved in THF/H_2O (1:1, 10 mL). $LiOH.H_2O$ (34 mg, 0.846 mmol) was added at 0 °C. The mixture was stirred at room temperature overnight. Then THF was removed on vacuum, and the aqueous layer was acidified with 1 M HCl and extracted with dichloromethane (3 x 10 mL). The organic layer was dried over anhydrous Na_2SO_4 and concentrated to yield 18 as white solid (250 mg). Crude compound was directly used for next without further purification.

benzyl ((S)-3-methyl-1-(((S)-4-methyl-1-(((S)-1-((4-methyl-2-oxo-2H-chromen-7-yl)amino)-1-oxo-3-((S)-2-oxopyrrolidin-3-yl)propan-2-yl)amino)-1-oxopentan-2-yl)amino)-1-oxobutan-2-yl)carbamate (Sub4):

Compound 18 (120 mg, 0.232 mmol) was dissolved in anhydrous acetonitrile (4.5 mL) under a nitrogen atmosphere and cooled to 0 °C, after which NMM (~31 µL ,0.279 mmol) was added dropwise followed by slow addition of T3P (50% w/w in EtOAc, 0.557 mmol, 2.4 equiv, ~0.33 mL); the reaction mixture was stirred at 0 °C for 15–20 min to allow activation of the carboxylic acid, then coumarin (49 mg, 0.279 mmol) was added portionwise at 0 °C, and the reaction was allowed to warm to room temperature and stirred for 3–6 h until complete as monitored by TLC, after which the mixture was diluted with ethyl acetate (20 mL) and washed successively with saturated aqueous $NaHCO_3$ (2 × 15 mL), 1 M HCl (1 × 15 mL), and brine (1 × 15 mL), dried over anhydrous Na_2SO_4, filtered, and concentrated under reduced pressure to afford the crude product, which was purified by flash chromatography (0-10% MeOH-DCM as the eluent) yielded resulting product Sub4 in moderate yield (45%).

^{1}H NMR (400 MHz, $CDCl_3$) δ 9.51 – 9.43 (m, 1H), 8.30 (s, 1H), 8.02 – 7.81 (m, 1H), 7.78 – 7.58 (m, 1H), 7.52 – 7.42 (m, 1H), 7.32 (d, J = 5.8 Hz, 5H), 6.17 (s, 1H), 5.44 (s, 1H), 5.11 (q, J = 11.3 Hz, 2H), 4.87 – 4.51 (m, 1H), 4.37 (d, J = 6.9 Hz, 1H), 4.06 (t, J = 6.8 Hz, 1H), 3.75 (s, 1H), 3.36 (s, 2H), 2.43 – 2.35 (m, 4H), 2.22 – 2.12 (m, 2H), 1.78 (d, J = 80.1 Hz, 6H), 0.95 (ddd, J = 23.2, 13.3, 6.2 Hz, 12H).

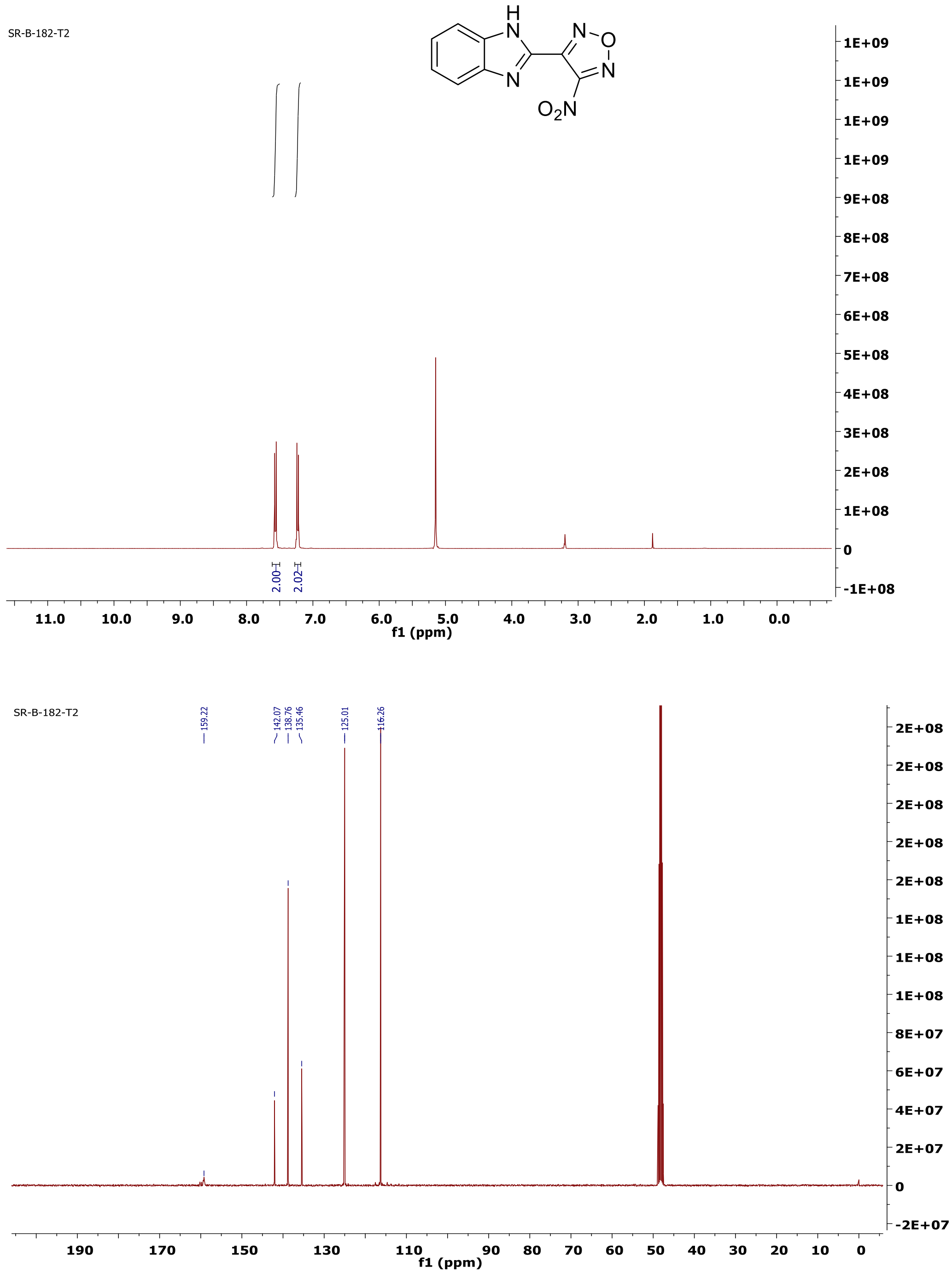

Figure S17. ^{1}H NMR and ^{13}C NMR spectra of SR-B-182 in CD_3OD

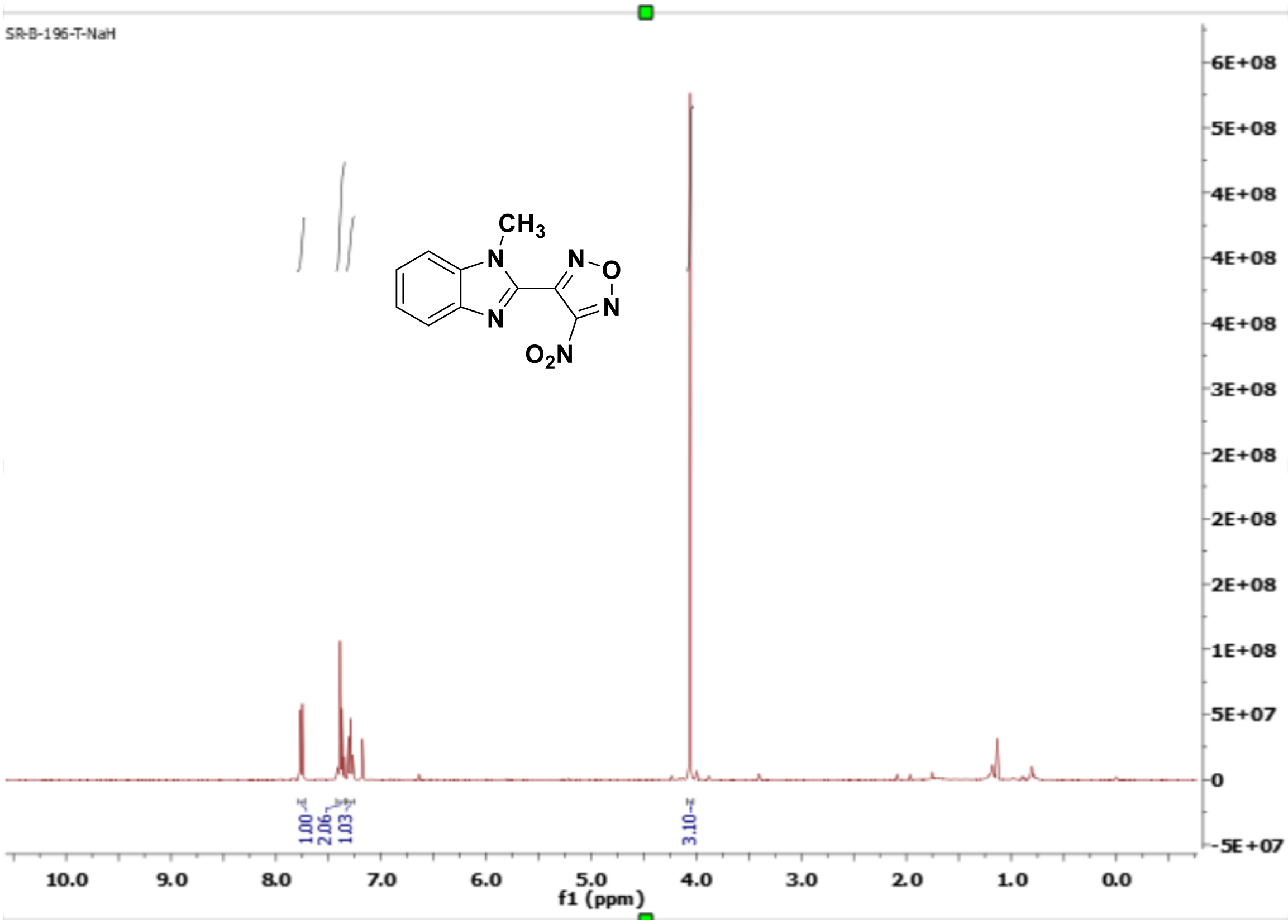

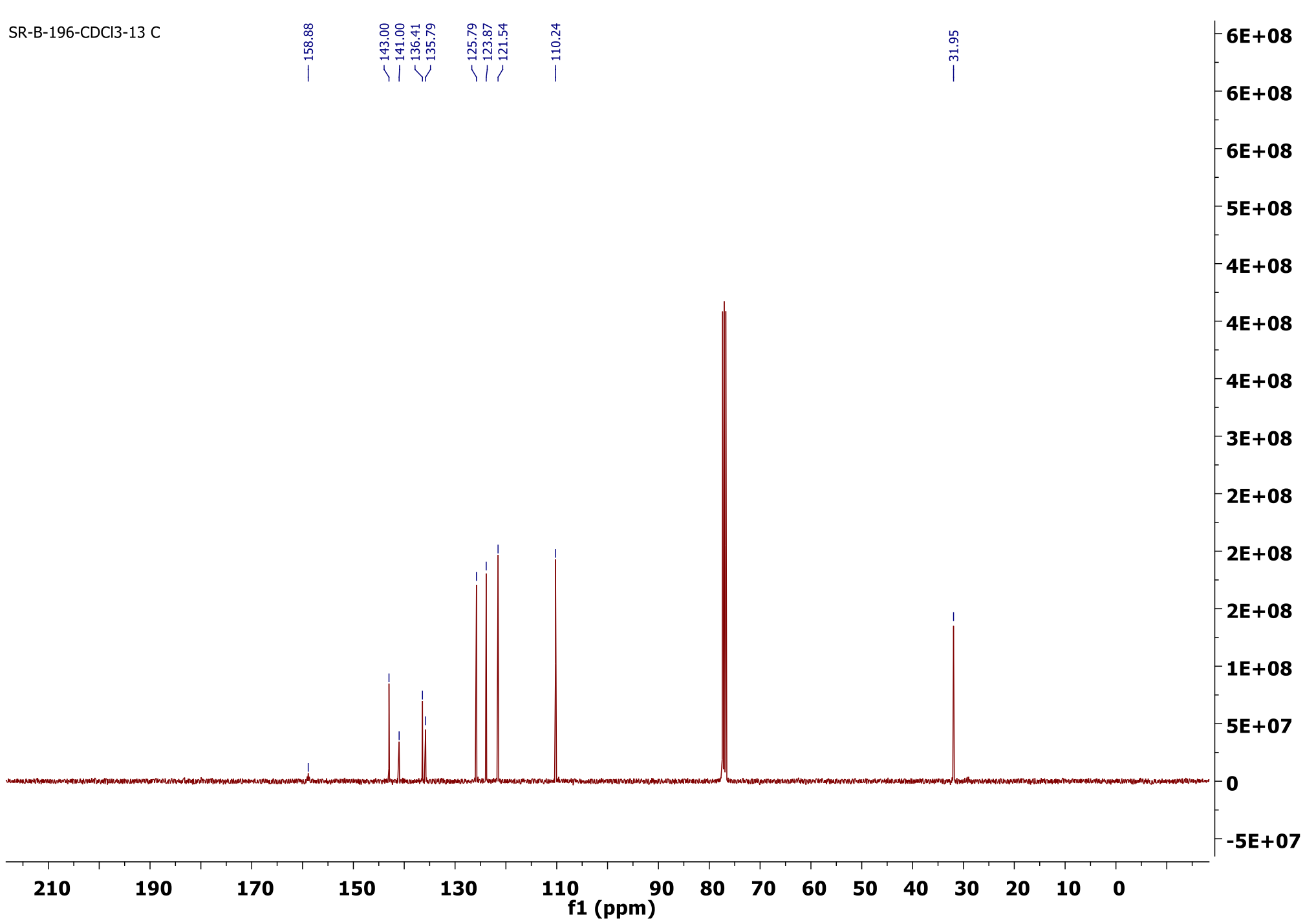

Figure S18. ^{1}H NMR and ^{13}C NMR spectra of SR-B-196 in $CDCl_3$

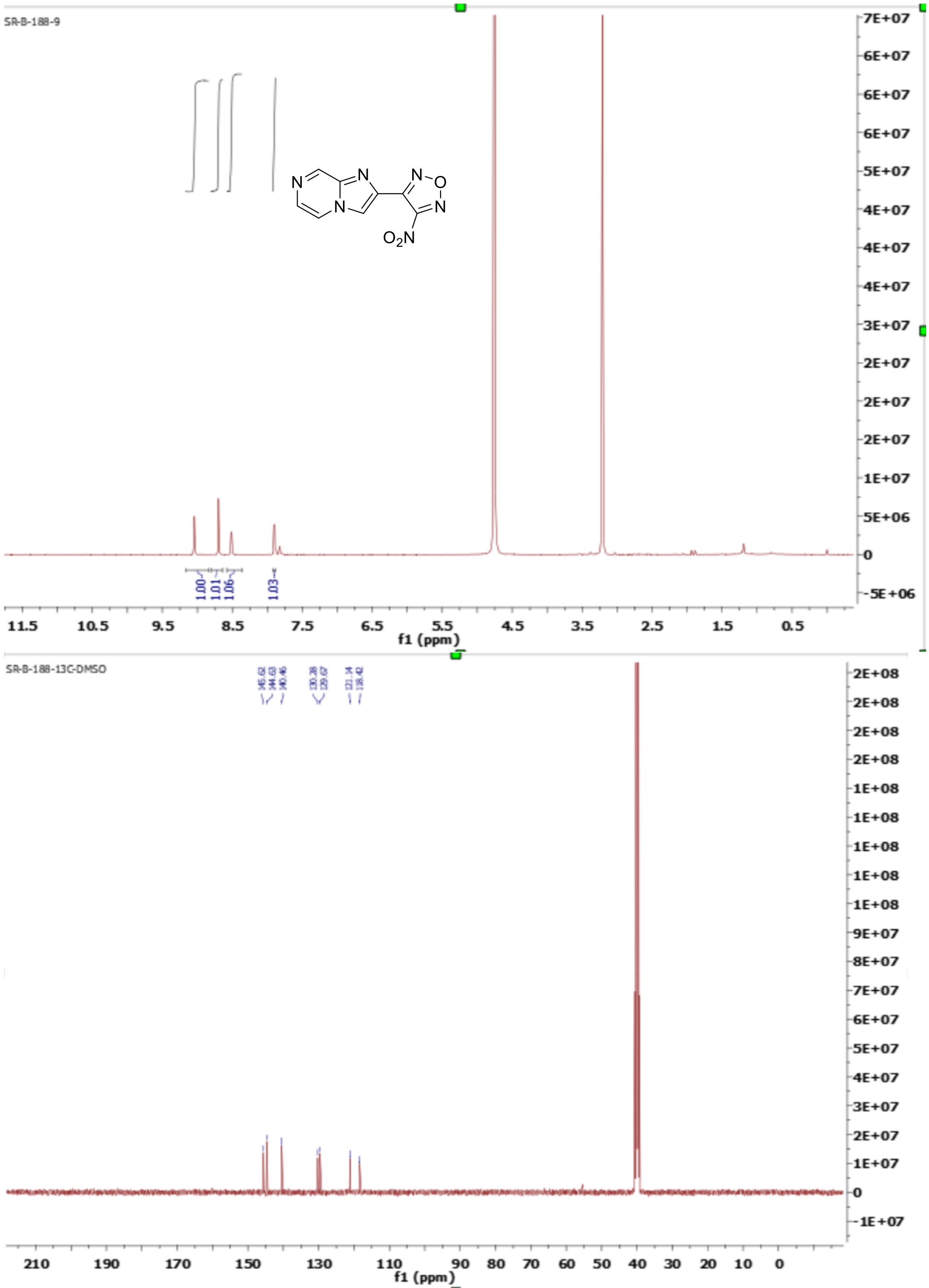

Figure S19. ¹H NMR and ¹³C NMR spectra of SR-B-188 in CD_3OD

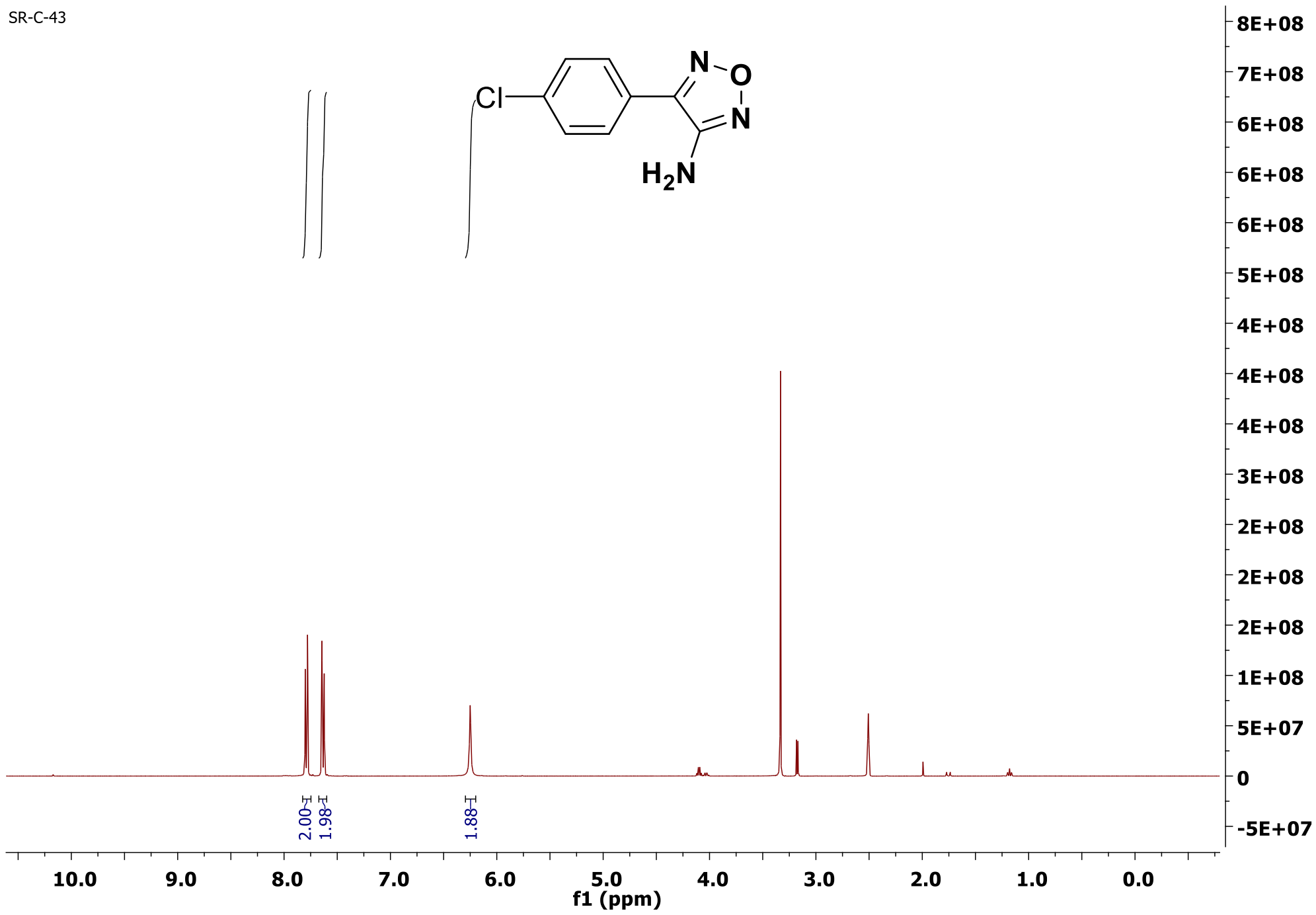

Figure S20. ^{1}H NMR spectra of 5a in DMSO-d_6

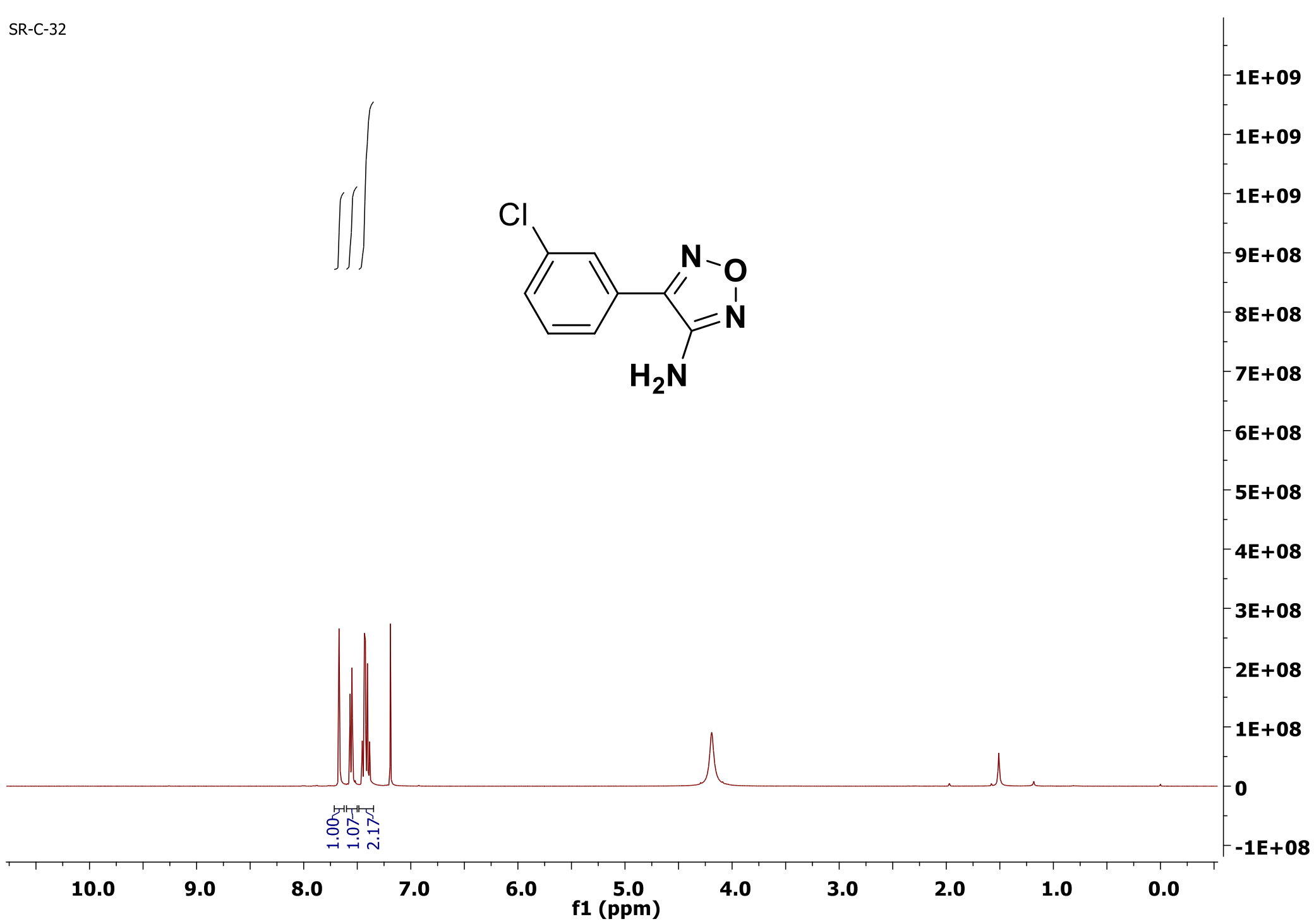

Figure S21. ^{1}H NMR spectra of 5b in $CDCl_3$

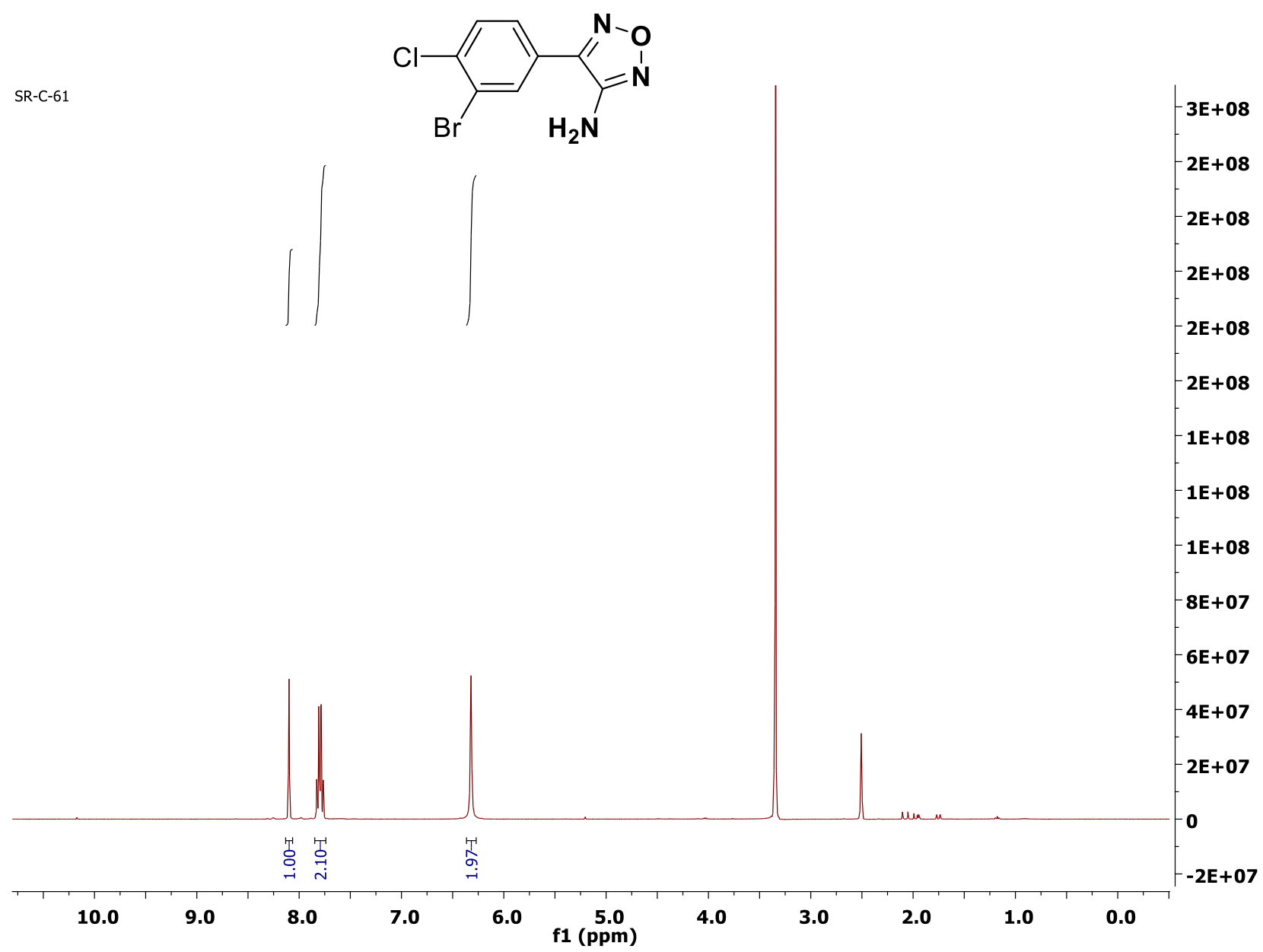

Figure S22. ^{1}H NMR spectra of 5c in DMSO-d_6

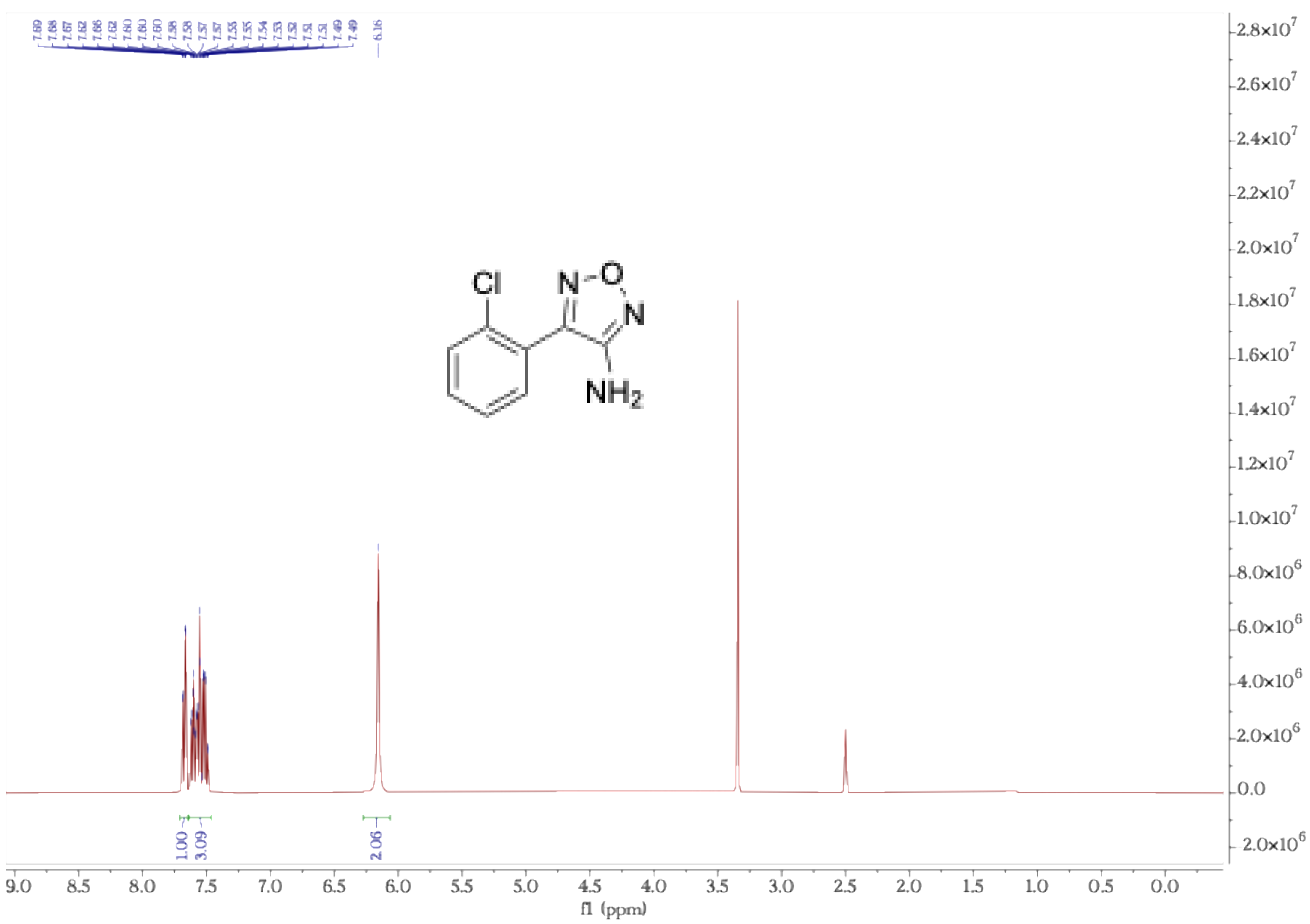

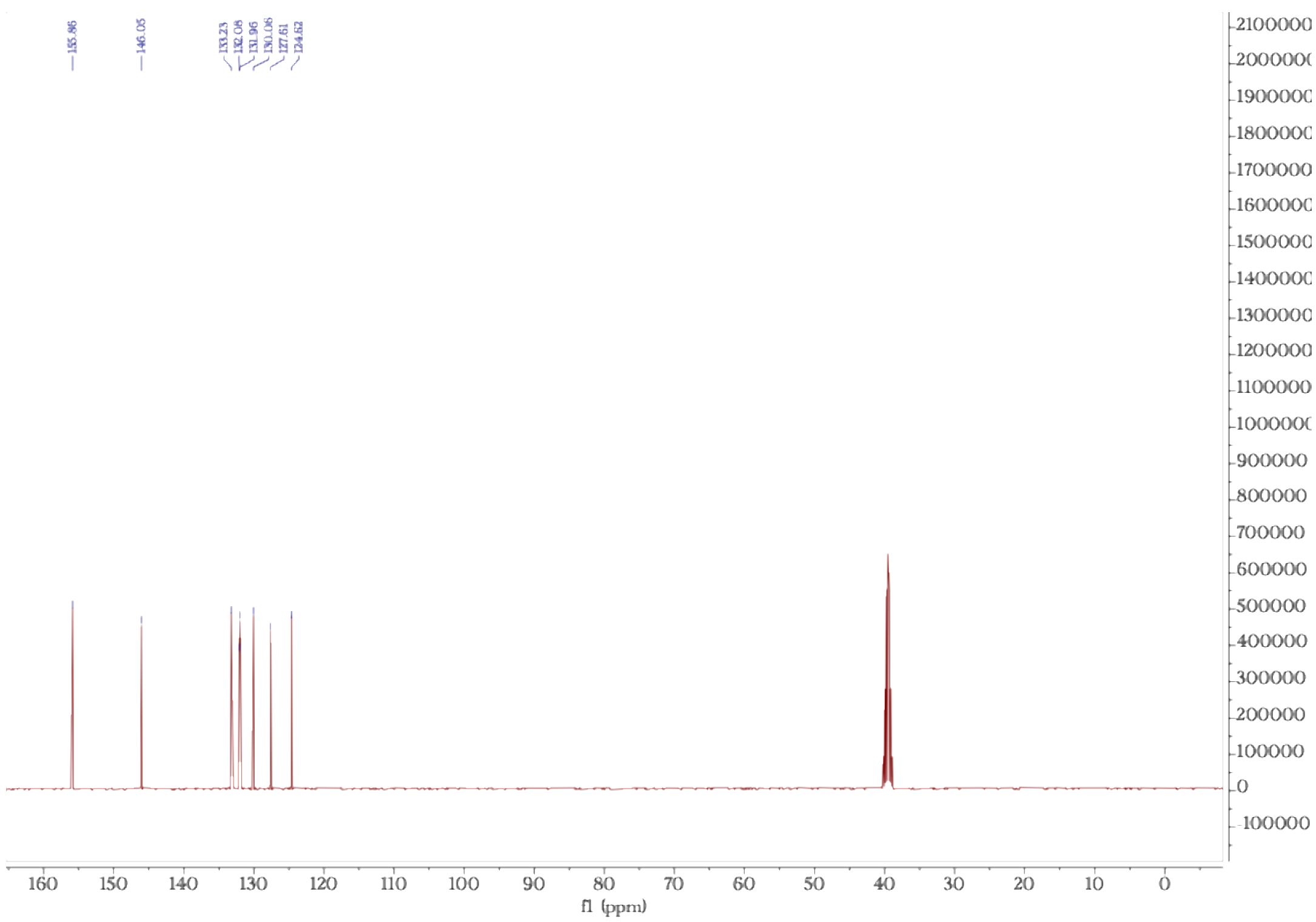

Figure S23. ^{1}H NMR and ^{13}C NMR spectra of 5d in DMSO-d_6

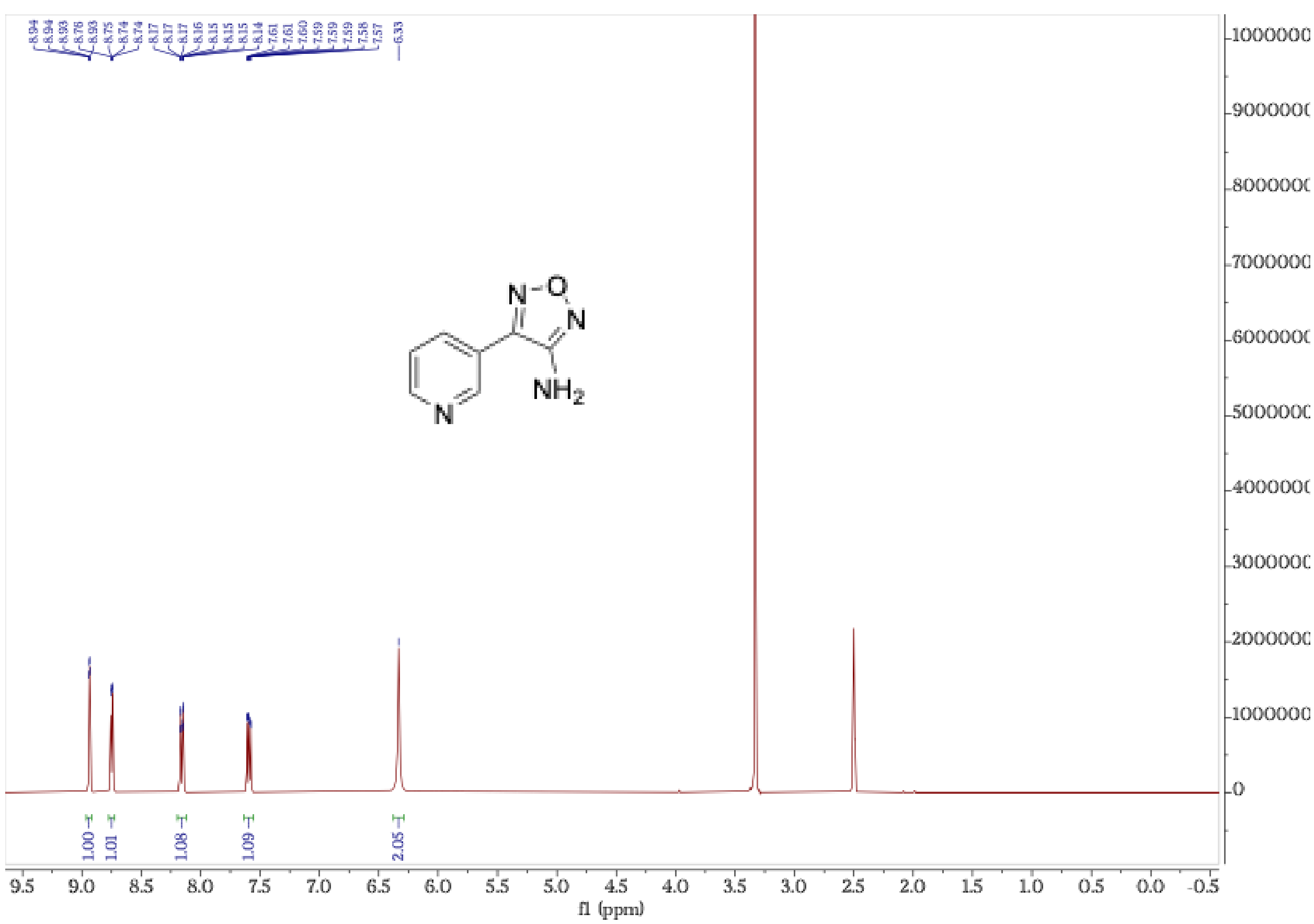

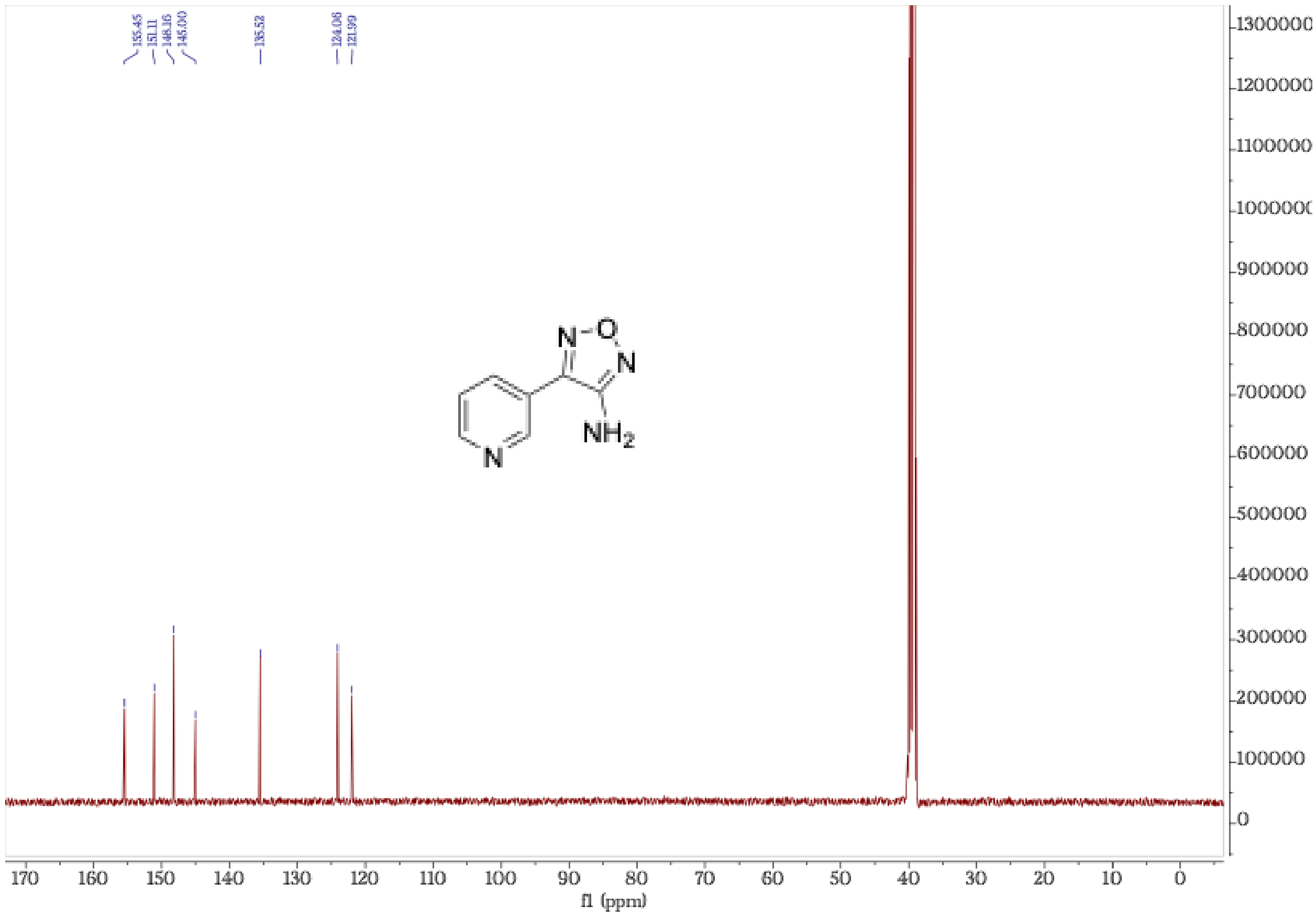

Figure S24. **^{1}H NMR and ^{13}C NMR spectra of 5e in DMSO-d_6**

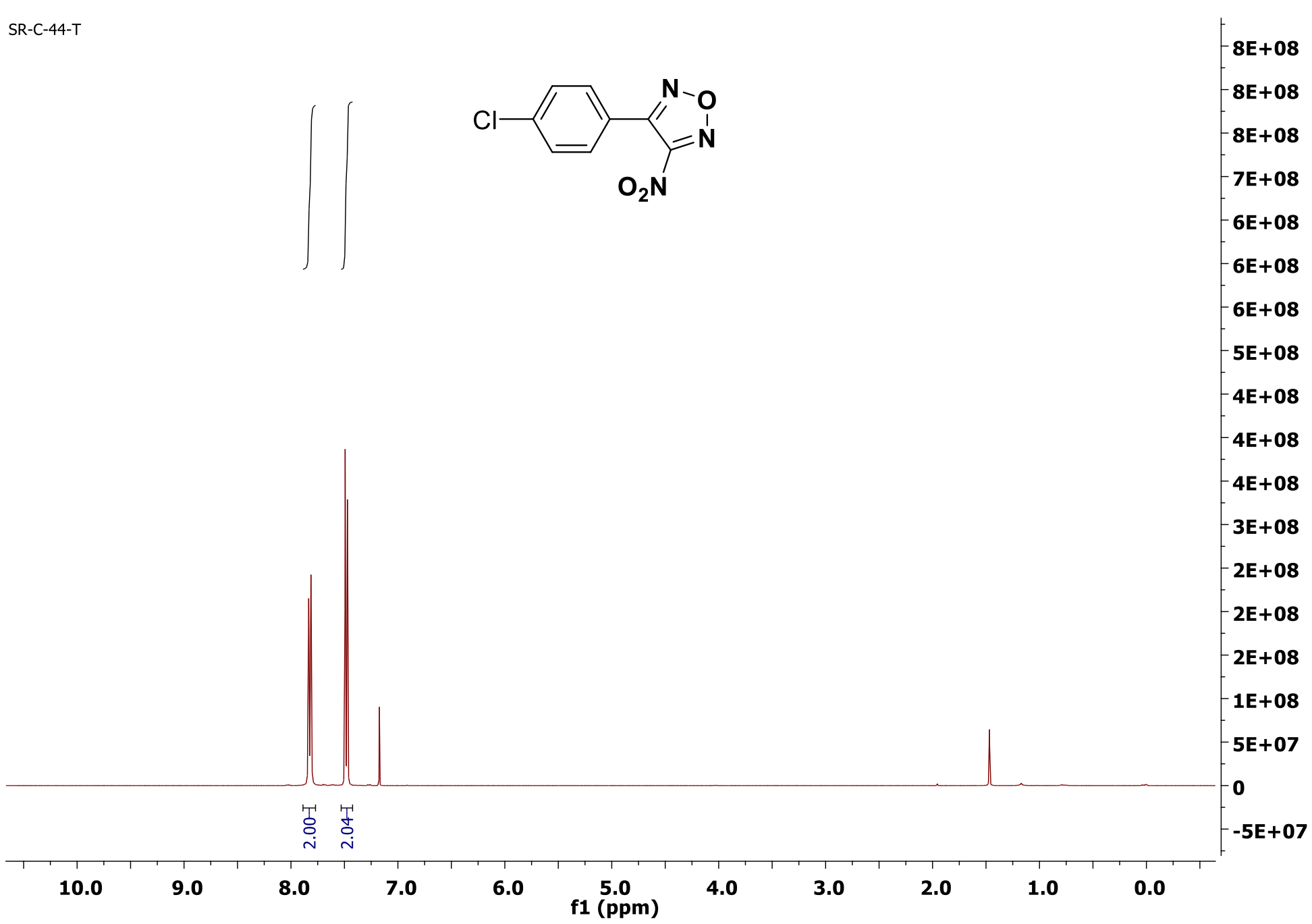

Figure S25. **^{1}H NMR spectra of SR-C-44 in $CDCl_3$**

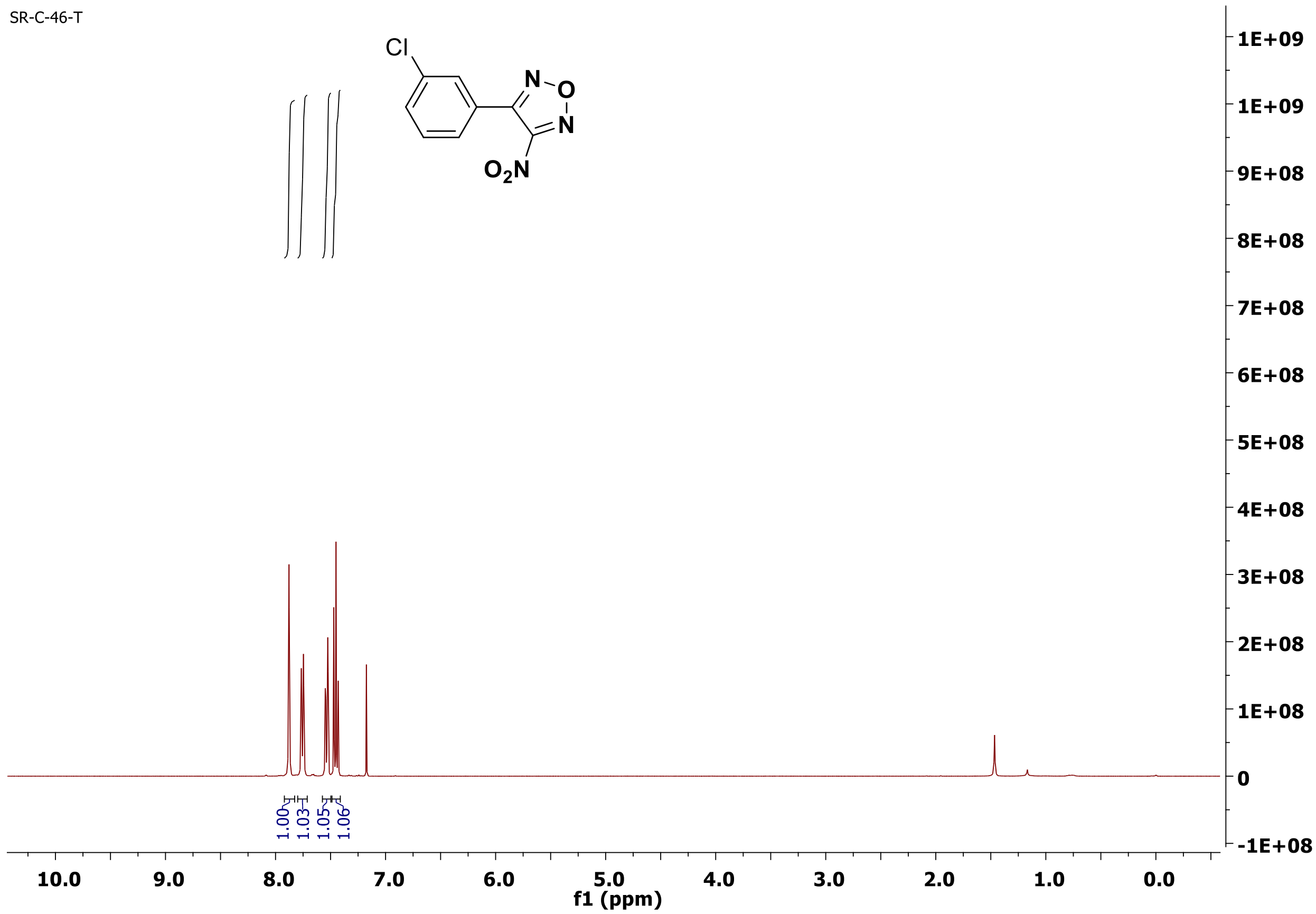

Figure S26. ^{1}**H NMR spectra of SR-C-46 in** $CDCl_3$

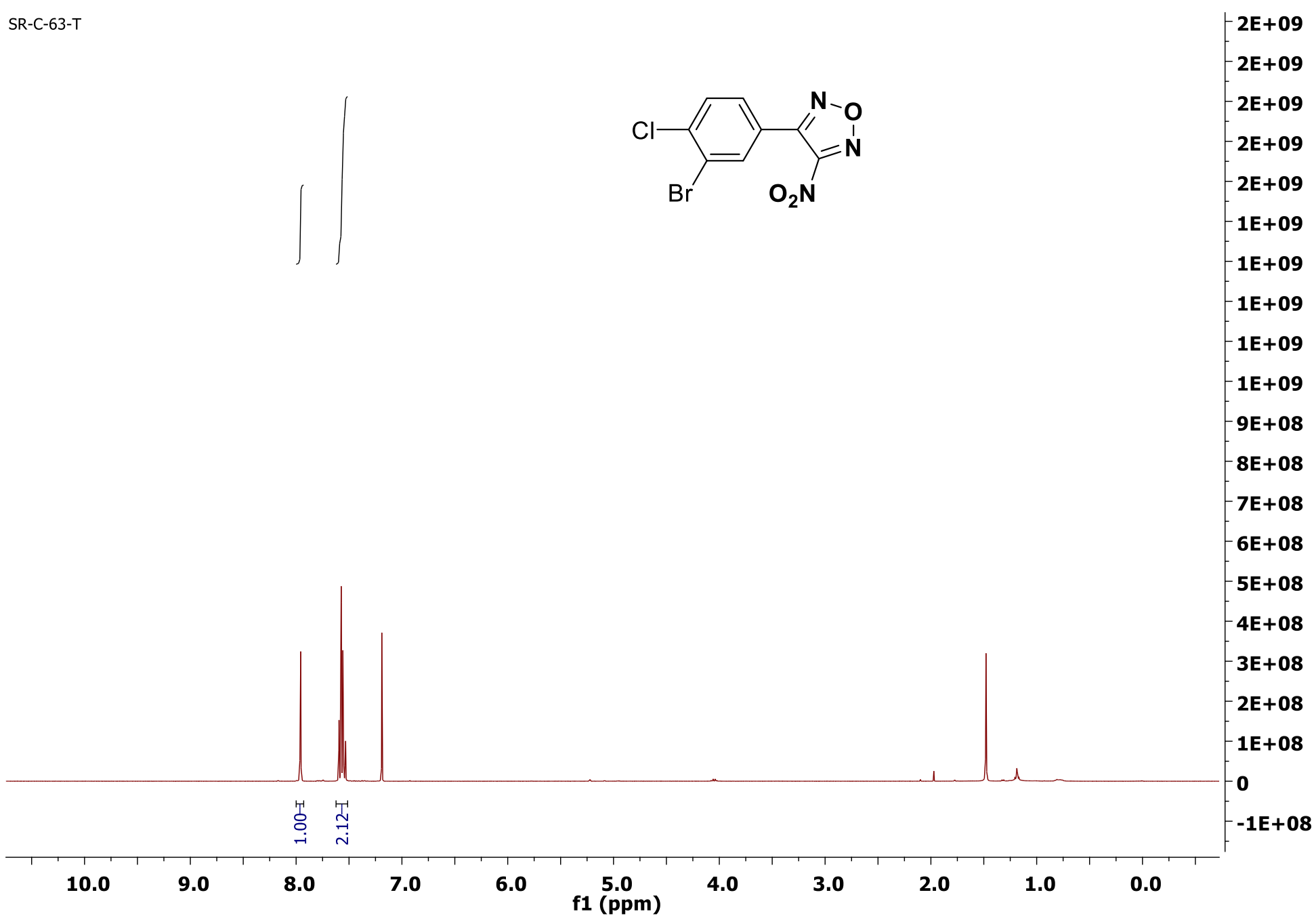

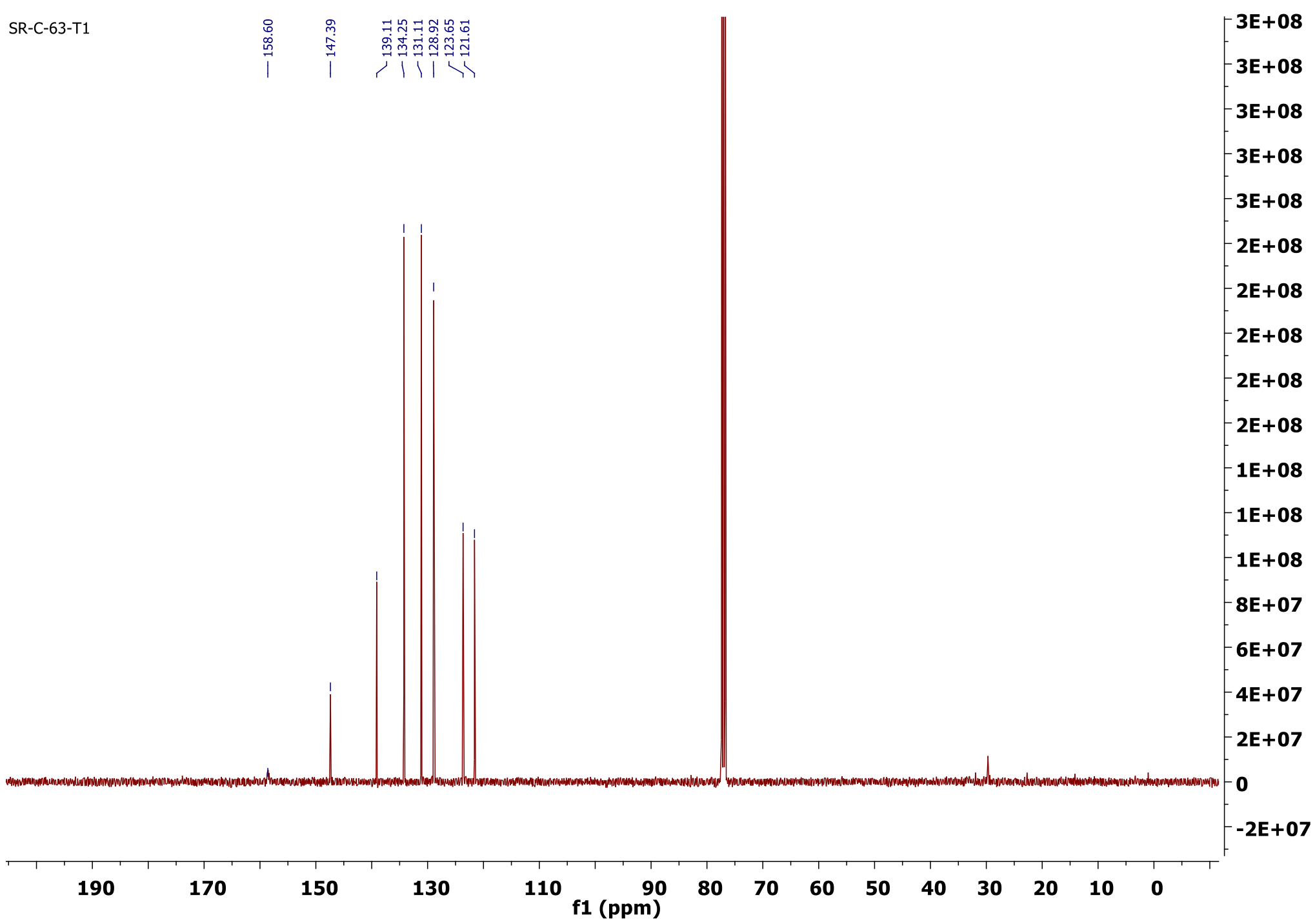

Figure S27. ^{1}H **NMR and** ^{13}C **NMR spectra of SR-C-63 in** $CDCl_3$

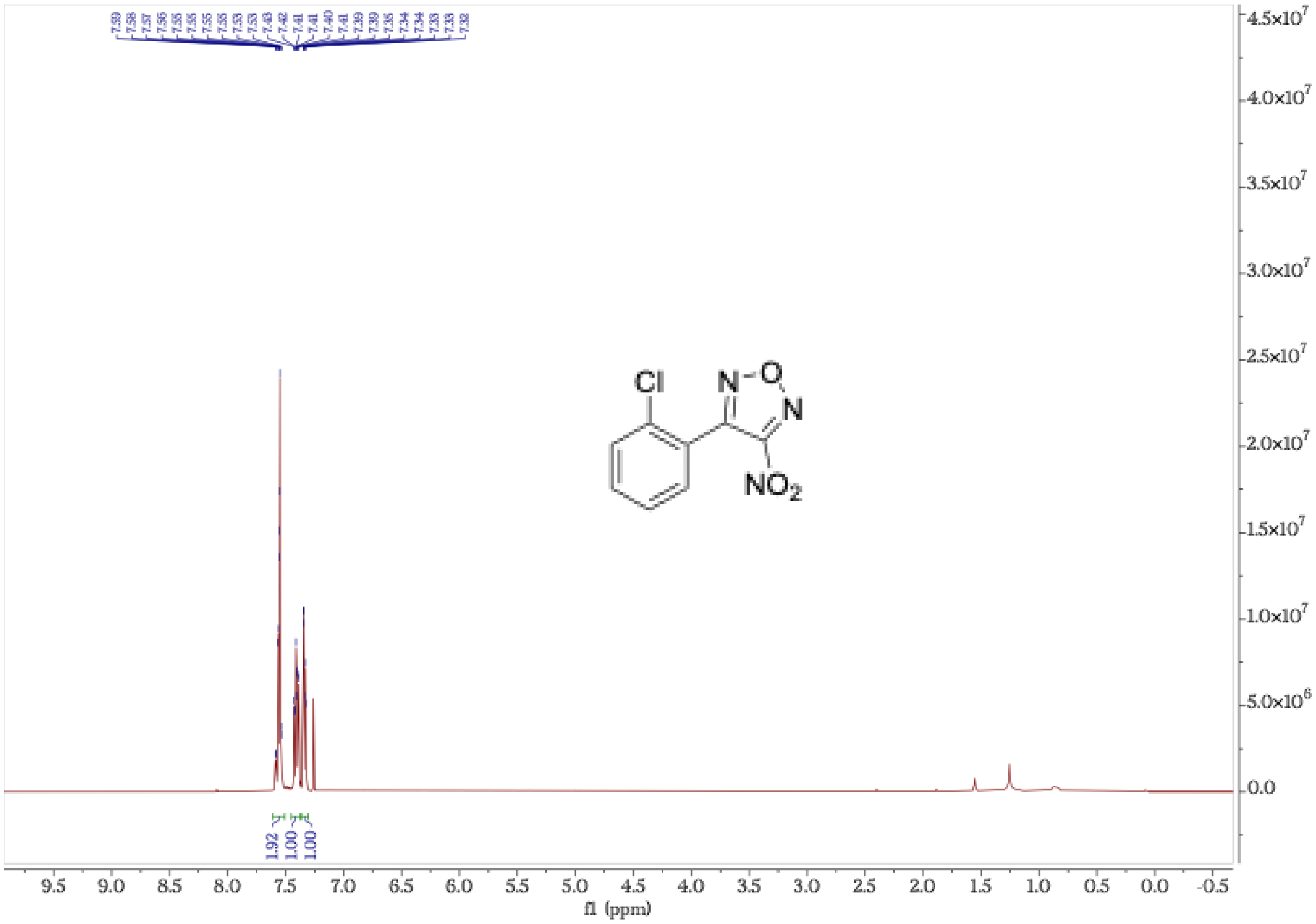

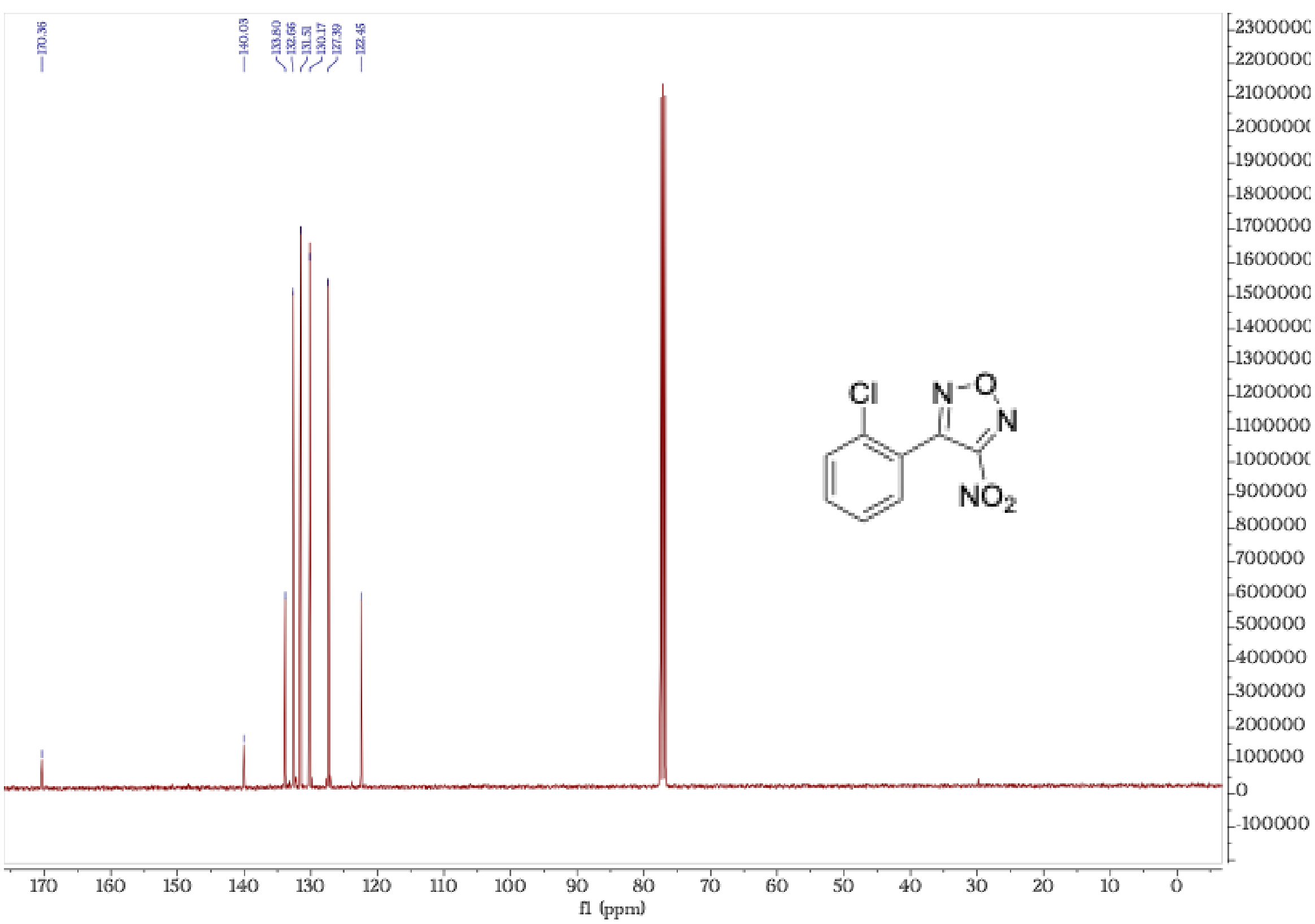

Figure S28. ^{1}H NMR and ^{13}C NMR spectra of NS-A-135 in $CDCl_3$

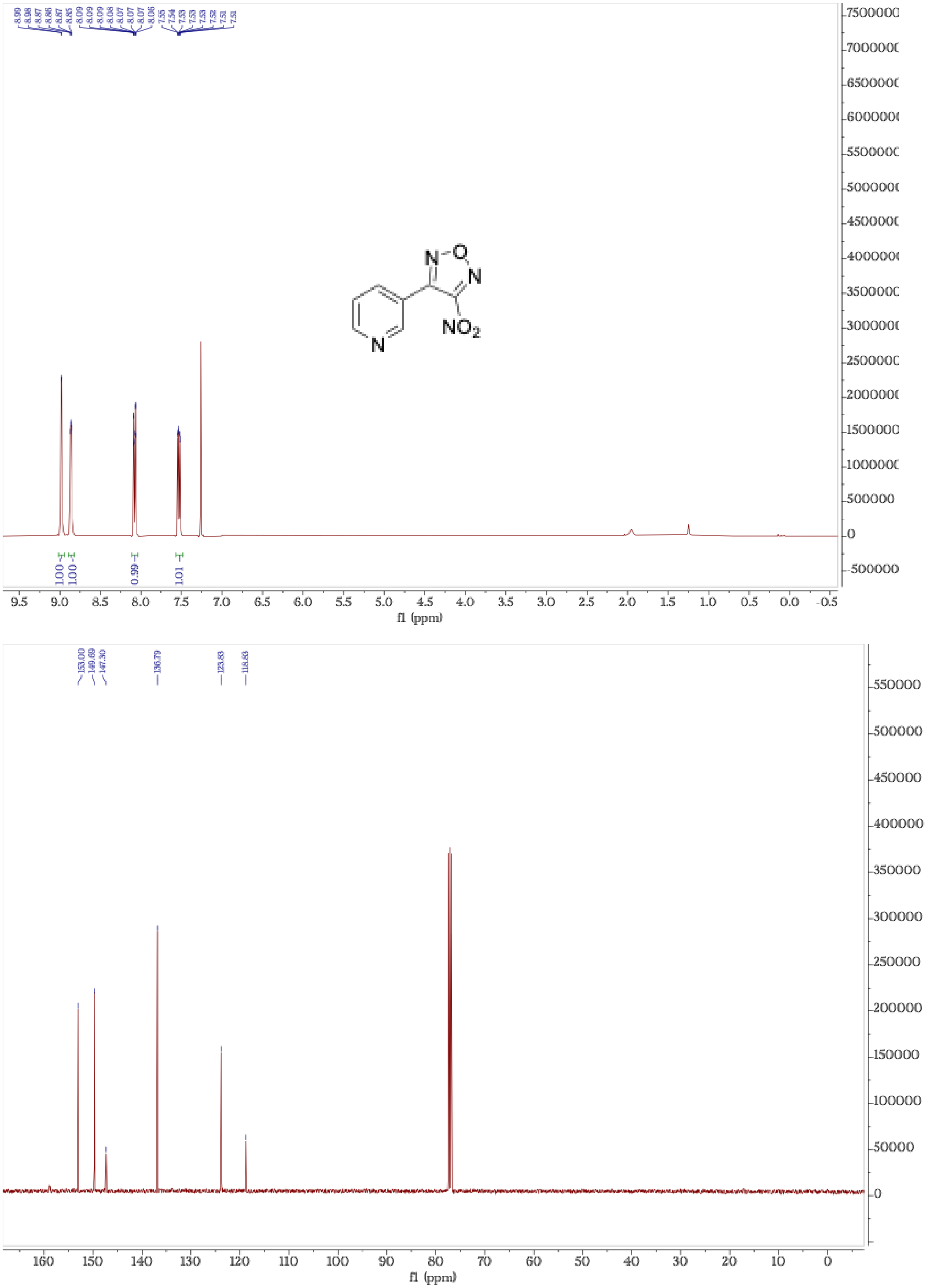

Figure S29. ^{1}H NMR and ^{13}C NMR spectra of NS-A-148 in $CDCl_3$

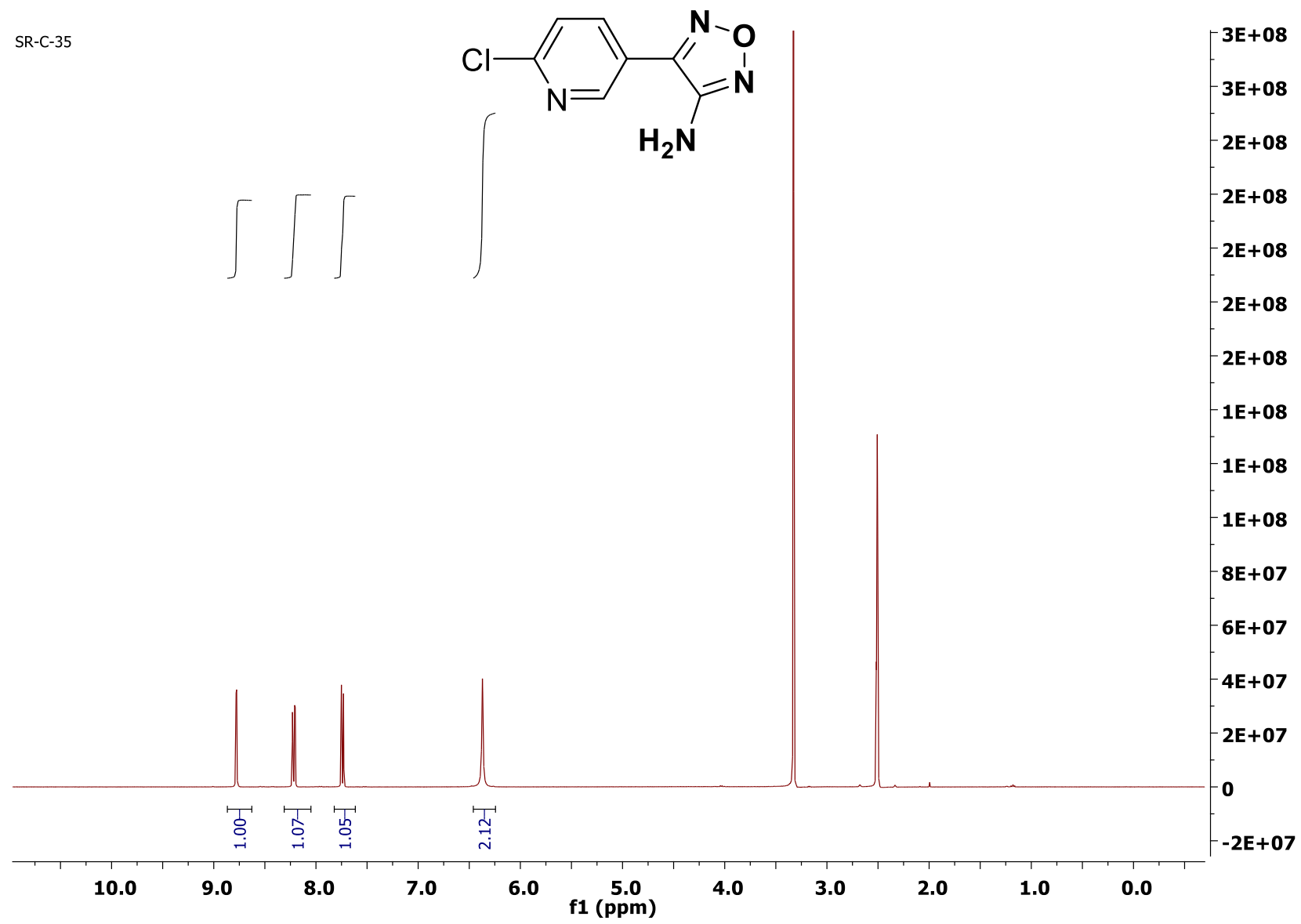

Figure S30. ^{1}H NMR spectra of SR-C-38 in DMSO-d_6

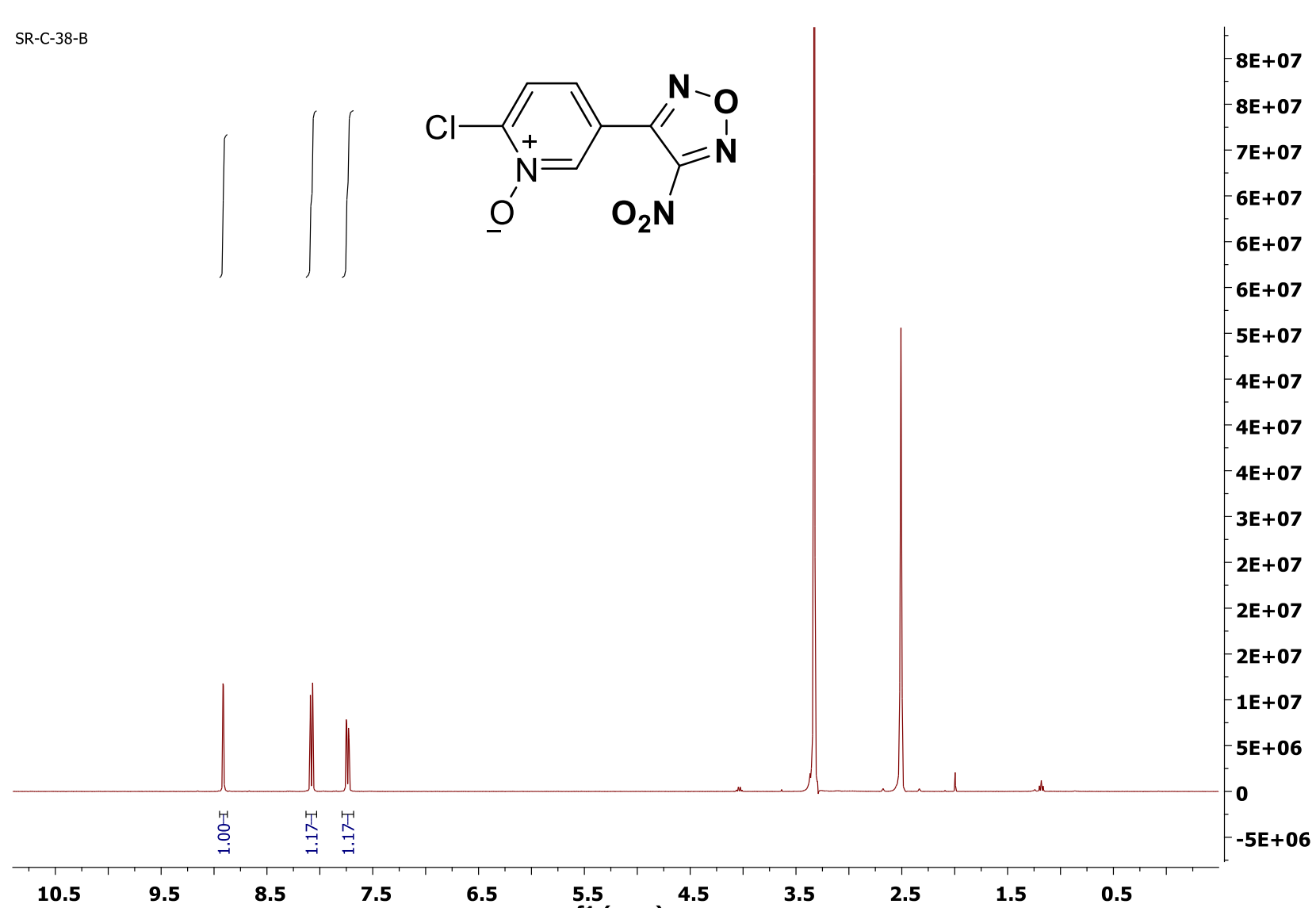

Figure S31. ^{1}H NMR spectra of SR-C-38 in DMSO-d_6

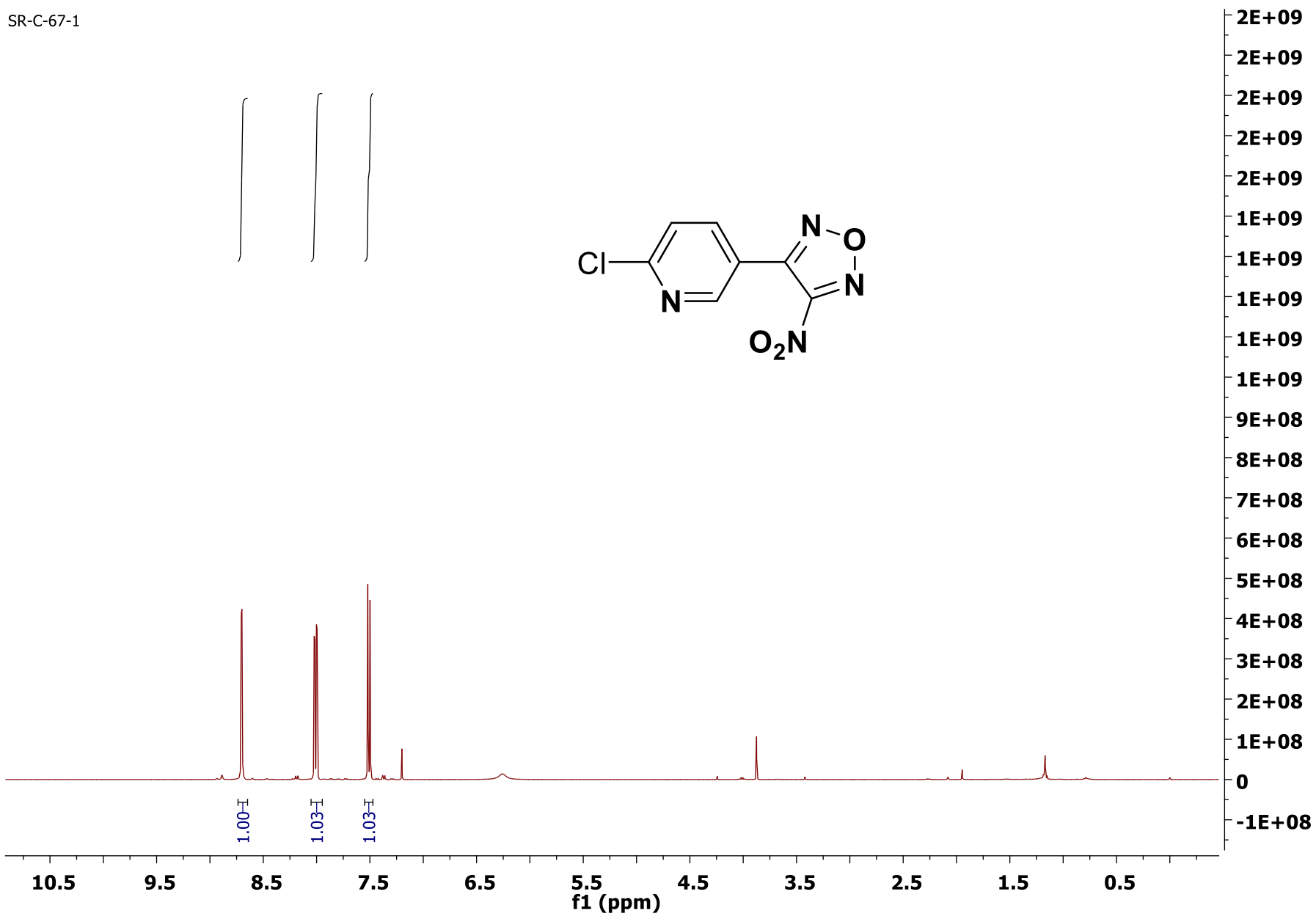

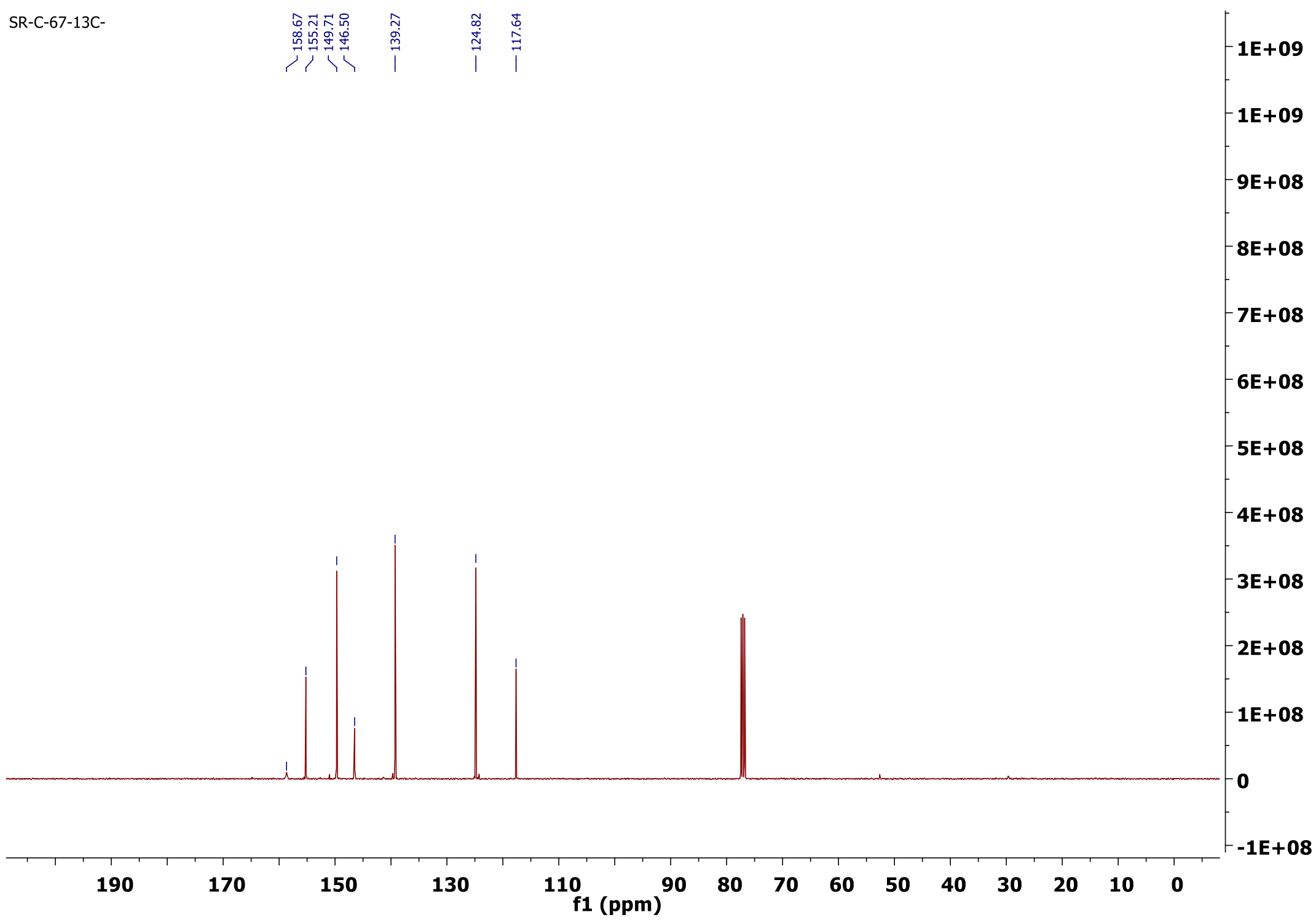

Figure S32. ^1H NMR and ^{13}C NMR spectra of SR-C-67 in $CDCl_3$

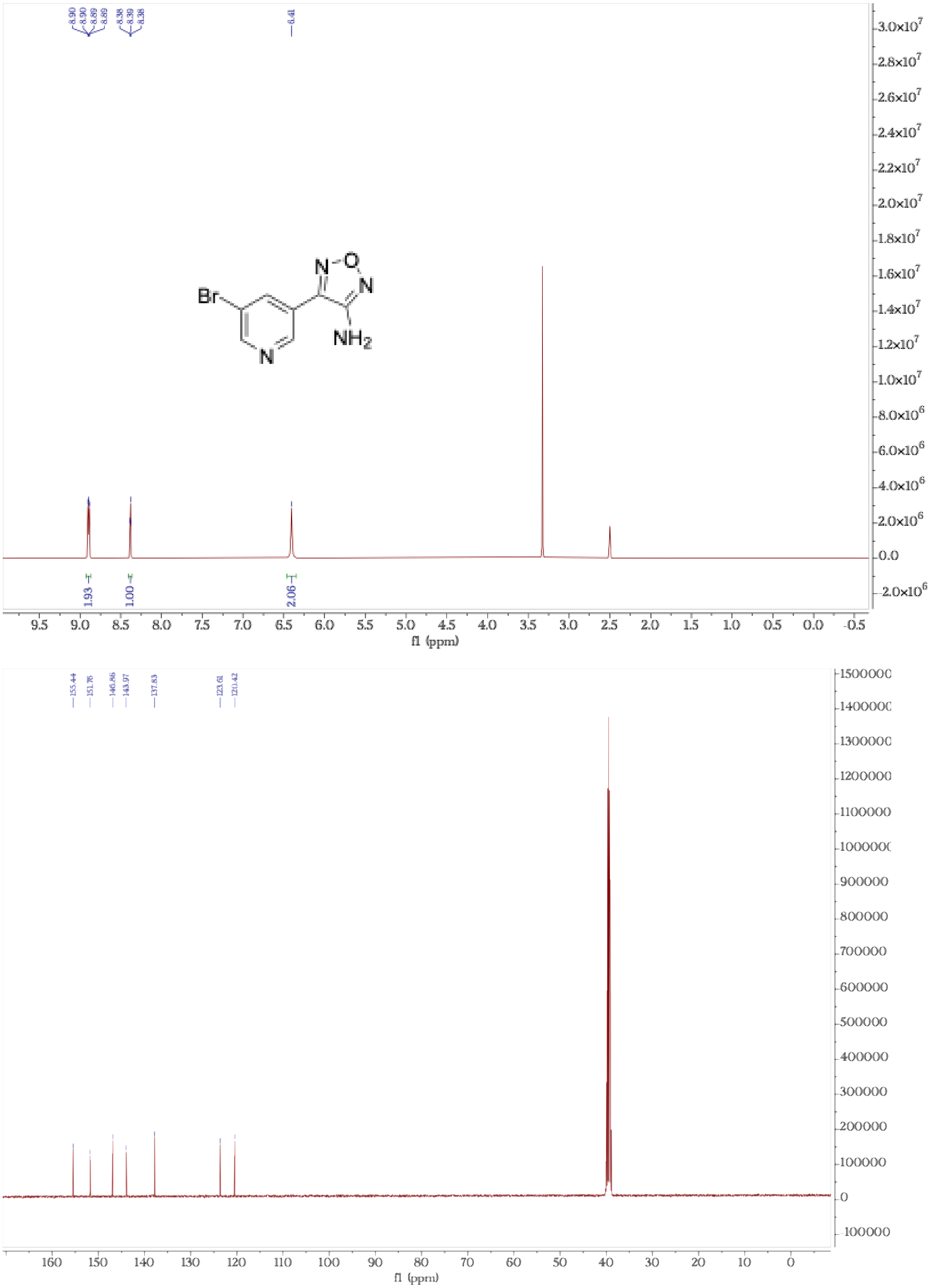

Figure S33. ^{1}H **NMR and** ^{13}C **NMR spectra of 13 in DMSO-*d*₆**

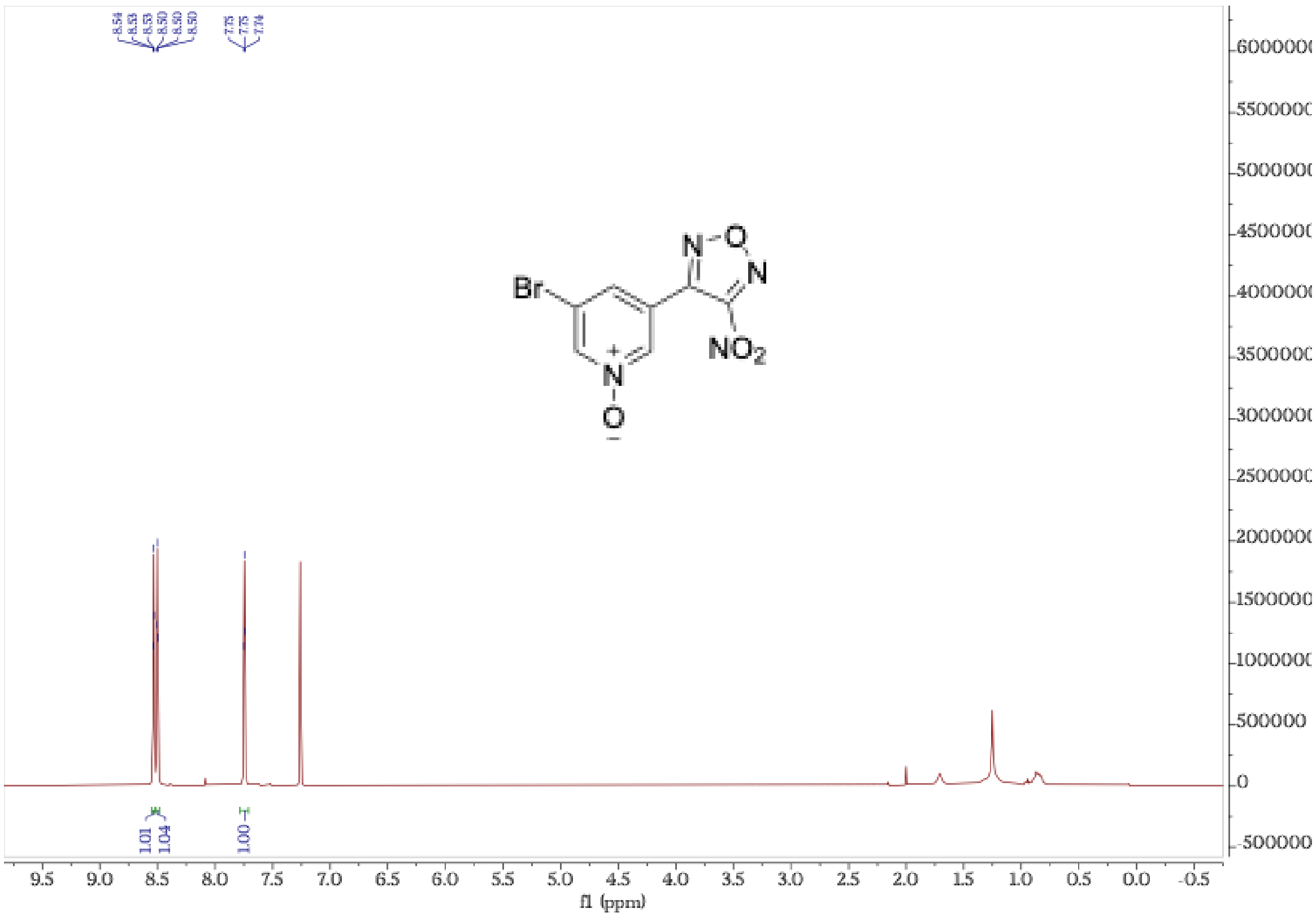

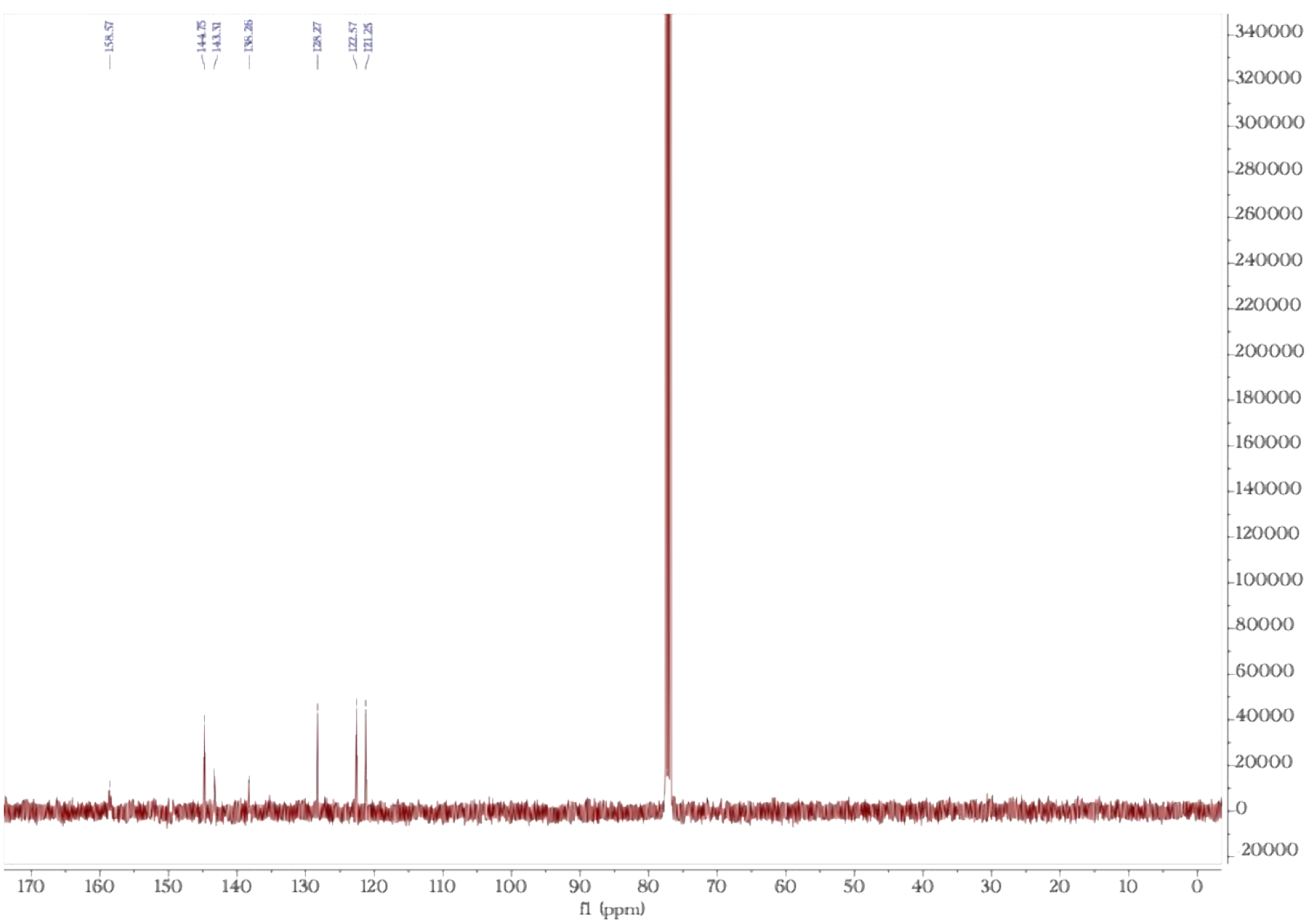

Figure S34. **^{1}H NMR and ^{13}C NMR spectra of NS-A-136 in $CDCl_3$**

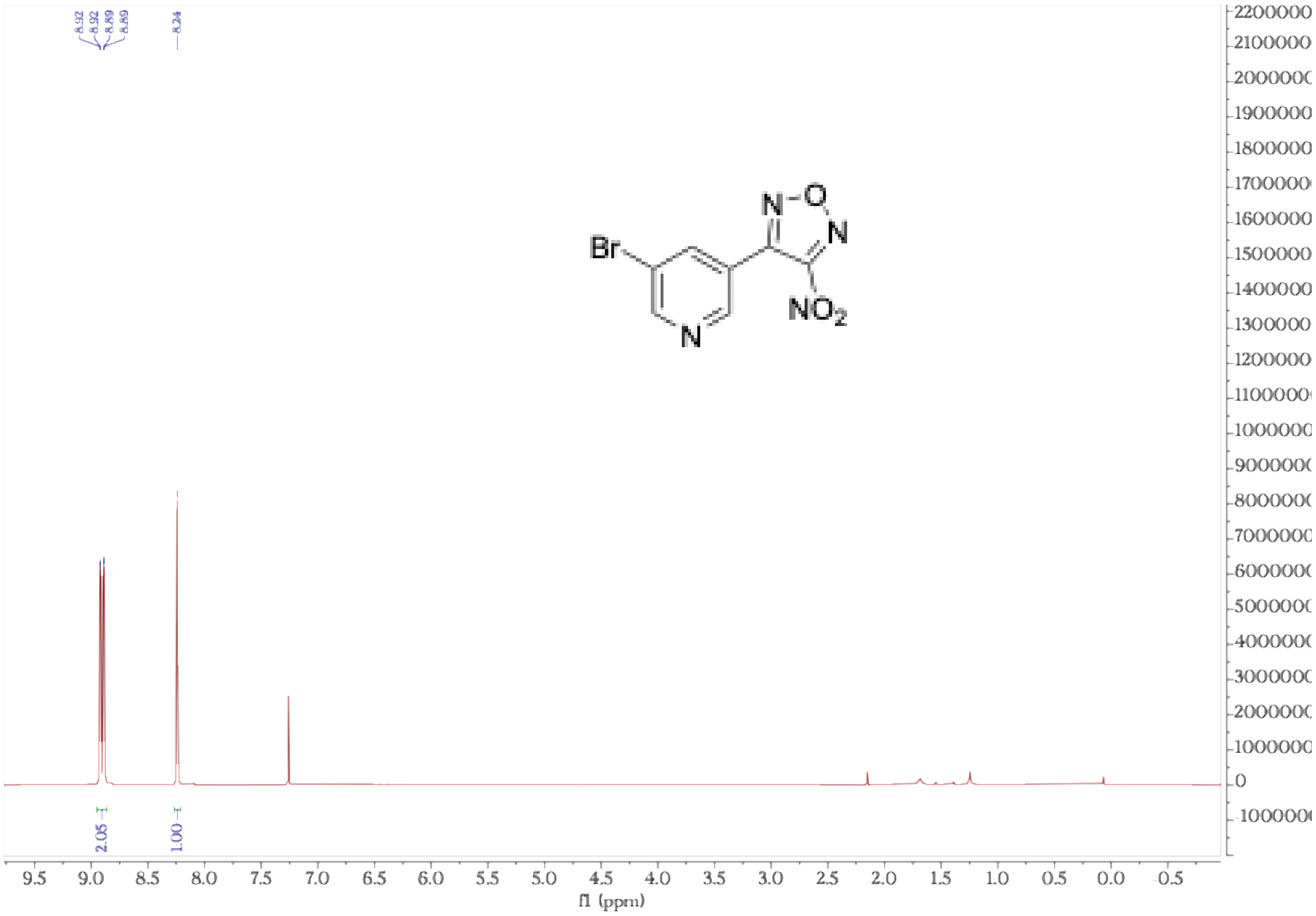

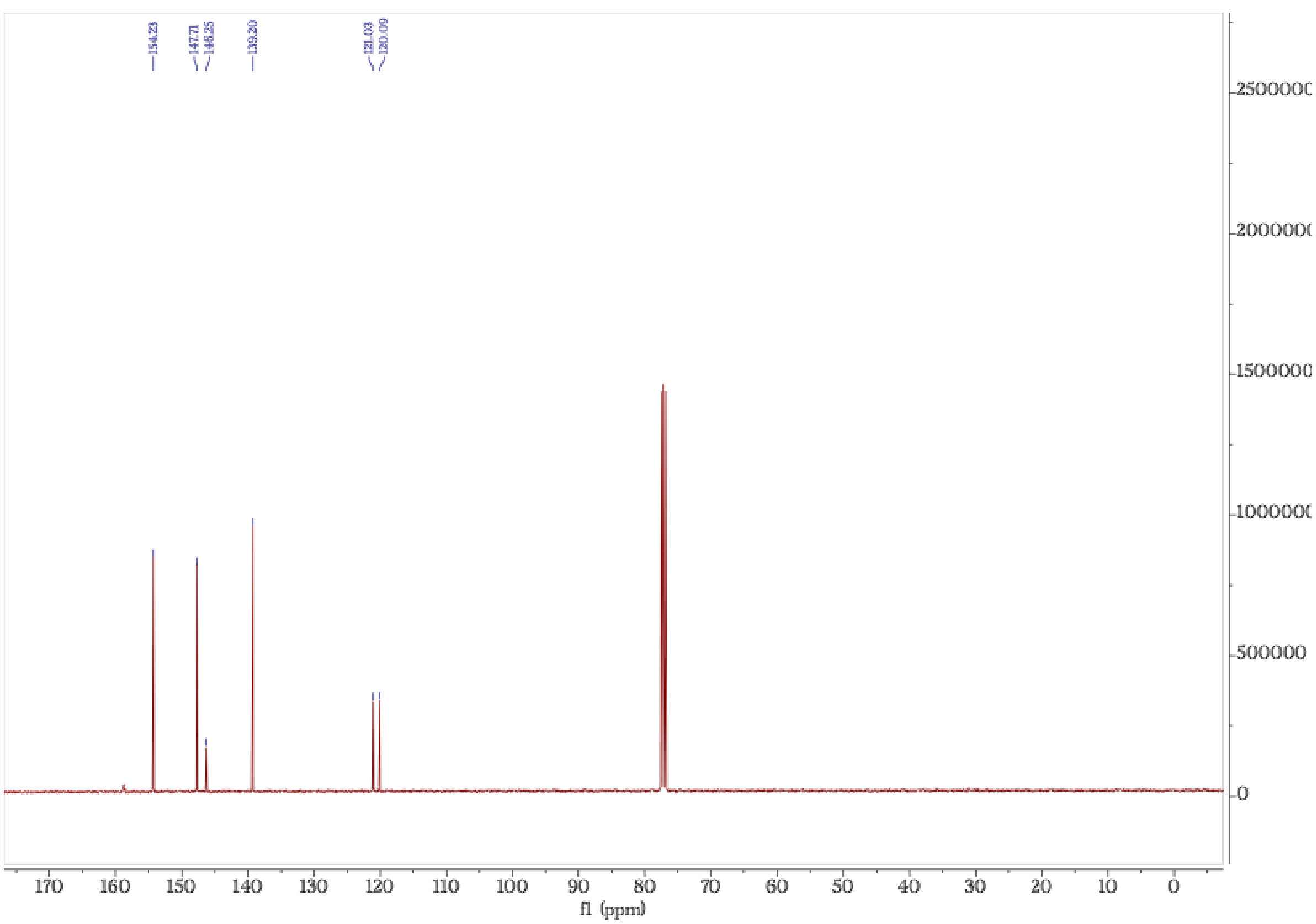

Figure S35. ^{1}H **NMR and** ^{13}C **NMR spectra of NS-A-141 in** $CDCl_3$

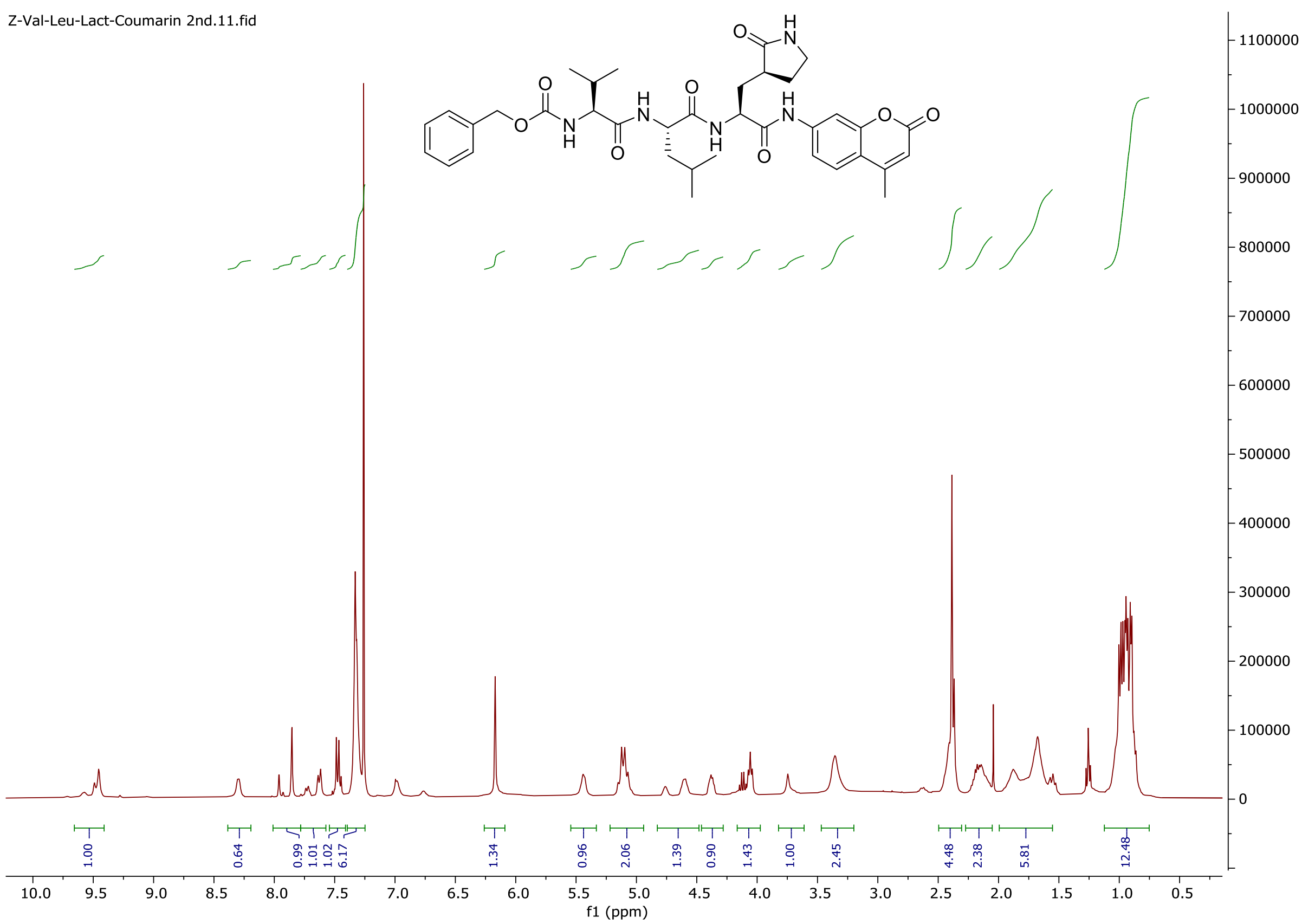

Figure S36. ^{1}H NMR spectra of Sub4 in $CDCl_3$

LC-MS Data of Final Compounds

SR-B-182

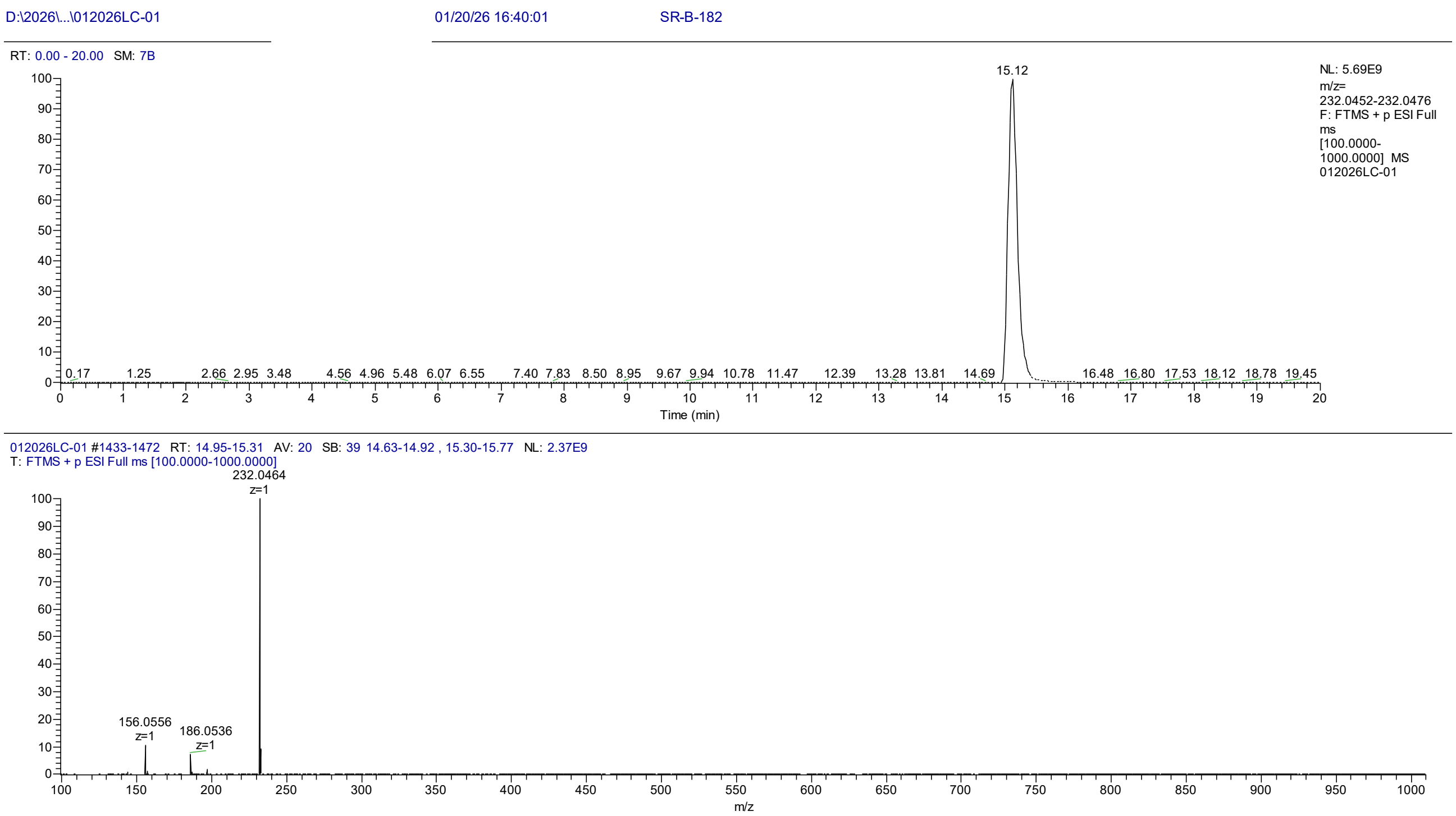

SR-C-67

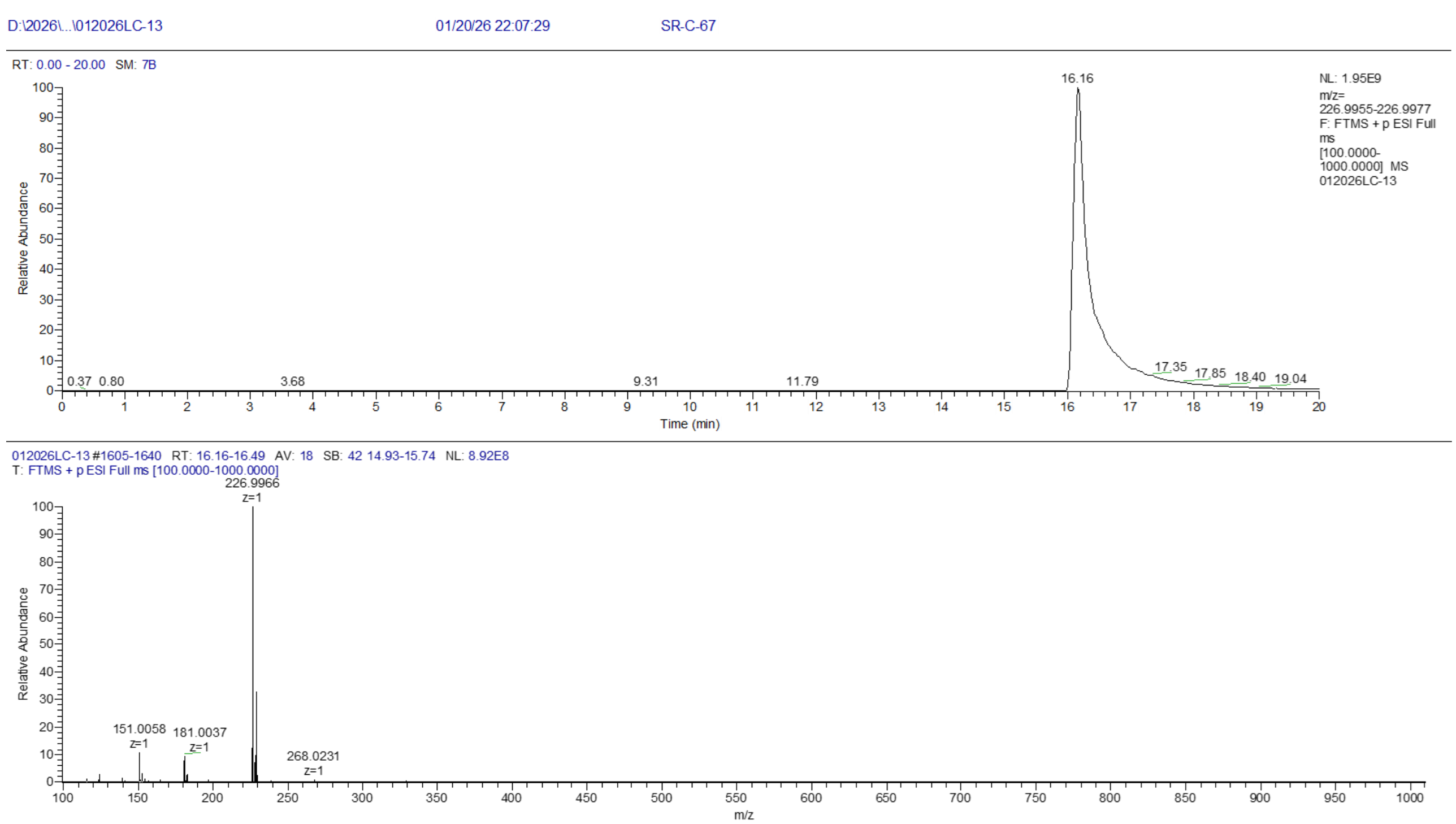

NS-A-148

D:\2026\...\012026LC-09 01/20/26 20:18:17 NS-A-148

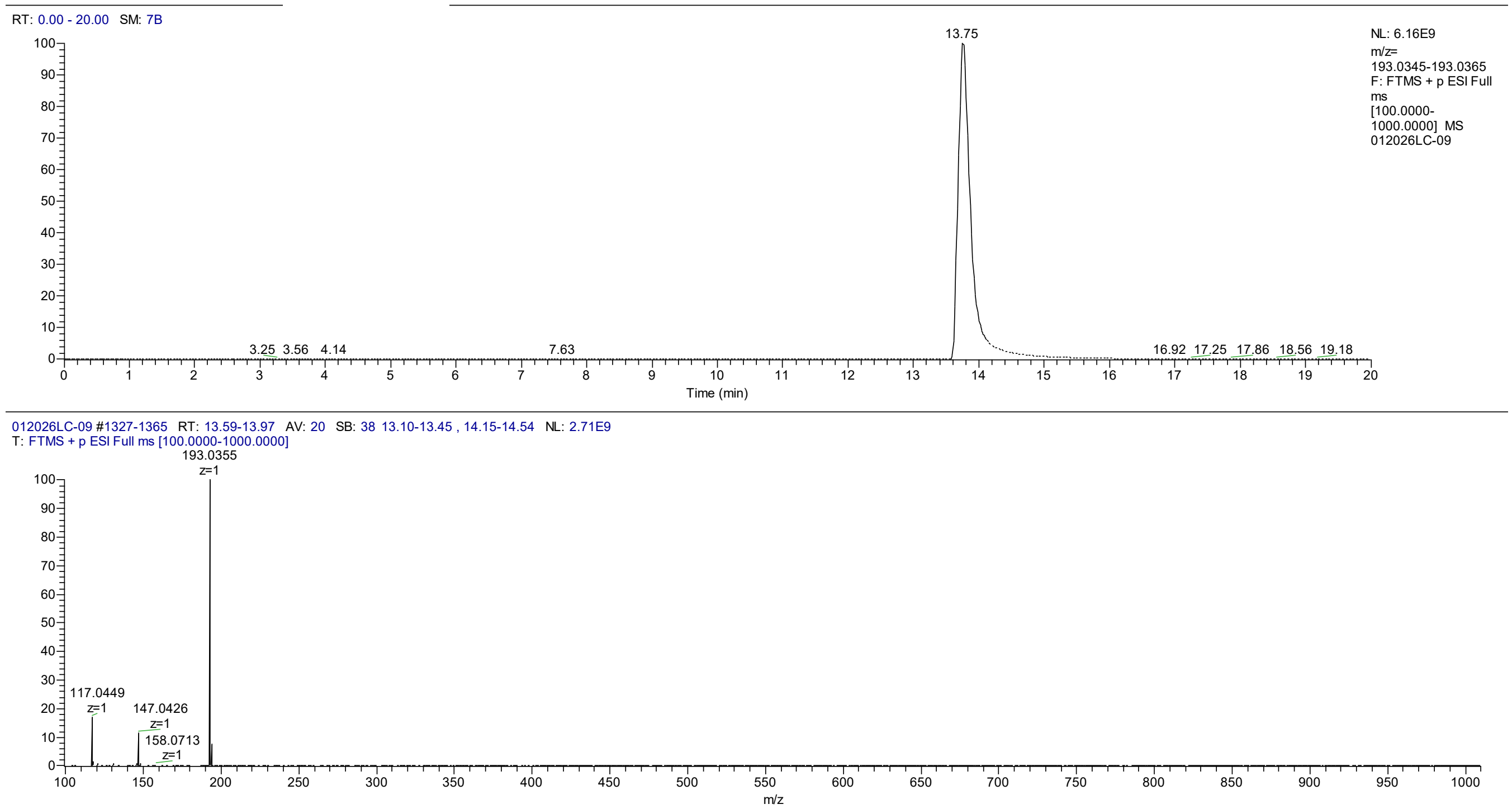

SR-B-196

D:\2026\...\012026LC-04 01/20/26 18:01:54 SR-B-196

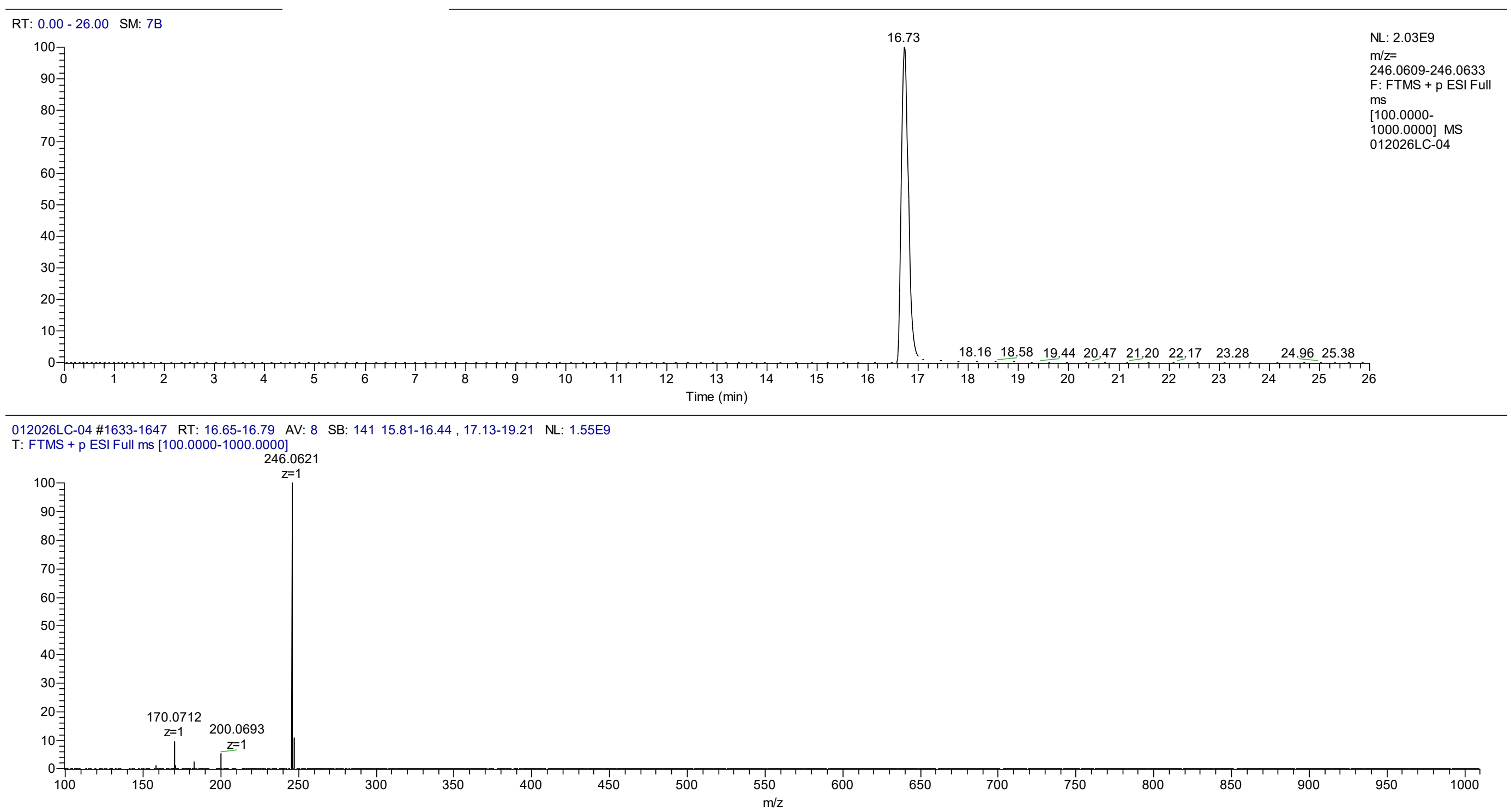

SR-C-38

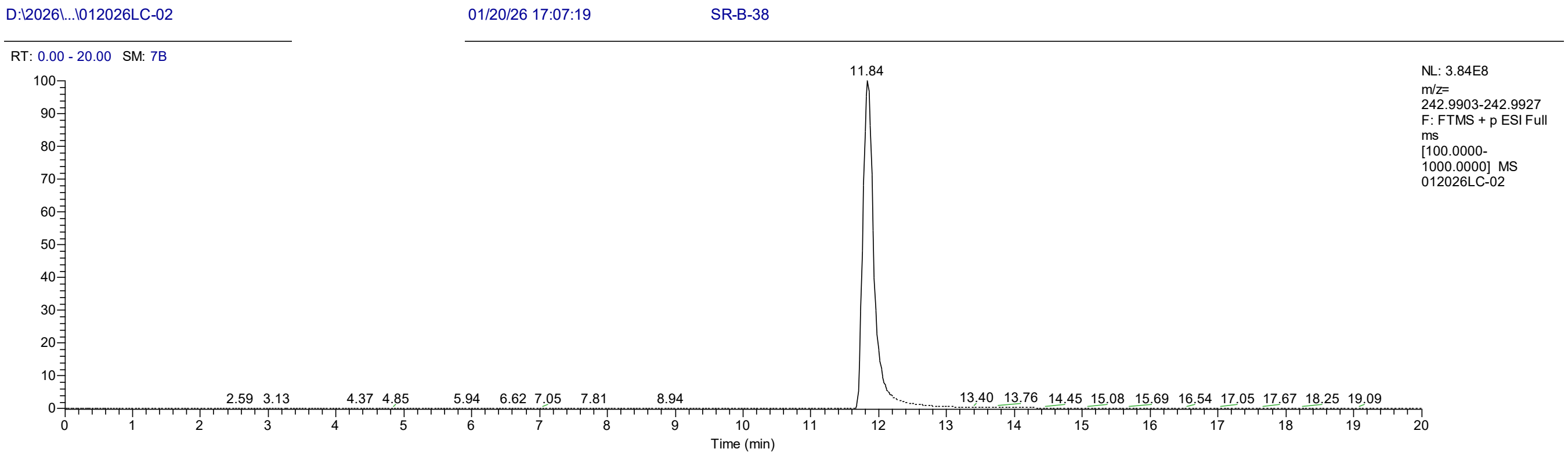

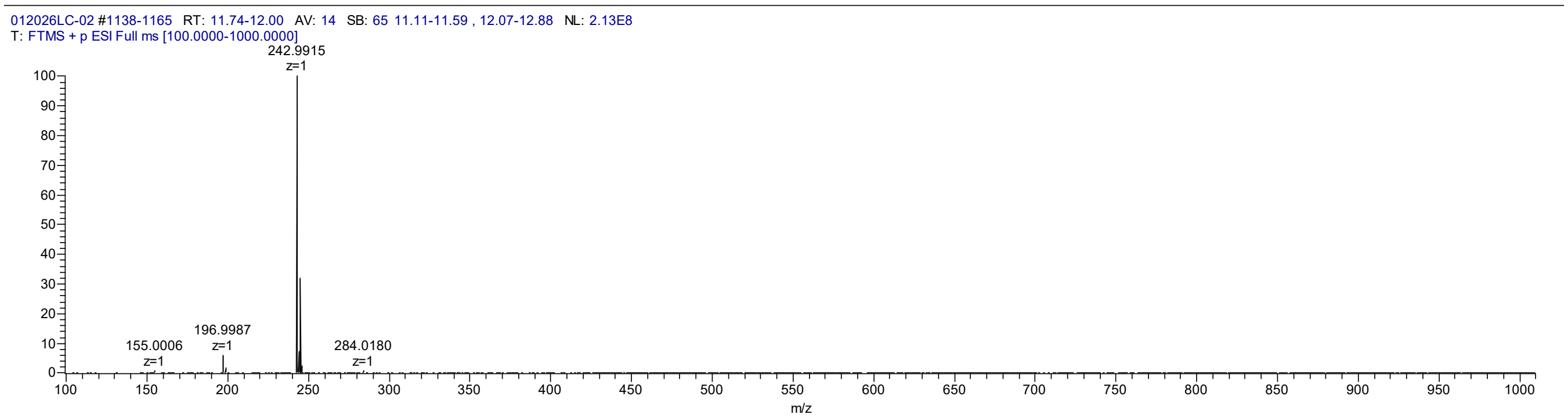

NS-A-141

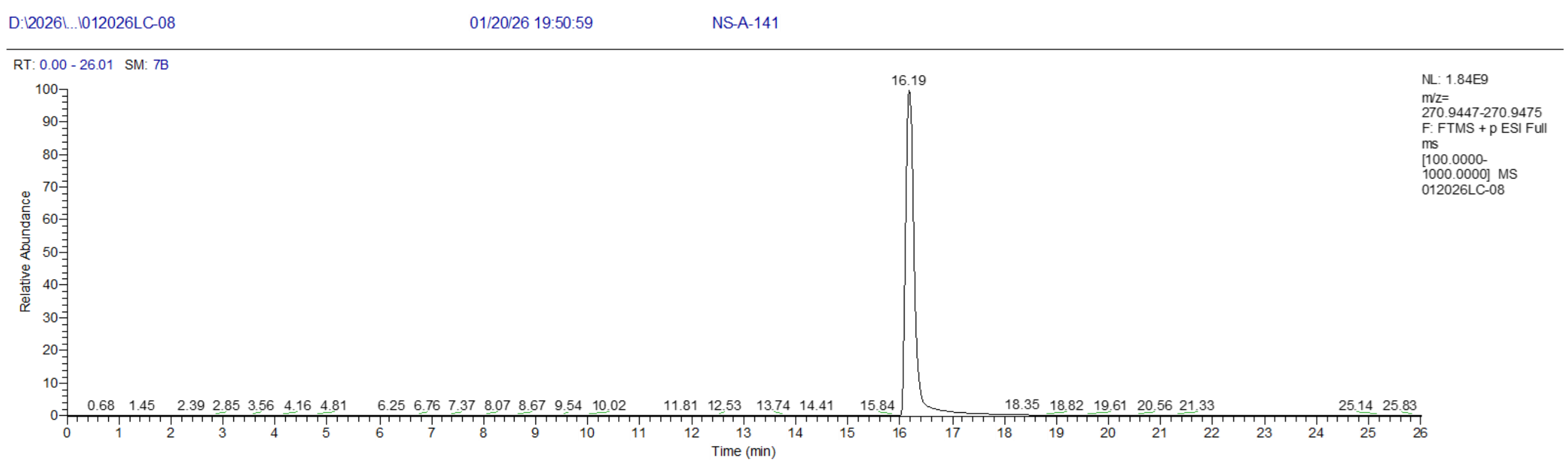

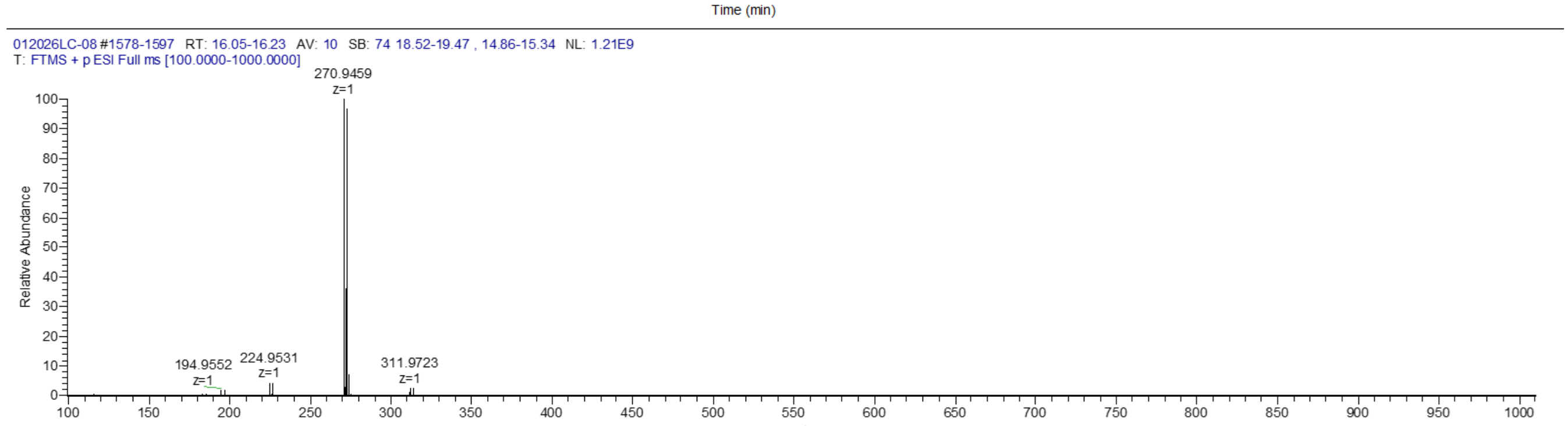

Supplement: Supplementary file 1 [file au6c00371_si_001.pdf]
